# Impact of intravenous lidocaine on clinical outcomes of patients with ARDS during COVID-19 pandemia (LidoCovid): A structured summary of a study protocol for a randomised controlled trial

**DOI:** 10.1186/s13063-021-05095-x

**Published:** 2021-02-11

**Authors:** Marie Muller, François Lefebvre, Marie-Line Harlay, Ludovic Glady, Guillaume Becker, Charlotte Muller, Ouafaa Aberkane, Mira Tawk, Madoé Julians, Arnaud Romoli, Stéphane Hecketsweiler, Francis Schneider, Julien Pottecher, Thiên-Nga Chamaraux-Tran

**Affiliations:** 1 Hôpital de Hautepierre, Service d’Anesthésie-Réanimation et Médecine Péri-Opératoire, Hôpitaux Universitaires de Strasbourg, Strasbourg, France; 2 Hôpital Civil, Service de Santé Publique, GMRC, Hôpitaux Universitaires de Strasbourg, Strasbourg, France; 3 Hôpital de Hautepierre, Service de Réanimation Médicale, Hôpitaux Universitaires de Strasbourg, Strasbourg, France; 4 Hôpital de Hautepierre, Laboratoire de Biochimie et Biologie Moléculaire, Hôpitaux Universitaires de Strasbourg, Strasbourg, France; 5 Hôpital de Hautepierre, Pôle Pharmacie-Pharmacologie, Hôpitaux Universitaires de Strasbourg, Strasbourg, France; 6 Laboratoire de Pharmacologie et Toxicologie Neurocardiovasculaire UR7296, Département Universitaire de Pharmacologie, Addictologie, Toxicologie et Thérapeutique, Université de Strasbourg, Strasbourg, France; 7 Vigilance des essais cliniques, Direction de la Recherche Clinique et de l’Innovation, Hôpitaux Universitaires de Strasbourg, Strasbourg, France; 8 Hôpital Civil, Direction de la Recherche Clinique et de l’Innovation, Hôpitaux Universitaires de Strasbourg, Strasbourg, France; 9 Faculté de Médecine, Fédération de Médecine Translationnelle de Strasbourg (FMTS), FRU 6702, EA3072, Université de Strasbourg, Strasbourg, France; 10 Institut de Génétique et de Biologie Moléculaire et Cellulaire (IGBMC), Illkirch, France; 11 Institut National de la Santé et de la Recherche Médicale (INSERM), Illkirch, U 1258 France; 12 Centre National de la Recherche Scientifique (CNRS), Illkirch, UMR 7104 France

**Keywords:** COVID-19, Randomised controlled trial, protocol, lidocaine, local anaesthetics, severe acute respiratory syndrome, molecular mechanisms of pharmacological action

## Abstract

**Objectives:**

The main objective of this study is to evaluate the effect of intravenous lidocaine on gas exchange and inflammation in acute respiratory distress syndrome due or not to Covid-19 pneumonia.

**Trial design:**

This is a prospective monocentric, randomized, quadruple-blinded and placebo-controlled superiority trial. This phase 3 clinical study is based on two parallel groups received either intravenous lidocaine 2% or intravenous NaCl 0.9%.

**Participants:**

This study has been conducted at the University Hospitals of Strasbourg (medical and surgical Intensive Care Units in Hautepierre Hospital) since the 4th November 2020. The participants are 18 years-old and older, hospitalized in ICU for a moderate to severe ARDS according to the Berlin definition; they have to be intubated and sedated for mechanical protective ventilation. All participants are affiliated to the French Social security system and a dosage of beta HCG has to be negative for women of child bearing age .

For the Covid-19 subgroup, the SARS-CoV2 infection is proved by RT-PCR <7 days before admission and/or another approved diagnostic technique and/or typical CT appearance pneumonia.

The data are prospectively collected in e-Case Report Forms and extracted from clinical files.

**Intervention and comparator:**

The participants are randomised in two parallel groups with a 1:1 ratio. In the experimental group, patients receive intravenous lidocaine 2% (20mg/mL) (from FRESENIUS KABI France); the infusion protocol provide a bolus of 1 mg/kg (ideal weight), followed by 3 mg/kg/h for the first hour, 1.5 mg/kg/h for the second hour, 0.72 mg/kg/h for the next 22 hours and then 0.6 mg/kg/h for 14 days at most or 24 hours after extubation or ventilator-weaning.

The patients in the control group receive intravenous NaCl 0.9% (9 mg/mL) (from Aguettant, France) as placebo comparator; the infusion protocol provide a bolus of 0.05 mL/kg (ideal weight), followed by 0.15 mL/kg/h for the first hour, 0.075 mL/kg/h for the second hour, 0.036 mL/kg/h for the next 22 hours, and the 0.03 mL/kg/h for up to 14 days or 24 hours after extubation or ventilator-weaning. Lidocaine level is assessed at H4, D2, D7 and D14 to prevent local anesthetics systemic toxicity.

Clinical data and biological samples are collected to assess disease progression.

**Main outcomes:**

The primary outcome is the evolution of alveolar-capillary gas exchange measured by the PaO2/FiO2 ratio after two days of treatment.

The secondary endpoints of the study include the following:

- Evolution of PaO2/FiO2 ratio at admission and after 21 days of treatment
- Number of ventilator-free days
- Anti-inflammatory effects by dosing inflammatory markers at different timepoints (ferritin, bicarbonate, CRP, PCT, LDH, IL-6, Troponin HS, triglycerides, complete blood count, lymphocytes)
- Anti-thrombotic effects by dosing platelets, aPTT, fibrinogen, D-dimers, viscoelastic testing and identification of all thromboembolic events up to 4 weeks.
- Plasmatic concentration of lidocaine and albumin
- Incidence of adverse events like cardiac rhythm disorders, need of vasopressors, any modification of the QRS, QTc or PR intervals every day
- Ileus recovery time
- Consumption of hypnotics, opioids, neuromuscular blockers.
- Lengths of stay in the ICU, incidence of reintubation and complications due to intensive care unit care (mortality until 90 days, pneumothorax, bacterial pneumopathy, bronchospasm, cardiogenic shock, acute renal failure, need of renal dialysis, delirium, atrial fibrillation, stroke (CAM-ICU score), tetraplegia (MCR score)).
- Incidence of cough and sore throat at extubation or ventilator-weaning and within 24 hours.

All these outcomes will be evaluated according to positivity to Sars-Cov-2.

**Randomisation:**

The participants who meet the inclusion criteria and have signed written informed consent will be randomly allocated using a computer-generated random number to either intervention group or control group. The distribution ratio of the two groups will be 1:1, with a stratification according to positivity to Sars-Cov-2.

**Blinding (masking):**

All participants, care providers, investigator and outcomes assessor are blinded.

**Numbers to be randomised (sample size):**

We planned to randomize fifty participants in each group, 100 participants total.

**Trial Status:**

The amended protocol version 2.1 was approved by the Ethics Committee “Comité de Protection des Personnes Sud-Méditerranée II on January 8, 2021 and by the Commission Nationale de l’Informatique et des Libertés (CNIL) on November 10, 2020. The study is currently recruiting participants; the recruitment started in November 2020 and the planned recruitment period is three years.

**Trial registration:**

The trial was registered on clinicaltrials.gov on October 30, 2020 and identified by number NCT04609865.

**Full protocol:**

The full protocol is attached as an additional file, accessible from the Trials website (Additional file 1). In the interest in expediting dissemination of this material, the familiar formatting has been eliminated; this Letter serves as a summary of the key elements of the full protocol.

**Supplementary Information:**

The online version contains supplementary material available at 10.1186/s13063-021-05095-x.

## Supplementary Information

**Additional file 1.**

## Data Availability

Not applicable.

