# Supplementary material for: Impact of intravenous lidocaine on clinical outcomes of patients with ARDS during COVID-19 pandemia (LidoCovid): A structured summary of a study protocol for a randomised controlled trial
Source: Trials. 2021 Feb 11;22:131. doi: 10.1186/s13063-021-05095-x (PMC7876973; doi:10.1186/s13063-021-05095-x)
Supplement: Supplementary file 1 — Additional file 1. [file 13063_2021_5095_MOESM1_ESM.pdf]

TITRE COMPLET de la recherche

Effets de la Lidocaïne intraveineuse dans le syndrome de détresse respiratoire aiguë dans le contexte de pandémie de COVID-19

Impact of Intravenous Lidocaine on clinical outcomes of patients with ARDS during COVID-19 pandemia

PRI 2020 HUS N°7820 - N° EudraCT 2020-002454-24

Titre abrégé : LidoCovid

Promoteur	**P. le Directeur Général,**
Hôpitaux Universitaires de Strasbourg<br>1, place de l'Hôpital,<br>67 091 STRASBOURG cedex<br>Tél. : 03 88 11 52 66<br>Fax : 03 88 11 52 40<br>Email : DRCI@chru-strasbourg.fr	**Le Directeur de la Recherche Clinique et des Innovations**<br>**Eric DEMONSANT**
**Investigateur Principal**<br><br>**Dr. CHAMARAUX-TRAN Thiên-Nga**<br>Service de Réanimation chirurgicale<br>Hôpital de Hautepierre<br>1 avenue Molière<br>67098 STRASBOURG Cedex<br>Tél. : 03 88 12 70 81<br>Email : thiennga.chamaraux-tran@chru-strasbourg.fr	**Signature de l'Investigateur Principal :**
**Méthodologiste responsable**<br><br>**Dr LEFEBVRE François**<br>Service de Santé Publique<br>Hôpital Civil<br>1 place de l'Hôpital<br>67091 Strasbourg<br>Tél. : 03 88 11 68 94<br>Email : francois.lefebvre@chru-strasbourg.fr	**Signature :**

Version N° : 2.1

Date de la version : 4 décembre 2020

TABLE DES MATIERES

HISTORIQUE DES MISES A JOUR DU PROTOCOLE 6

TABLE DES ABBREVIATIONS 8

RESUME DE L'ETUDE 11

I- INTRODUCTION ET JUSTIFICATION SCIENTIFIQUE DE L'ETUDE 17

I-1. Définition de la pathologie ou de l'événement étudié 17

I-2. Etat des connaissances scientifiques actuelles et questions restant en suspens 19

I-2.1 Prise en charge existante et ses limites 19
I-2.2. Hypothèse de la recherche 22
I-2.3- Rationnel pour l'utilisation du médicament expérimenté 23
I-2.4. Balance bénéfices / risques 31

II- OBJECTIF(S) ET CRITERES D'EVALUATION DE L'ESSAI 36

II-1. Objectif Principal 36

II-2. Objectifs Secondaires 36

II-3. Objectifs de l'étude ancillaire 36

II-4. Critère d'évaluation principal 36

II-5. Critères d'évaluation secondaires 37

II-6. Critères d'évaluation des études ancillaires 37

III- CONCEPTION DE LA RECHERCHE 38

III-1. Plan expérimental 38

III-2. Calendrier prévisionnel 38

IV- POPULATION ETUDIEE 38

IV-1. Critères d'inclusion 38

IV-2. Critères de non inclusion 39

IV-3. Faisabilité et modalités de recrutement 39

V- DEROULEMENT PRATIQUE DE L'ESSAI 40

V-1. Chronologie des visites 40

V-2. Description des visites 43
V-2.1 Inclusion en situation d'urgence (visite V0) 43
V-2.2 Visites de suivi (jusqu'à J28) 47
V-2.3 Mortalité à J60 48
V-2.4 Appel téléphonique à J90 (fin de recherche) 48
V-2.5 Visite de sortie d'étude en cas d'arrêt prématuré (le cas échéant) 48

V-3. Procédure de mise en insu et de levée d'aveugle 49
V-3.1 Mise en insu 49
V-3.2 Levée d'aveugle en cours de recherche 50
V-3.3 Levée d'aveugle à l'issue de la recherche 50

V-4. Arrêt prématuré 50
V-4.1 Critères et procédures d'arrêt prématuré de l'utilisation des produits expérimentaux 50
V-4.2 Critères et procédures d'arrêt prématuré de participation à la recherche 51
V-4.3 Critères d'arrêt d'une partie ou de la totalité de la recherche 51

V-5. Indemnisation 51

VI- MEDICAMENT EXPERIMENTAL 52

VI-1. Description et modalités d'administration 52

VI-2. Gestion des médicaments expérimentaux 53
VI-2.1 Libération et distribution des produits 53
VI-2.2 Fourniture des produits 53
VI-2.3 Conditionnement des produits 53
VI-2.4. Etiquetage des produits 53
VI-2.5 Expédition et gestion des produits 53
VI-2.6 Dispensation des produits 54
VI-2.7 Stockage 54
VI-2.8 Retour et destruction des produits non utilisés 54

VII - ETUDES BIOLOGIQUES 54

VII -1. Analyses biologiques prévues dans le cadre du protocole 54

VII -2. Constitution d'une collection d'échantillons biologiques 55

VIII - MEDICAMENTS/TRAITEMENTS AUXILIAIRES 55

IX- GESTION DES DONNEES DE SECURITE 56

IX -1. Définitions 56
IX -1.1 Evénement indésirable 56
IX -1.2 Effet indésirable 56
IX -1.3 Evénement ou effet indésirable grave 56
IX -1.4 Effet indésirable inattendu 57
IX -1.5 Fait nouveau 57

IX -2. Description des évènements indésirables attendus. 57

IX -3. Conduite à tenir par l'investigateur en cas d'évènement indésirable, de fait nouveau ou de grossesse. 60

IX -3.1 Recueil des évènements indésirables (EvI). 60
IX -3.2 Notification des évènements indésirables graves (EvIG), et des faits nouveaux 60
IX -3.3 Notification des grossesses 63
IX -3.4 Tableau récapitulatif du circuit des notifications 63

IX -4. Déclaration par le promoteur des suspicions d'effets indésirables graves inattendus, des faits nouveaux et autres évènements. 64

IX -5. Rapport annuel de sécurité. 64

X - CREATION D'UN COMITE DE SURVEILLANCE INDEPENDANT 65

XI- STATISTIQUES 65

XI-1. Description des Méthodes Statistiques Prévues 65

XI-2. Nombre de Personnes à Inclure et Justification 66

XI-3- Niveau de significativité statistique 67

XI-4. Critères statistiques d'arrêt de l'étude 67

XI-5. Modalités de prise en compte des données manquantes, non utilisées ou non valides 67

XI-6. Gestion des modifications apportées au plan statistique initial 68

XI-7. Choix des personnes à inclure dans les analyses 68

XI-8. Remplacement des sujets sortis d'étude 68

XII- DROIT D'ACCES AUX DONNEES ET DOCUMENTS SOURCE 68

XIII - RECUEIL DES DONNEES 69

XIII -1. Données sources 69

XIII -2. Cahier d'observation électronique (eCRF). 69

XIII -3. Base de données. 73

XIV - ASSURANCE DE LA QUALITE 73

XIV -1. Contrôle de cohérence. 73

XIV -2. Monitoring. 73

XV- ARCHIVAGE 74

XVI- CONSIDERATIONS ETHIQUES 74

XVI 1. Comité de Protection des Personnes et Autorité compétente 75

XVI -2. Information et consentement du volontaire ... 75

XVI -3. Constitution d'une collection d'échantillons biologiques ... 77

XVI -4. Protection des données à caractère personnel ... 77

XVI.5- Assurance ... 78

XVI -6. Financement ... 78

XVII - FIN DE RECHERCHE ET RAPPORT FINAL ... 78

XVIII - CONFIDENTIALITE ET PUBLICATION DES RESULTATS ... 79

XIX- BIBLIOGRAPHIE ... 80

XX- ANNEXES ... 90

ANNEXE 1 : QUESTIONNAIRE DE SANTE SF-361 MODIFIE (LIDOCOVID) ... 90

ANNEXE 2 : DEBIT LORS DE L'ADMINISTRATION DES TRAITEMENTS EN ML/H ... 95

HISTORIQUE DES MISES A JOUR DU PROTOCOLE

Version	Date	Raison de la mise à jour
V1.1	02/06/2020	Version soumise aux Autorités pour la demande d'autorisation initiale
V1.2	08/06/2020	Réponse au courriel émis le 5 juin 2020 par l'ANSM
V1.3	20/07/2020	Réponse au courriel émis le 10 juillet 2020 par l'ANSM et modifications recommandées par le CPP Sud Méditerranée II lors de l'émission de l'avis favorable du 05/06/2020 sur la version V1.1
V2.1	04/12/2020	La modification substantielle porte sur la mise à jour des sections relatives :<br>- aux critères d'éligibilité<br>- aux modalités d'information et de recueil du consentement<br>- à la prise en charge des patients ayant un SDRA consécutif à une infection par le coronavirus suite à la parution des dernières recommandations nationales et internationales.<br>- aux modalités de mise en œuvre de l'insu. Il est précisé que la vigilance essai clinique pourra lever l'insu en cas de SUSAR<br>- au recueil des évènements indésirables déterminants pour l'évaluation de la sécurité<br>- à la collection d'échantillons biologiques

EQUIPE SCIENTIFIQUE ASSOCIEE ET PLATEAUX TECHNIQUES* IMPLIQUES DANS L'ETUDE

QUALITE (statisticiens, chercheur associé, etc…)	NOM	PRENOM	SERVICE ou PLATEAUX TECHNIQUES ET ADRESSE DU LIEU	TELEPHONE	ADRESSE E-MAIL
PU-PH	Pr Chenard	Marie-Pierrette	Centre de Ressources Biologiques (CRB), Hôpital de Hautepierre<br>1 avenue Molière<br>67098 STRASBOURG Cedex		Marie-pierrette.chenard@chru-strasbourg.fr
Biologiste Responsable	BILLING	Michèle	UCBEC-Plateaux technique de biologie<br>Nouvel Hôpital Civil<br>1, place de l'hôpital<br>67091 STRASBOURG Cedex	0369550785	UCBEC@chru-strasbourg.fr
PH Biologiste médical	Dr Glady	Ludovic	LABORATOIRE DE BIOCHIMIE ET BIOLOGIE MOLECULAIRE<br>Hôpital de Hautepierre<br>1 avenue Molière<br>67098 STRASBOURG Cedex	03 88 12 87 95	ludovic.glady@chru-strasbourg.fr
Pharmacien	Dr Leveque	Dominique	SERVICE DE PHARMACIE - STERILISATION<br>Hôpital de Hautepierre<br>1 avenue Molière<br>67098 STRASBOURG Cedex	03 88 12 82 13	Dominique.Leveque@chru-strasbourg.fr
Interne	Muller	Marie	Service d'anesthésie-réanimation<br>Hôpital de Hautepierre<br>1 avenue Molière<br>67098 STRASBOURG Cedex		Marie.muller1@chru-strasbourg.fr
Méthodologiste - Statisticien	Dr Lefebvre	François	SERVICE DE SANTE PUBLIQUE<br>Hôpital Civil<br>1 place de l'Hôpital<br>67091 Strasbourg	03 88 11 68 94	francois.lefebvre@chru-strasbourg.fr

TABLE DES ABBREVIATIONS

ACo	Aides Cognitives
AC	Autorité Compétente
ACFA	Fibrillation Auriculaire
ACR	Arrêt CardioRespiratoire
AFOP	Acute Fibrinous and Organizing Pneumonia
Ag	Antigène
AL	Anesthésiques Locaux
ALRIV	Anesthésie LocoRégionale IntraVeineuse
AMM	Autorisation de Mise sur le Marché
ANI	Analgesia/Nociception Index
ANSM	Agence Nationale de Sécurité du Médicament et des produits de santé
anti-Xa	Activité anti-Xa
APACHE	Acute Physiology And Chronic Health Evaluation
AT	Aspirations Trachéales
AVC	Accident Vasculaire Cérébral
BIS	Indice BiSpectral
CAM-ICU	Confusion Assessment Method - Intensive Care Unit
CAMR	Comité « Analyse et Maitrise du Risque »
CFS	Clinical Frailty Scale
CJP	Critère de jugement principal
COREB	Coordination Opérationnelle Risque Epidémique et Biologique
CPOT	Critical-Care Pain Observation Tool
CPP	Comité de Protection des Personnes
CRF	Cahier d'observation
CRP	Protéine C Réactive
CSP	Code de la Santé Publique
CTL	Contrôle
DGOS	Direction Générale de l'Offre de Soins
DV	Ventilation en Décubitus Ventral
ECG	Électrocardiogramme
ECMO	ExtraCorporeal Membrane Oxygenation
eCRF	Cahier d'observation électronique
EIGI	Effet Indésirable Grave Inattendu
EL	Emulsion Lipidique
EMA	European Medicines Agency
EtCO2	End-tidal CO2
EvI	Evènement Indésirable
EvIG	Evènement Indésirable Grave
FiO2	Fraction inspirée en Oxygène
FR	Fréquence Respiratoire
GIHP	Groupe d'Intérêt en Hémostase Périopératoire

HCG	Human Chorionic Gonadotropin
HCSP	Haut Conseil de la santé Publique
HUS	Hôpitaux Universitaires de Strasbourg
ICAM	InterCellular Adhesion Molecule
ICH-GCP	International Conference on Harmonisation - GOOD CLINICAL PRACTICE
IDE	Infirmier Diplômé d'Etat
IFN	Interféron
IL	Interleukine
IMC	Indice de Masse Corporelle
IMG	Interruption Médicale de Grossesse
IOT	Intubation OroTrachéale
IVG	Interruption Volontaire de Grossesse
IVSE	intra veineux à la seringue électrique
KDIGO	Kidney Disease Improving Global Outcomes
LBA	lavages Bronchoalvéolaires
LDH	Lactate Deshydrogénase
LPS	Lipopolysaccharides
MAR	Médecin Anesthésiste Réanimateur
MCP	Monocyte Chemoattractant Protein
MI	Membres Inférieurs
MR001	méthodologie de référence 001
MRC	Medical Research Council
NaCl	Chlorure de Sodium
NFS	Numération Formule Sanguine
NF-κB	Nuclear Factor-kappa B
ONHD	Oxygénothérapie nasale à Haut Débit
OMS	Organisation Mondiale de la Santé
p38 MAPK	p38 Mitogen-Activated Protein Kinases
PaCO2	Pression partielle de dioxyde de carbone
PaO2	Pression artérielle en oxygène
PAVM	Pneumopathies Acquises sous Ventilation Mécanique
PCR	Polymerase Chain Reaction
PCT	Procalcitonine
PEEP (PEP)	Positive End-Expiratory Pressure (Pression Expiratoire Positive)
PHRC I	Programme Hospitalier de Recherche Clinique Interrégional
PIT	Poids Idéal Théorique
PRIS	Syndrome de Perfusion du Propofol
PUI	Pharmacie à Usage Intérieur
QTc	Intervalle QT corrigé
RASS	Richmond Agitation-Sedation Scale
RCP	Résumés des Caractéristiques du Produit
récepteur NMDA	récepteur acide N-Méthyl-D-Aspartique
RFE	Recommandations Formalisées d'Experts
ROX	Ratio of Oxygen Saturation

RPC	Recommandations pour la Pratique Clinique
RT-PCR	Reverse Transcription-Polymerase Chain Reaction
SAPS	Simplified Acute Physiology Score
SARS	Severe Acute Respiratory Syndrome
SAU	Service d'Accueil et de traitement des Urgences
SDRA	Syndrome de Détresse Respiratoire Aigu
SF36	Short Form (36) Health Survey
SFAR	Société Française d'Anesthésie et de Réanimation
SOFA	Sequential Organ Failure Assessment
SpO2	Saturation pulsée de l’hémoglobine en oxygène
SRLF	Société de Réanimation de Langue Française
TCA	Temps de Céphaline Activée
TCAr	ratio du Temps de Céphaline Activée
TDM	Tomodensitométrie
TNF	Tumor Necrosis Factor
TOF	Train Of Four
TP	Taux de Prothrombine
Tropo HS	Troponine T Haute Sensibilité
TSV	Tachycardie Supraventriculaire
USI	Unité de Soins Intensifs
VEGF	Vascular Endothelium Growth Factor
VNI	Ventilation Non Invasive
VSAI	Ventilation Spontanée avec Aide Inspiratoire
Vt	Volume courant
ZEEP	Zero end expiratory pressure (pression téléexpiratoire nulle)

RESUME DE L'ETUDE

**Titre de la recherche :** Effets de la Lidocaïne intraveineuse dans le syndrome de détresse respiratoire aiguë dans le contexte de pandémie de COVID-19
**Titre abrégé :** LidoCovid
**Promoteur :** Hôpitaux Universitaires de Strasbourg
**Investigateur coordonnateur/principal :** Dr. CHAMARAUX-TRAN Thiên-Nga
**N° HUS :** 7820
**N° EudracT :** 2020-002454-24
**Justification de la recherche :**<br>Un virus émergent de la famille des coronavirus, le SARS-CoV-2 a été identifié en Chine au mois de décembre 2019. La maladie provoquée par ce coronavirus, la COVID-19, peut provoquer une atteinte respiratoire grave, principale cause d'admission en réanimation, mettant en avant l'absence thérapeutique médicamenteuse du Syndrome de Détresse Respiratoire Aiguë (SDRA), un syndrome qui peut être rencontré dans la COVID-19 mais également dans d'autres pathologies. Le traitement du SDRA est symptomatique et consiste en une ventilation mécanique protectrice pendant plusieurs jours et malgré cela, la mortalité reste importante. Le pronostic des pneumopathies à Covid-19 quant à elle est principalement grevé par la forte inflammation, les problèmes thromboemboliques et la difficulté de sevrage ventilatoire avec risques nosocomiaux notamment les pneumopathies acquises sous ventilation mécanique (PAVM). D'autres problématiques logistiques se surajoutent comme la pression de places disponibles en service de réanimation, la pénurie de médicaments hypnotiques, morphiniques et de curarisation liée à l'hospitalisation massive de patients et du confinement qui atteint l'économie lors de la pandémie.<br>La Lidocaïne IV présente de multiples propriétés cliniques bien connues en période périopératoire qui pourraient dans le cadre du SDRA avoir un effet pour :<br>- Réduire les besoins en médicaments hypnotiques et morphiniques permettant une épargne de ces médicaments tant pour le patient que pour la société dans le contexte de risque de pénurie médicamenteuse<br>- Réduire la durée de l'iléus, importante dans ce type de population<br>- Réduire le taux d'épisodes thromboemboliques par ses propriétés antithrombotiques<br>Dans le contexte plus particulier de COVID-19, elle pourrait également :<br>- Contrôler l'orage cytokinique induit par la COVID-19 grâce à sa capacité à réduire le taux des cytokines proinflammatoires<br>- Réduire la toux au moment de l'extubation ou déventilation définitive et ainsi réduire le risque d'aérosolisation virale<br>- Avoir un effet neuroprotecteur et ainsi avoir une action contre les atteintes neurologiques de la COVID-19<br>Par ailleurs, des études précliniques ont montré une amélioration des paramètres cliniques et biologiques dans des modèles animaux de SDRA par la lidocaïne (diminution des lésions pulmonaires, des marqueurs de l'inflammation, de l'atteinte alvéolocapillaire, amélioration des échanges gazeux…).<br>Ces effets pléiotropes pourraient ainsi améliorer les patients d'un point de vue clinique et ainsi permettre un sevrage ventilatoire plus rapide et le transfert des patients plus rapidement vers un service de rééducation.<br>Cette étude serait la première étude clinique sur l'utilisation de lidocaïne, médicament couramment utilisé en période périopératoire, à visée thérapeutique dans le SDRA, dans le contexte de pandémie à COVID-19.
**Objectif principal :**<br>Evaluer l'efficacité de la Lidocaïne en IV sur le rapport PaO2/FiO2 à J2 chez les patients intubés ventilés pour SDRA modéré à sévère sans lien avec la Covid-19 et chez les patients intubés ventilés pour SDRA modéré à sévère consécutif à une infection par SARS-Cov-2
**Objectifs secondaires :**<br>1. Suivre l'évolution du rapport PaO2/FiO2 de J0 à J21 chez les patients intubés ventilés pour SDRA<br>2. Evaluer l'efficacité de la Lidocaïne en IV sur la durée de la ventilation mécanique chez les patients intubés ventilés pour SDRA<br>3. Mesurer les effets de la Lidocaïne IV sur les marqueurs de l'inflammation de routine

4. Evaluer l'effet antithrombotique de la Lidocaïne IV
5. Evaluer la concentration plasmatique de Lidocaïne IV chez les patients atteints de SDRA
6. Evaluer le retentissement hémodynamique de la lidocaïne IVSE
7. Evaluer l'efficacité de la Lidocaïne IV dans l'iléus de réanimation
8. Evaluer l'épargne morphinique et en hypnotique par la Lidocaïne IV
9. Evaluer l'impact de la Lidocaïne IV dans l'évolution et les complications des séjours en réanimation
10. Evaluer l'effet de la Lidocaïne IV sur la toux à l'extubation/déventilation
11. Comparer les résultats obtenus pour l'ensemble des objectifs en fonction de la positivité au SARS-Cov2.

Objectifs études ancillaires :

Une collection sera constituée afin de réaliser des recherches ultérieures sur la réponse inflammatoire dans le SDRA et la Covid-19

Critère d'évaluation principal :

Le critère d'évaluation principal sera le Rapport PaO_2/FiO_2 à J2 chez les patients intubés ventilés pour SDRA modéré à sévère sans lien avec la Covid-19 et chez les patients intubés ventilés pour SDRA modéré à sévère consécutif à une infection par SARS-Cov-2.

Critères d'évaluation secondaires :

1. Rapport PaO2/FiO2 de J0 à J21 (ou jusqu'à la sortie de réanimation en cas de sortie avant J21)
2. Nombre de jours vivants sans ventilation mécanique (ventilator-free days) à J28 et J90
3. Mesurer les effets de la Lidocaïne IV sur les marqueurs de l'inflammation de routine : dosage à J0, J2, J7, J14 et J21 de la ferritine, bicarbonate, CRP, PCT, LDH, IL-6, Tropo HS, Triglycérides, NFS avec lymphocytes
4. Evaluer l'effet antithrombotique de la Lidocaïne IV : dosage à J0, J2, J7, J14 et J21 des plaquettes, du TCAr, du Fibrinogène, des D-Dimères, recensement des évènements thromboemboliques jusqu'à J28. Tests thromboélastométriques (TEG6S® et/ou Quantra® à J0, J2, J7, J14 et J21)
5. Concentration plasmatique de la lidocaïne et albuminémie à H4, J2, J7, J14 ;
6. Recherche d'épisodes de troubles du rythme et recours à un vasopresseur, mesure du PR, du QRS et QTc quotidiennement jusqu'à J14
7. Evaluer l'efficacité de la Lidocaïne IV dans l'iléus de réanimation : date de reprise des gaz et du transit (selles)
8. Evaluer l'épargne morphinique en hypnotique par la Lidocaïne IV : collecte des posologies des hypnotiques, des curares et des morphiniques en mg/kg/h, 1 fois par jour, nombre d'épisodes nécessitant une curarisation
9. Evaluer l'impact de la Lidocaïne IV sur l'évolution du patient (réintubation, durée de séjour en réanimation) et sur les complications liées à la réanimation (mortalité à J7, J14, J21, J28, J60 et J90, épisodes de pneumothorax, de pneumopathie bactérienne, bronchospasme, choc cardiogénique, insuffisance rénale aigue, épuration extra-rénale, délirium, fibrillation atriale, AVC, tétraparésie de réanimation…). En ce qui concerne le délirium et la tétraparésie de réanimation, les scores CAM-ICU et MRC seront mesurés une fois par jour à partir du réveil du patient jusqu'à sa sortie de réanimation.
10. Evaluer l'effet de la Lidocaïne IV sur la toux en objectivant ou non une toux immédiatement ou dans les 24h après l'extubation/déventilation, et évaluer cet effet sur les maux de gorge immédiatement ou dans les 24h après l'extubation
11. Les critères présentés ci-dessus seront comparés en fonction de la positivité au SARS-Cov2

Critères d'évaluation études ancillaires :

Des marqueurs de l'inflammation seront dosés sur la collection d'échantillons biologiques constituée. Ces marqueurs seront définis en fonction de l'avancée des connaissances sur la pathologie étudiée.

Plan expérimental :

Etude monocentrique
Comparative
Randomisée
En double insu
Phase : 3

Critères d'inclusion :

- Homme ou femme âgé de 18 ans ou plus
- Hospitalisé en service de réanimation
- En SDRA modéré à sévère selon les critères de Berlin :
  - Présence dans les 7 jours suivant une pathologie pulmonaire ou extra-pulmonaire aigüe de l'association d'une hypoxémie aigüe ($PaO_2/FiO_2 \leq 300$ mmHg) chez un patient ventilé avec une pression expiratoire positive (PEP) de 5 cmH_2O au moins, ainsi que d'infiltrats radiologiques bilatéraux non entièrement expliqués par une insuffisance cardiaque ou une surcharge volémique.
  - La définition de Berlin distingue les SDRA selon le rapport PaO_2/FiO_2 en SDRA modérés ($100 < PaO_2/FiO_2 \leq 200$ mmHg) et SDRA sévères ($PaO_2/FiO_2 \leq 100$ mmHg).
- Intubé et sédaté pour ventilation mécanique protectrice
- Beta HCG négatif pour les femmes en âges de procréer
- Sujet affilié à un régime de protection sociale d'assurance maladie

Critères de non-inclusion :

- Hypersensibilité aux anesthésiques locaux du groupe à liaison amide
- Porphyrie aiguë
- Troubles de la conduction auriculoventriculaire nécessitant un entraînement électrosystolique permanent non encore réalisé
- Épilepsie non contrôlée par un traitement
- Traitement par fluvoxamine
- Traitement par un antiarythmique de classe III : amiodarone, dronedarone
- Traitement par un autre antiarythmique de classe I : quinidine, disopyramide, hydroquinidine, flécaïnide, propafenone
- Insuffisance hépatocellulaire définie par un TP<15% en l'absence de traitement par anti-vitamine K
- Sujet sous sauvegarde de justice
- Sujet sous tutelle ou sous curatelle (statut connu avant l'inclusion)
- Grossesse
- Allaitement

Déroulement pratique de l'essai :

<u>Inclusion (visite V0)</u>

- Recherche du consentement auprès du patient ou d'un proche
- Vérification des critères d'éligibilité
- Randomisation
- Bilan biologique : TCA, TCAr, gazométrie du sang artériel (PaO_2 et bicarbonates), plaquettes, Fibrinogène, Tropo HS, Tests thromboélastométriques (TEG6S® et/ou Quantra®), albumine, cholestérol, Triglycéride, ferritine, PCT, formule leucocytaire, d-dimères, IL-6, CRP, LDH
- Prélèvements spécifiques à l'essai (2 tubes héparinés de 2,5 ml) pour la constitution d'une collection d'échantillons biologiques
- Administration du traitement (lidocaïne/placebo)
- Surveillance respiratoire et hémodynamique

<u>4 heures après l'administration du traitement :</u>

Un examen clinique est réalisé à la recherche des troubles du rythme cardiaque et d'une hypotension.

Un prélèvement spécifique à la recherche est effectué afin de réaliser le dosage de la lidocaïne, il s'agit d'un tube hépariné de 2,5 ml.

<u>Visites de suivi (jusqu'à J28)</u>

Un suivi quotidien est effectué jusqu'à la sortie des patients de réanimation. Les examens réalisés sont ceux de la

pratique courante, ils comprennent :

- Un examen clinique (examen neurologique, auscultation cardiopulmonaire, examen vasculaire, examen urodigestif et examen cutané, surveillance hémodynamique et respiratoire (y compris ECG)
- Le bilan biologique standard :
  - Quotidiennement

    TCA, TCAr, gazométrie du sang artériel (PaO2 et bicarbonates), plaquettes, Fibrinogène
  - Bilan réalisé à J0, J7, J14 et J21

    Tropo HS, albumine, cholestérol
  - Bilan réalisé à J0, J2, J7, J14 et J21

    Triglycéride, ferritine, PCT, formule leucocytaire, d-dimères, IL-6, CRP, LDH, Tests thromboélastométriques (TEG6S® et/ou Quantra®),

Spécifiquement pour la recherche sont réalisés :

- Le dosage plasmatique de la lidocaïne H4, J2, J7 à J14
- Les prélèvements pour la constitution de la plasmathèque à J0, J2, J7 à J14

En cas de sortie du service de réanimation avant J28, on s'attachera à récolter les données de morbi mortalité auprès du médecin en charge du patient dans le service aval ou auprès du patient s'il est sorti d'hospitalisation.

Appel téléphonique à J60

Le patient sera contacté par téléphone à J60 par l'investigateur en charge de son suivi afin de recueillir son état de santé.

Appel téléphonique à J90 (fin de recherche)

Le patient sera contacté par téléphone à J90 par l'investigateur en charge de son suivi afin de recueillir son état de santé et lui fera passer une échelle de qualité de vie, il s'agit d'un questionnaire de santé SF-36 modifié, les questions 3 à 8 et 10 ne porteront pas sur les 4 dernières semaines mais sur la dernière semaine.

La participation du sujet à la recherche prend fin à l'issue de cette visite.

Visite de sortie d'étude en cas d'arrêt prématuré (le cas échéant)

En cas de sortie prématurée d'étude, le patient sera contacté par téléphone pour recueillir son état de santé ainsi que l'ensemble des données de sécurité (évènements indésirables).

Médicaments expérimentaux :

- **Lidocaïne :**

Le médicament de l'étude est la lidocaïne 2% sans conservateur (ATC N01BB02) fournie par le laboratoire FRESENIUS KABI France. Il sera administré dans une indication hors AMM. La voie intraveineuse est conforme au résumé des caractéristiques du produit. Le médicament est conditionné en ampoules de 10 ou 20 ml. Chaque ml de solution contient 20 mg de chlorhydrate de lidocaïne (forme monohydratée).
La solution de lidocaïne 20 mg/ml sera administrée par perfusion intraveineuse lente à l'aide d'un pousse seringue électrique.
Le protocole d'administration de la lidocaïne, afin de maintenir les taux plasmatiques inférieurs à 5µg/ml sera : un bolus de 1mg/kg de poids idéal théorique, puis 3 mg/kg/h pendant une heure, puis 1,5 mg/kg/h pour la 2ème heure, puis 0,72 mg/kg/h les 22 heures suivantes et 0,6 mg/kg/h après la 24ème heure, la perfusion est poursuivie jusqu'à 24h après extubation ou déventilation définitive ou jusqu'à 14 jours maximum. Ce protocole est basé sur des études pharmacologiques réalisées sur une population de 99 patients ayant bénéficié d'une chirurgie cardiaque pour pontage coronarien. A noter, l'administration de la lidocaïne se fera en poids idéal théorique.

- **Placebo**

Le placebo utilisé sera une solution de NaCl 0,9 % administrable par voie intraveineuse conformément à son AMM (ATC B05XA03). Le NaCl 0,9% est conditionné en ampoules de 10 ou 20 ml. Chaque ml de solution contient 9 mg de chlorure de sodium. La solution de NaCl 0,9% sera administrée par perfusion intraveineuse lente à l'aide d'un pousse seringue électrique.
Son administration sera l'équivalent volumétrique de la lidocaïne : un bolus de 0,05 ml/kg de poids idéal théorique, puis 0,15 ml/kg/h pendant une heure, puis 0,075 ml/kg/h pour la 2ème heure, puis 0,036 ml/kg/h les 22 heures suivantes et 0,03 ml/kg/h après la 24ème heure, la perfusion est poursuivie jusqu'à 24h après extubation ou déventilation définitive ou jusqu'à 14 jours maximum.
Afin de respecter la mise en insu, le médicament sera préparé extemporanément par un infirmier diplômé d'Etat qui n'est pas en charge du patient.

Création d'une collection d'échantillons biologiques :
Dans le cadre du présent protocole, une collection d'échantillon biologique sera nouvellement constituée : 2 tubes héparinés de 2,5mL de sang seront prélevés chez les sujets pour lesquels un consentement aura été recueilli. Ces prélèvements seront réalisés concomitamment aux bilans biologiques standards à J0, J2, J7 et J14 soit un total de 20 ml sur toute la durée de participation du sujet à l'étude.
Ces échantillons sanguins seront conservés pour une durée de 3 ans après la recherche au sein du Centre de Ressources Biologiques (CRB), Hôpital de Hautepierre, 1 avenue Molière, 67098 STRASBOURG Cedex.
Cette collection permettra la réalisation de recherche concernant la réponse inflammatoire lors de l'infection par le SARS-CoV-2.

Médicaments/traitements autorisés et/ou interdits :

- <u>Médicaments/Traitements autorisés :</u>

- Hypnotiques : propofol, kétamine, midazolam, clonidine, dexmétomidine ou tout hypnotique qui serait amené à substituer ceux-ci en cas de pénurie médicamenteuse
- Curares : cisatracurium ou tout curare qui serait amené à substituer celui-ci en cas de pénurie médicamenteuse
- Morphinique : sufentanil, rémifentanil ou tout morphinique qui serait amené à substituer ceux-ci en cas de pénurie médicamenteuse
- Les traitements prokinétiques et laxatifs
- Les traitements anticoagulants
- Les antibiotiques
- Les corticoïdes, notamment la dexaméthasone

- <u>Médicaments/Traitements interdits :</u>

- les autres anesthésiques locaux : ropivacaïne, lévobupivacaïne, chloroprocaïne notamment.
- antiarythmique de classe III : amiodarone, dronedarone
- autre antiarythmique de classe I : quinidine, disopyramide, hydroquinidine, flécaïnide, propafenone
- la fluvoxamine

Nombre de sujets nécessaire :
D'après les données d'une étude portant sur l'utilisation d'un protocole de sédation par sevoflurane dans le SDRA, le rapport PaO_2 sur FiO_2 est estimé à 205±56 dans le groupe avec sévoflurane et à 166±59 dans le groupe midazolam. Par conséquent, un effectif de 46 personnes par groupe permettra de montrer une différence de rapport PaO2 sur FiO2 avec une puissance de 90% et un risque de première espèce de 5%. Quatre patients sont ajoutés dans chaque groupe pour permettre de prévoir d'éventuelles sorties d'étude. Au total, 100 sujets seront donc inclus.
Il n'est pas possible de savoir la proportion de patients avec Covid-19, toutefois, cet effectif permettra d'obtenir au moins 50 sujets dans un sous-groupe (avec ou sans Covid-19) ce qui nous permettra d'obtenir une puissance d'au moins 65 % dans ce sous-groupe.
Les calculs ont été réalisés avec le logiciel R 3.6.3.

**Méthode statistique :**<br>Des régressions linéaires bayésiennes seront réalisées pour analyser les variables quantitatives, notamment le critère de jugement principal, et des régressions logistiques bayésiennes seront réalisées pour analyser les variables qualitatives.<br>Des analyses en sous-groupe et avec la présence d'une interaction entre le groupe de traitement et la positivité au Sars-Cov-2 seront également réalisées.
**Calendrier prévisionnel**<br>Durée de période d'inclusion : 3 ans<br>Durée de participation de chaque sujet : 3 mois<br>Durée totale de l'étude : 39 mois<br>Durée d'exclusion :<br>- pendant la recherche : le patient ne peut participer à un autre essai thérapeutique pendant la durée de sa participation à la présente recherche de 3 mois. La participation à un autre protocole de recherche observationnel est néanmoins possible.<br>- à l'issue de la recherche : pas de durée d'exclusion au bout des 3 mois de participation des sujets<br>- en cas de sortie prématurée : 1 semaine<br>Fin de la recherche : la fin de la recherche correspond à la dernière visite du dernier sujet participant à la recherche.

I- INTRODUCTION ET JUSTIFICATION SCIENTIFIQUE DE L'ETUDE

I-1. Définition de la pathologie ou de l'événement étudié

La pandémie de Covid-19 a mis un coup de projecteur particulier sur le Syndrome de Détresse Respiratoire Aigu (SDRA).

Un virus émergent de la famille des coronavirus, le SARS-CoV-2 a été identifié en Chine au mois de décembre 2019 (1). La maladie provoquée par ce coronavirus a été nommée COVID-19 par l'organisation mondiale de la santé (OMS), et ses manifestations cliniques sont variables allant du portage asymptomatique à une atteinte respiratoire grave avec un tableau de SDRA sévère (2,3). La propagation de ce virus hors de Chine a rapidement touché l'Europe et notamment la France à partir de février 2020 (4). A partir du 11 mars 2020, l'épidémie liée au SARS-CoV-2 est qualifiée de pandémie (5).

Les premières études rétrospectives montrent que la population touchée en Chine était représentée majoritairement par des hommes (62%), d'âge médian 56 ans, et présentant pour près de la moitié des cas au moins une comorbidité (diabète, hypertension artérielle et maladies cardio-vasculaires)(6). La mortalité était de 94% en cas de ventilation mécanique invasive. Or, la détresse respiratoire sur pneumopathie est la principale cause d'admission en réanimation des patients atteints de Covid-19. Initialement présenté comme un syndrome de détresse respiratoire aigu ou SDRA (3), le concept semble être remis en question par différents experts (7–9) mais son traitement symptomatique consiste toujours en une ventilation mécanique protectrice chez des patients intubés et sédatés en cas de mauvaise tolérance clinique, pendant plusieurs jours (7,10).

La définition la plus utilisée du SDRA est celle dite de Berlin (11). Le SDRA y est défini par la présence dans les 7 jours suivant une pathologie pulmonaire ou extra-pulmonaire aiguë de l'association d'une hypoxémie aiguë ($PaO_2/FiO_2 \leq 300$ mmHg) chez un patient ventilé avec une pression expiratoire positive (PEP) de 5 cmH_2O au moins, ainsi que d'infiltrats radiologiques bilatéraux non entièrement expliqués par une insuffisance cardiaque ou une surcharge volémique. La définition de Berlin distingue les SDRA selon le rapport PaO2/FiO2 en SDRA légers (200 < PaO2/FiO2 ≤ 300 mmHg), SDRA modérés (100 < PaO2/FiO2 ≤ 200 mmHg) et SDRA sévères (PaO2/FiO2 ≤ 100 mmHg).

Classiquement le SDRA est physiologiquement une atteinte alvéolaire diffuse constituée d'un œdème pulmonaire interstitiel et alvéolaire riche en protéines avec lésions endothéliales et épithéliales (atteinte des pneumocytes de type I et II) (12). Cet œdème est lié à l'augmentation de la perméabilité vasculaire, conséquence de l'inflammation ayant lieu au niveau de l'endothélium. Dans les lavages bronchoalvéolaires (LBA) des patients atteints de SDRA, on rencontre classiquement une augmentation de cytokines pro- et anti-inflammatoires (IL-1β, TNF-α, IL-6 et IL-10) et de leurs

agonistes et antagonistes (IL-1ra, sIL-1RII, sTNF-RI et -RII, et sIL-6R) (13). Dans les LBA des patients infectés par le SARS-Cov2, on retrouve également une augmentation des taux de plusieurs cytokines, notamment d'IL-1β, IL1-ra, IL-7, IL-8, IL-10, VEGF, TNF-α, MCP1, IFNγ etc. (14).

Les premières biopsies post-mortem des patients décédés d'insuffisance respiratoire aiguë sur Covid-19 montrent dans le premier stade de la maladie (ou phénotype L de Gattinoni) une infiltration lymphocytaire des alvéoles pulmonaires tandis que les stades plus avancés présentent une pneumonie fibrineuse et organisée aigue (AFOP pour *acute fibrinous and organizing pneumonia*) avec des dépôts de fibrine, une perte du tissu conjonctif avec des fibroblastes, un infiltrat de cellules T et une hyperplasie des pneumocytes de type 2 (8).

D'après l'étude LUNG SAFE, une étude internationale, multicentrique, prospective réalisée dans 50 pays et ayant inclus plus de 29 000 patients, les SDRA concernent 10,4% des patients de réanimation et 23,4% des malades ventilés. La mortalité est importante, de l'ordre de 40% environ et pouvant atteindre 45% en cas de SDRA sévère (15).

Le pronostic des pneumopathies à Covid-19 est principalement grevé par la forte inflammation en lien avec l'infection à COVID-19 (16,17), les problèmes thromboemboliques qui en découlent (18,19) et la difficulté de sevrage ventilatoire avec risques nosocomiaux notamment les pneumopathies acquises sous ventilation mécanique (PAVM). Par ailleurs, une atteinte neurologique, démasquée par un réveil très agité, avec atteinte cérébrale documentée en imagerie a été mise en évidence (20). La mortalité en réanimation en cas de COVID-19, notamment chez les patients ventilés est très disparate. Ainsi dans une des premières études chinoises publiées, en cas de ventilation mécanique, elle était de 97%. Dans une étude new-yorkaise, la mortalité chez les patients ventilés était de 88% (21). En France, l'étude COVID-ICU a pu montrer que chez 4 244 patients atteints de SDRA à SARS-CoV-2 admis en réanimation, 80% ont bénéficié de ventilation mécanique. La mortalité à 90 jours était de 31% dans toute la cohorte et de 37% lorsque les patients étaient ventilés le jour de leur admission en réanimation. Parmi ces patients ayant été intubés de manière précoce, la mortalité croissait avec la sévérité du SDRA (30%, 34%, et 50% pour les SDRA légers, modérés et sévères respectivement).

Enfin, la pandémie a fait apparaître d'autres problématiques, notamment d'ordre logistique. Ainsi s'est rajouté le risque de pénurie de médicaments hypnotiques, morphiniques et de curarisation (22) permettant l'adaptation à la machine ventilatoire de ces patients très hypoxémiques, présentant un « drive » respiratoire majeur. Malgré la diminution majeure du nombre de patients admis en réanimation, étant donné que les principes actifs sont majoritairement synthétisés en dehors de l'Europe, les médicaments d'anesthésie et de réanimation restent en tension et c'est l'Etat qui assure depuis le 25 avril 2020 l'approvisionnement des établissements de santé en médicaments dits

« prioritaires » à savoir deux hypnotiques : le Midazolam et le Propofol et trois curares : l'Atracurium, le Cisatricurium et le Rocuronium (Décret n° 2020-466 du 23 avril 2020 complétant le décret n° 2020-293 du 23 mars 2020).

Enfin, la pression de places disponibles en service de réanimation reste également problématique. Lors de la première vague, plus de 4806 lits de réanimation (+95%) ont été créés pour répondre à l'urgence sanitaire (23) et des transferts sanitaires entre régions ont eu lieu par train ou par les airs (24).

I-2. Etat des connaissances scientifiques actuelles et questions restant en suspens

I-2.1 Prise en charge existante et ses limites

Le SDRA n'est pas une maladie mais un regroupement syndromique défini par des variables cliniques et paracliniques. Ainsi, aucune thérapeutique médicamenteuse n'a fait preuve de son efficacité à ce jour (25) et la prise en charge se base sur une stratégie de ventilation mécanique protectrice basée sur une approche physiopathologique. Cette stratégie permet la limitation des lésions pulmonaires induites par la ventilation (ventilator-induced lung injury (VILI)) réduisant ainsi le volotraumatisme (réduction du stress et du strain). La limitation des pressions a l'avantage théorique de réduire la surdistension des zones aérées en préservant l'état hémodynamique.

Ainsi la prise en charge des SDRA se conforme aux recommandations formalisées d'experts (RFE) de 2018 de la SFAR endossant les recommandations internationales sur la prise en charge du syndrome de détresse respiratoire aiguë (SDRA) publiées en 2017 et réalisées par l'American Thoracic Society (ATS), l'European Society of Intensive Care Medicine (ESICM) et la Society of Critical Care Medicine (SCCM) (26) : la stratégie ventilatoire est de limiter les volumes courant (Vt 4-8 mL/kg de poids théorique) et la pression de plateau (Pplat < 30 cmH_2O), le décubitus ventral est appliqué au moins 12 h par jour en cas de SDRA sévère, la PEP appliquée est élevée (>10 cmH_2O).

En ce qui concerne la pneumopathie à Covid-19, plusieurs stratégies de diagnostic (PCR, imagerie), plusieurs stratégies ventilatoires (intubation précoce, le recours limité à la ventilation non invasive et à l'oxygénothérapie nasale à haut débit) et médicamenteuses (remdesivir, lopinavir/ritonavir, hydroxychloroquine, plasma thérapeutique) ont été proposées mais restent à ce jour fondées sur des données d'efficacité et de tolérance limitées dans la littérature, littérature qui est sans cesse renouvelée. Seuls les corticoïdes, en particulier la dexaméthasone, ont à ce jour pu démontrer leur efficacité sur la mortalité dans la COVID-19 (27,28).

En l'absence de consensus et en raison de données discordantes de la littérature concernant la prise en charge de la Covid-19, notre approche thérapeutique se fonde sur le protocole institutionnel (annexe 3), les Recommandations d'experts portant sur la prise en charge en réanimation des patients infectés à SARS-CoV2 pour la Coordination Opérationnelle Risque Epidémique et Biologique (COREB)(29), les recommandations du Haut Conseil de la Santé Publique (30) et des préconisations de la Survival Sepsis Campaign (31).

<u>I-2.1 a) Protocole diagnostique</u>

- PCR COVID : répéter la RT-PCR SARS-Cov2 à J7 (dans frottis + AT)
- Antigénurie légionnelle à l'admission
- Aspirations trachéales systématiques à l'admission (via système clos) : culture bactériologique
- PAS de LBA systématique à l'admission (souvent négatif initialement, risque de contamination, de dégradation de l'hématose). LBA à J5-J7 si dégradation.
- Hémocultures si fièvre (> 38,5°C)
- Recherche d'Aspergillose systématique en cours d'évaluation
- Seulement si dégradation clinique (J5-J7) : Culture + Ag Aspergillaire (sang) si dégradation respiratoire

<u>I-2.1 b) Protocole anti-infectieux</u>

- Traitement antiviral spécifique : Aucune preuve scientifique validée de l'efficacité des différentes molécules. Plusieurs essais thérapeutiques sont en cours en France. En attendant, en l'absence d'argument pour recommander une thérapeutique plutôt que l'abstention, cette dernière est à privilégier en dehors d'essais thérapeutiques, afin de s'affranchir de complications iatrogènes.
- Traitement antibiotique associé : NON SYSTEMATIQUE, seulement si des arguments sont en faveur d'une surinfection bactérienne
    - Céfotaxime CLAFORAN® 2g/8h pendant 7j
    - Spiramycine ROVAMYCINE® 1,5 MU/8h SEULEMENT si Antigénurie à légionnelle positive ou argument pour une surinfection à germe intracellulaire.

<u>I-2.1 c) Oxygénothérapie nasale à haut débit (ONHD) et ventilation non invasive (VNI)</u>

- Oxygénothérapie nasale à haut débit (ONHD) Optiflow®, avec masque chirurgical pour le patient (élargissement des indications, pas de limitation de débit)
    - Mesure du ROX (SpO_2/FiO_2/FR) à H2 (seuil 2,85), H6 (seuil 3,5) et H12 (seuil 3,8).

- Si ROX inférieur aux seuils précités ou non amélioré sous OHD maximale (50 à 60L/min et FiO_2 100%), l'intubation est légitime.

- VNI avec heaume (StarMed CaStar R Up®) ou masque de snorkeling modifié
- Envisager le décubitus ventral ou l'alternance des décubitus latéraux même en ventilation spontanée

I-2.1 d) Intubation

- Préoxygénation si nécessaire en VSAI : AI 10 cmH_2O, PEEP 5 cmH_2O, FiO_2=1 avec masque étanche AVANT d'allumer le respirateur
- Induction à Séquence Rapide
- Arrêt de la ventilation avant retrait du masque facial
- IOT par MAR entraîné, via vidéolaryngoscope (McGrath®)
- Filtre supplémentaire sur la branche expiratoire du respirateur, clamper la sonde d'IOT avant chaque débranchement, système clos pour aspirations trachéales

I-2.1 e) Ventilation mécanique

- Vt 6ml/kg PIT, recrutement alvéolaire, titration PEEP
- Curarisation si PaFi < 150 à discuter (bolus +/- IVSE selon réponse)
- Décubitus Ventral si PaO_2/FiO_2 < 100 (et à discuter quand le rapport PaO_2/FiO_2 est entre 100 et 150) d'une durée de 16h, 2 à 3 séances puis réévaluation
- Envisager d'enlever le raccord annelé et le capnographe pour réduire l'espace mort instrumental. Humidificateur chauffant NON systématique mais autorisé, notamment en cas de sécrétions collantes, sèches ou de signes d'obstruction de la sonde.

I-2.1 f) Sevrage

- Sevrage ventilatoire très progressif :
  - Arrêt curare seulement si PaO_2/FiO_2 > 200 et PEEP < 15 cmH_2O (en pratique, souvent après 48h de curarisation)
  - Consolider l'arrêt des curares au moins 24-48h avec sédation profonde sans modifier les paramètres respiratoires (aggravation très fréquente lors de l'arrêt des curares, et lorsqu'on introduit la VSAI trop précocement)
  - Baisser les sédations lorsque PaFi >150 avec PEEP<12 cmH_2O et FiO_2 <50%
- Epreuve de sevrage prolongée (1h, VSAI, AI 6 cm H_2O, ZEEP)
- Relais : oxygène aux lunettes

I-2.1 g) Sédation

- Propofol 2% IVSE : sans dépasser 4mg/kg/h pour éviter le PRIS
- Sufentanil 10µg/cc IVSE
- Place de la Kétamine dans la sédation après arrêt du curare à envisager (dans le cadre de ses propriétés de neuroprotection)

I-2.1 h) Nutrition

- *Nutrition entérale précoce : 2 Kcal/mL avec FIBRES pour limiter l'expansion volémique et améliorer la tolérance digestive en DV*
- *Macrogol FORLAX® systématique*
- *Erythromycine à visée prokinétique*

I-2.1 i) Thromboprophylaxie

- Anticoagulation préventive renforcée (enoxaparine 4000 UI 2x/j ou 6000 UI 2x/j si poids > 120 kg) si FDR thromboembolique (ex : syndrome inflammatoire important, IMC >30 kg/m2, ventilation mécanique)
- Anticoagulation curative selon critères cliniques et biologiques, durée adaptée au contexte clinique (exemple : survenue d'un évènement thromboembolique)
- Evaluer le risque thrombotique et hémorragique individuel (ATCD, traitements médicamenteux) ; adaptation thérapeutique selon la fonction rénale, arrêt si sortie d'hospitalisation ou indication spécifique.

I-2.1 j) antiinflammatoires

Dexaméthasone à la dose de 6mg/j pendant 10 jours (30).

I-2.2. Hypothèse de la recherche

Les anesthésiques locaux (AL) par leur action anti-inflammatoire ont démontré leur efficacité dans des modèles précliniques de SDRA (32). Et la Lidocaïne IV présente de multiples propriétés cliniques (reprises dans une revue (33,34)) qui pourraient dans le cadre du SDRA avoir un effet pour :

- Améliorer les échanges alvéolo-capillaires et réduire les résistances bronchiolaires du fait d'un effet myorelaxant sur l'arbre trachéobronchique (35)
- Réduire les besoins en médicaments hypnotiques (33,36–38), permettant une épargne tant pour le patient (facilitant ainsi son réveil) que pour la société dans le contexte de risque de pénurie médicamenteuse

- Réduire la durée de l'iléus, importante dans ce type de population (et réduite sous Lidocaïne IV en chirurgie)(39,40).

Dans le contexte de COVID-19, la lidocaïne pourrait avoir des effets intéressants pour :
- Contrôler l'orage cytokinique induit par la COVID-19 (17,41) grâce à sa capacité à réduire le taux des cytokines proinflammatoires démontrée dans le contexte de chirurgie digestive (42)
- Réduire le taux d'épisodes thromboemboliques par ses propriétés antithrombotiques(43,44,40)
- Réduire la toux au moment de l'extubation (45–47) ou déventilation définitive et ainsi réduire le risque d'aérosolisation virale (48)
- Avoir un effet neuroprotecteur qui pourrait protéger contre les atteintes neurologiques de la COVID-19 (49)

Ces effets pléiotropes pourraient ainsi améliorer les patients d'un point de vue clinique et ainsi permettre un sevrage ventilatoire plus rapide et permettre le transfert des patients plus rapidement vers un service de rééducation.

L'hypothèse que nous formulons est que l'utilisation de la lidocaïne intraveineuse comme adjuvant de l'analgo-sédation chez les patients en SDRA améliorerait les échanges alvéolocapillaires. L'effet attendu est donc l'amélioration du rapport PaO2/FiO2 à J2.

I-2.3- Rationnel pour l'utilisation du médicament expérimenté

La lidocaïne

Les anesthésiques locaux (AL) sont des agents pharmacologiques inhibant la transmission nerveuse par blocage des canaux sodiques au niveau de la moelle épinière, des ganglions spinaux, des nerfs périphériques, des plexus nerveux et des terminaisons nerveuses. Les AL sont des amines aromatiques, généralement des bases faibles en solution aqueuse.

Ce sont des molécules amphiphiles dont la structure de base est constituée :
- d'un cycle aromatique insaturé aux propriétés lipophiles
- d'un groupement amine (secondaire ou tertiaire) aux propriétés hydrophiles (50)
- d'une chaîne intermédiaire courte (2-3 C) aliphatique reliant le cycle aromatique et le groupement amine. Cette chaîne intermédiaire (liaison ester (-COO-) ou amide (NHCO-)) détermine la classe des anesthésiques locaux : amino-ester ou amino-amide.

Les différents éléments qui composent cette chaîne déterminent sa liposolubilité, ses propriétés de liaison aux protéines et son affinité au site d'action. Les structures du cycle aromatiques et du groupement amine déterminent les propriétés physico-chimiques des anesthésiques locaux.
Les AL amides ont moins d'effets secondaires que les AL esters, ceux-ci n'étant presque plus utilisés en France. Les molécules les plus employées sont : la lidocaïne, la mépivacaïne, la bupivacaïne et la ropivacaïne.

Décrite dès 1948 (51), la lidocaïne a plusieurs domaines d'application en anesthésie-réanimation : elle est utilisée comme anti-arythmique par voie systémique (52), pour la réalisation d'anesthésies locales et locorégionales (53–56) (ALRIV, infiltration, bloc nerveux périphérique, péridurales et rachianesthésie), comme analgésique lors des inductions anesthésiques et en peropératoire (57–59), comme traitement de la douleur chronique de type neuropathique (60,61). Nous nous intéresserons ici à ses mécanismes d'action par voie systémique.

Pharmacocinétique de lidocaïne

Les effets thérapeutiques, secondaires et toxiques de la lidocaïne dépendent de sa concentration plasmatique. Bien qu'il existe un chevauchement entre les concentrations thérapeutiques et celles où des effets secondaires peuvent être observés, la cible thérapeutique communément admise est une concentration plasmatique de lidocaïne entre 2 et 5 µg/mL, les effets secondaires subjectifs pouvant être observés entre 3 et 6 µg/mL et les effets toxiques aux alentours de 10 µg/mL chez la personne âgée (62). Les cas de cardiotoxicité ont été observés pour des concentrations plasmatiques de 21 µg/mL (34).
La diffusion de la lidocaïne se fait selon un modèle bi-compartimental où le compartiment central comporte la circulation sanguine et les organes nobles (foie, cœur, cerveau, poumon et rein) et le compartiment périphérique les organes moins bien perfusés (muscle, peau et graisse).
Avec une demi-vie de distribution très courte, inférieure à 10 minutes, la phase d'équilibre entre le plasma et le compartiment central est très rapide et détermine le délai d'action de la lidocaïne. La demi-vie d'élimination, entre 90 et 120 minutes est le reflet du métabolisme hépatique et de la redistribution du médicament. La biotransformation hépatique est la principale voie d'élimination. Soixante à 70% de la lidocaïne est éliminée par le premier passage hépatique. Ainsi, le métabolisme de la lidocaïne est dépendant du débit sanguin hépatique. Et bien qu'étant une base faible, avec une élimination urinaire pH-dépendante, la part rénale de son métabolisme est infime.

À partir des travaux de Rowland *et al.* et Thomson *et al.* sur la pharmacocinétique de la lidocaïne (63,64), Waller avait proposé deux équations permettant de calculer les doses de bolus et d'infusion continue de lidocaïne nécessaire à un sujet sain :

- DC (µg) = C_p (µg /mL) x V_c (mL/kg)
- DE (µg /min) = C_p (µg /mL) x Cl (mL/min/kg)

où DC est la dose de charge, C_p la concentration plasmatique, V_c le volume de distribution dans le compartiment central, DE la dose d'entretien et Cl la clairance de la lidocaïne.

Ainsi en partant d'un volume de distribution central d'environ 0,5 L/kg chez le sujet sain, une dose de charge de 1 mg/kg permet d'obtenir une concentration plasmatique de 2 µg/mL. Pour une clairance de 10 mL/min/kg, une dose d'entretien de 10 µg/kg/min permet de maintenir une concentration plasmatique de 1 µg/mL de lidocaïne. Afin d'obtenir rapidement une concentration plasmatique efficace, Waller propose l'association d'un bolus et d'une administration continue.

L'insuffisance cardiaque entraîne une diminution du volume de distribution et de la clairance plasmatique. De même, l'insuffisance hépatique prolonge la demi-vie d'élimination et diminue la clairance plasmatique de la lidocaïne. Ainsi, ces changements de paramètres pharmacocinétiques contre-indiquent l'utilisation systémique de la lidocaïne à visée analgésique chez les patients atteints de ces défaillances d'organes. S'il a été montré que ni le volume de distribution, ni la clairance plasmatique n'étaient atteints en cas d'insuffisance rénale, permettant l'utilisation de la lidocaïne aux mêmes posologies que chez les sujets sains dans certaines études (64), d'autres en revanche soulignent un allongement de la demi-vie d'élimination de la lidocaïne en cas d'insuffisance rénale sévère (CL_{cr} <30 mL/min · 1.73 m^{-2}) mais également une normalisation des taux en cas d'hémodialyse (65).

Mécanismes d'action de la lidocaïne

L'action analgésique de la lidocaïne intraveineuse est le fruit de ses multiples mécanismes d'action, résultant de son interaction avec les canaux sodiques et de ses effets directs ou indirects avec différents récepteurs et voies de transmission nociceptive.

Action classique de la lidocaïne sur les canaux sodiques périphériques et centraux

La transmission du stimulus nociceptif périphérique dépend de la présence des canaux sodiques voltage-dépendants. Ceux-ci peuvent être retrouvés sur les neurones sensitifs périphériques (NaV 1.8 et NaV 1.9) et sur les neurones sensitifs du système nerveux sympathique (NaV 1.7) (66). Il existe également un sous-type embryonnaire de canaux sodiques (NaV 1.3) dont la présence est décrite dans les neurones périphériques endommagés. Ce sous-type, surexprimé au niveau des lésions

périphériques, est associé à la douleur neuropathique et est la cible de la lidocaïne dans le traitement de la douleur chronique (67). Même à faible dose, la lidocaïne peut agir sur l'hyperalgésie centrale post-opératoire en bloquant les canaux sodiques résistants à la tétrodotoxine (sur les mécano-nocicepteurs) au niveau de la moelle épinière et des racines dorsales (68).

Mécanismes analgésiques passant par d'autres voies que celle des canaux sodiques

Administrée par voie intraveineuse, la lidocaïne entraîne une augmentation de la concentration d'acétylcholine dans le liquide cérébrospinal. Ceci permet d'obtenir une analgésie par inhibition de la douleur descendante par liaison aux récepteurs muscariniques M3 (69), inhibition des récepteurs à la glycine (70) et possible relargage d'opioïdes endogènes (71).

De plus, la lidocaïne agit au niveau de la moelle épinière en réduisant directement et indirectement la dépolarisation post-synaptique médiée par les récepteurs NMDA et neurokines (72). Dans des modèles animaux, la lidocaïne appliquée sur les racines nerveuses avant leur lésion, préviendrait les douleurs neuropathiques par une moindre libération d'aminoacides excitateurs (73) et une moindre activation des cellules microgliales (74). Ces propriétés pourraient ainsi avoir un effet préventif contre la confusion mentale en réanimation (delirium) (75,76).

Action de la lidocaïne sur les membranes phospholipidiques

Des études physico-chimiques ont montré que la lidocaïne avait un mode d'entrée différent de la bupivacaïne dans les membranes phospholipidiques, avec une affinité plus importante pour la face interne de la bicouche membranaire (77).

De fortes concentrations de lidocaïne peuvent également altérer la continuité des membranes cellulaires grâce aux propriétés détergentes des AL, analogues aux surfactants, causant notamment des lésions nerveuses irréversibles (78). Ce mécanisme peut également expliquer l'activité antimicrobienne concentration-dépendante de la lidocaïne sur les *Escherichia coli, Staphylococcus aureus, Pseudomonas aeruginosa, Candida albicans* (79) et *Streptococcus pneumonia* (80)et cette propriété pourrait être intéressante en prévention des surinfections bactériennes et les PAVM de manière générale ou sur pneumopathie à Covid-19.

Action antivirale

Dans des modèles *in vitro,* la lidocaïne a montré un effet protecteur contre l'invasion de cellules cérébrales par l'EHV-1 (Equine herpesvirus-1) (81) et permet de déstabiliser la structure de l'HSV-1 (Herpes Simplex Virus de Type 1) (82).

La lidocaïne comme adjuvant de l'analgo-sédation

Si les données de la littérature sont abondantes quant à l'utilisation de la lidocaïne intraveineuse en période périopératoire (83–85), il n'y a que peu de données à l'heure actuelle concernant l'utilisation de la lidocaïne intraveineuse à visée sédative en service de réanimation, si bien que les « Recommandations de pratiques cliniques pour la prévention et la gestion de la douleur, de l'agitation/sédation, de la confusion mentale, de l'immobilité et des altérations du sommeil chez les patients adultes en soins critiques » de la *Society of Critical Care Medicine* parues en septembre 2018 n'ont pu soutenir son utilisation en réanimation comme adjuvant aux morphiniques (86). Toutefois des données cliniques sont encourageantes. Dans une étude rétrospective concernant 21 patients admis en réanimation pour choc septique, défaillance cardiaque autre, respiratoire, neurologique ou complications postopératoires, l'administration de lidocaïne IVSE a permis la réduction des scores de douleur de plus de 20% et une réduction significative de la consommation de morphiniques. Trois patients ont présenté des manifestations neurologiques (diminution du score RASS, bourdonnements d'oreilles et agitation) résolutifs après arrêt de la lidocaïne. Cette étude n'avait pas de groupe contrôle et les doses administrées de lidocaïne étaient exprimées en mg/min (87). En anesthésie, plusieurs études montrent que la lidocaïne IVSE a un effet sur l'épargne en hypnotique, en effet pour une même dose d'hypnotique, l'administration de lidocaïne diminue les valeurs de l'indice bispectral (BIS), appareil de monitorage de la profondeur d'anesthésie (36–38) , dans une étude portant sur la sédation pour coloscopie, la lidocaïne IVSE a permis de réduire la consommation de propofol de près de 50% (88); cet effet pourrait être intéressant dans le contexte actuel de pénurie de médicaments d'anesthésie mais également à cause de la tachyphylaxie rencontrée chez les patients sédatés et atteints de COVID-19. En effet, les besoins en hypnotiques, notamment du propofol, des patients sont élevés et dans notre pratique, il a souvent été nécessaire d'avoir recours simultanément à plusieurs hypnotiques afin notamment d'être en-dessous du seuil des 4 mg/kg/h de propofol, dose à risque de « syndrome de perfusion du propofol » ou PRIS se présentant comme un état de choc réfractaire aux inotropes avec acidose lactique induisant une forte mortalité (89).

Propriétés anti-inflammatoires de la lidocaïne et action possible dans le SDRA

La lidocaïne intraveineuse réduit la réponse inflammatoire à l'ischémie tissulaire et atténue les dommages tissulaires induits par les cytokines endothéliales et vasculaires par le biais d'un mécanisme impliquant les canaux potassiques ATP-sensibles mitochondriaux (90) . Elle inhibe l'activation des polynucléaires neutrophiles, leur adhésion, démargination, chimiotactisme et sécrétion de médiateurs comme les cytokines (91,92). Elle agit également sur d'autres voies : prostaglandines, leucotriènes, thromboxanes, histamine (93)…

Ces propriétés ont été démontrées dans des modèles animaux, *in vitro* mais aussi en clinique (94). Dans un modèle *ex vivo* de macrophages de patientes atteintes ou non d'endométriose et de cellules stromales d'endométriomes, l'exposition à la lidocaïne entraîne un taux d'IL8 moins important des cellules stromales et une moindre expression génique l'IL-6, IL-8 et MCP1 par les macrophages des patientes non atteintes d'endométriose (le taux était en revanche inchangé) (95). Dans une étude où la lidocaïne IVSE permettait de diminuer la douleur post-opératoire après hystérectomie, l'analyse *ex vivo* des leucocytes a montré une diminution de la sécrétion d'IL-1ra et d'IL-6 et la préservation de la prolifération lymphocytaire après test à la phytohémagglutine-M (96). Une autre étude a également montré dans le même type de chirurgie une diminution de l'IFN-γ (97) Si elle n'a pas pu démontrer une efficacité contre les douleurs post-opératoires, la lidocaïne a montré une diminution plasmatique de plusieurs marqueurs de l'inflammation : IL-1, IL-6, IFN-γ et TNFα après cholecystectomie (42). Pour rappel, ces facteurs de l'inflammation sont retrouvés de manière élevée dans les LBA et le plasma des patients atteints de pneumopathies à Covid-19 (1,14). Ce sont les propriétés anti-inflammatoires des AL et plus particulièrement de la lidocaïne qui sont intéressantes dans le SDRA et les pneumopathies à COVID-19.

Ainsi, dans une revue de la littérature, Krishnamoorthy et Chung ont suggéré l'utilisation des anesthésiques locaux dans le traitement de l'inflammation dans le cadre du SDRA (32). Dans un essai *in vitro* sur cellules endothéliales et alvéolo-capillaires et dans un modèle in vivo de lésions pulmonaires induites par LPS chez le rat, Blumenthal *et al*. ont montré une diminution de l'expression de molécules d'adhésion (ICAM-1) *in vitro* et une moindre accumulation des neutrophiles et des cytokines *in vivo*, de même qu'une diminution de l'albumine présente dans les alvéoles (traduisant une réduction de la perméabilité alvéolo-capillaire anormale) quand les cellules ou le rat étaient exposés à la ropivacaïne (98). Dans leur conclusion, Krishnamoorthy et Chung préconisaient des études cliniques portant sur l'administration intratrachéale de ropivacaïne dans le SDRA. Plus récemment Piegeler *et al*. ont montré dans un modèle murin de lésions pulmonaires induites par LPS une diminution de l'eau extravasculaire pulmonaire lorsque les souris étaient exposées à la ropivacaine (99,100). De même lorsque les lésions étaient induites par LPS et ventilation mécanique à volumes courants élevés, les paramètres de lésions pulmonaires aigues étaient diminués : réduction de l'eau extravasculaire pulmonaire, de l'extravasation plasmatique, de l'index de perméabilité et de l'activité myéloperoxydase en cas d'exposition à la ropivacaïne. L'analyse par Western Blot des poumons montrait une diminution de l'activation des tyrosine kinases (src), de l'expression d'ICAM-1 et de la phosphorylation de la cavéoline-1, tous 3

marqueurs moléculaires de lésions pulmonaires, confirmant encore le rôle protecteur des AL dans l'inflammation du SDRA.

Les études précliniques concernant la lidocaïne portent quant à elles sur des modèles animaux de lapin, porc, rat :

- Dès 1994, Mikawa *et al*. ont montré que le prétraitement par lidocaïne IVSE diminuait les marqueurs inflammatoires de lésions pulmonaires aigues induites par endotoxine chez le lapin : réduction de la leucocytose, augmentation de la compliance pulmonaire, amélioration de la gazométrie artérielle, réduction de l'eau extravasculaire pulmonaire, réduction du nombre des cellules polynucléaires dans le LBA, réduction des taux d'albumine, de C3a, de C5a, de TNF alpha, d'IL1 bêta et de thromboxane B2 dans le LBA. Les lésions macroscopiques pulmonaires étaient également significativement diminuées (101).
- En 2000, la même équipe a montré des résultats similaires cette fois-ci dans un modèle de lésions induites par des enzymes pancréatiques (102).
- En 1996, cette équipe a montré que l'administration concomitante de lidocaïne IVSE à une ventilation à FiO_2 à 1 pendant 36h permettait de diminuer les lésions alvéolaires dues à l'hyperoxie (103).
- Enfin en 1998, cette équipe a montré que le prétraitement ou le traitement par lidocaïne IVSE après agression pulmonaire induite par acide chlorhydrique permettaient également de réduire ces lésions alvéolaires (104).
- Enfin, se basant sur ces travaux, une équipe américaine a utilisé la lidocaïne combinée au surfactant en administration intratrachéale dans un modèle de lésions pulmonaires induites par acide chlorhydrique chez des porcelets, ce traitement permettant une meilleur oxygénation et une meilleure compliance pulmonaire que le surfactant seul ou l'abstention thérapeutique (105).
- En 2010, l'équipe de Flondor a montré que la lidocaïne intraveineuse diminuait les taux d'IL-1β et de TNF-α dans le LBA dans un modèle d'endotoxémie chez le rat (106).
- Dans un modèle de volotraumatisme chez la souris, une équipe hollandaise a montré que la lidocaïne intraveineuse augmentait les taux plasmatiques et intrapulmonaire de la protéine anti-inflammatoire IL-10 et diminuait les besoins anesthésiques des animaux (94).
- Plus récemment, une équipe chinoise a montré que l'administration systémique de lidocaïne (par voie intrapéritonéale) dans un modèle de lésions pulmonaires induites par LPS chez le rat permettait de réduire l'eau extravasculaire pulmonaire et les lésions histopathologiques. De même, le TNF-α, IL-6 et MCP-1 étaient diminués dans le LBA et les analyses de biologie

moléculaire montraient une inhibition de la phosphorylation de NF-κB p65 et p38 MAPK, tous deux acteurs pivot de l'inflammation (107).

- En mars 2020, une étude chinoise a montré que les lésions pulmonaires induites dans un modèle d'endotoxémie chez le rat étaient atténuées par la lidocaïne administrée en intrapéritonéal en amont du LPS et que cet effet protecteur passait par une inhibition de HIF1α au niveau des macrophages (108).

Il n'existe à ce jour aucune étude clinique sur l'utilisation de lidocaïne à visée thérapeutique dans le SDRA. Son utilisation dans le cadre du présent protocole se fait donc hors AMM. Toutefois, son utilisation est sûre. Les 1ères publications concernant son utilisation en intraveineux remontent aux années 1950 (109) et la longue expérience de son utilisation comme agent anti-arythmique et les nombreuses études pharmacocinétiques permettent son utilisation en toute sécurité. Les données pharmacocinétiques ont été reprises dans une revue récente (34,33) qui rappelle que des concentrations plasmatiques comprises entre 1,4 et 6 µg/mL sont considérées comme sûres et efficaces. Le protocole d'administration de la lidocaïne dans cette étude se base sur des études pharmacologiques réalisées sur une population de 99 patients ayant bénéficié d'une chirurgie cardiaque pour pontage coronarien permettant d'obtenir une concentration plasmatique inférieure à 5 µg/mL (110). Compte-tenu de la sécurité d'emploi à la posologie de 1 à 1,5 mg/kg de lidocaïne "en bolus" en dose de charge (posologie de l'AMM), le protocole d'administration de la lidocaïne, afin de maintenir les taux plasmatiques inférieurs à 5µg/ml sera : un bolus de 1mg/kg de poids idéal théorique, puis 3 mg/kg/h pendant une heure, puis 1,5 mg/kg/h pour la 2ème heure, puis 0,72 mg/kg/h les 22 heures suivantes et 0,6 mg/kg/h après la 24ème heure jusqu'à 24 heures après extubation (ou déventilation définitive) ou 14 jours. La durée du traitement n'excèdera pas 14 jours, toutefois, il n'existe pas de durée maximale ou de dose cumulée maximale de lidocaïne dans la littérature.

Par ailleurs, du fait de ses propriétés antitussives, la lidocaïne a été mise en avant dans l'intubation et l'extubation des patients atteints de COVID-19 dans le contexte d'anesthésie pour chirurgie programmée ou en urgence afin de protéger les soignants (48,111). Elle possède également d'autres vertus sur la respiration qui peuvent être intéressantes dans le cadre de la pneumopathie à Covid-19. Tout d'abord, la lidocaïne IVSE n'a cliniquement qu'un faible effet dépresseur respiratoire (112) . *In vitro*, elle a des effets myorelaxants sur les cellules de muscles lisses de l'arbre trachéobronchique infectées par le SARS-CoV-2, et inverse l'effet contracturant de l'histamine ou de l'acétylcholine par inhibition des récepteurs muscariniques (69). Dans des modèles d'hyperréactivité bronchique, elle diminue la réponse à l'histamine que ça soit dans un modèle animal ou clinique (113–115). Enfin, la

lidocaïne est connue pour prévenir le laryngospasme au cours de l'anesthésie générale chez l'enfant (116).

I-2.4. Balance bénéfices / risques

- **Risques encourus :**

Comme tous les AL, la lidocaïne possède un effet « stabilisateur de membrane » pouvant avoir des répercussions graves sur le système nerveux central et cardiovasculaire. Cet effet est consécutif à un surdosage.

La prévention de la toxicité systémique passe donc par le respect des posologies et la surveillance continue du patient. La SFAR a publié plusieurs « recommandations formalisées d'experts » (RFE) (117) et « recommandations pour la pratique clinique » (RPC) (118) à ce sujet. Une quantité excessive d'AL ou une modification de la pharmacocinétique peuvent entraîner une intoxication à la lidocaïne. D'autres facteurs peuvent également être impliqués dans les mécanismes de toxicité (déséquilibre hydro-électrolytique, état de dénutrition avec hypoalbuminémie ou encore l'état de l'équilibre acidobasique).

Neurotoxicité des anesthésiques locaux

L'extraction des AL au niveau cérébral étant rapide, les signes neurologiques d'intoxication aux AL précèdent toujours les signes cardiaques (sauf en cas d'élévation très rapide des taux plasmatiques des AL).

Les premiers symptômes sont d'abord subjectifs : paresthésies péribuccales, goût métallique dans la bouche, acouphènes, vertiges. Puis apparaissent des signes objectifs : diplopie, tremblements des extrémités, logorrhée, confusion, myoclonies et convulsions (119). Les convulsions sont dues à une désynchronisation neuronale des structures corticales et sous corticales probablement liée à une diminution du tonus neuronal inhibiteur qui dépend surtout du système GABAergique et de la conductance chlorée (120). Un coma peut survenir à un taux plasmatique très élevé par blocage généralisé des canaux sodiques.

Cardiotoxicité des anesthésiques locaux

La toxicité cardiaque concerne la conduction et la contractilité cardiaques (119).

On peut observer des troubles de l'excitabilité et de la conduction, des arythmies, une diminution de la contractilité myocardique et une vasodilatation périphérique. Une bradycardie et un allongement de l'espace PR apparaissent en premier, suivis par des complexes QRS élargis. Apparaissent ensuite

des troubles du rythme à type de tachycardie et de fibrillation ventriculaire par un phénomène de réentrée. Les arythmies par réentrée sont favorisées en cas d'acidose ou de dyskaliémie.

Utilisation des solutions d'émulsion lipidique en cas d'intoxication systémique aux anesthésiques locaux

L'utilisation des solutions d'émulsion lipidique (EL) en 2006 a révolutionné la prise en charge des intoxications aux AL, qui, avant cette date, consistait en une réanimation symptomatique classique (121) .

Conduite à tenir en cas d'intoxication aux anesthésiques locaux

Forte de ces données expérimentales et de plusieurs cas cliniques ayant rapporté l'utilisation avec succès des émulsions lipidiques en cas de toxicité systémique aiguë des anesthésiques locaux, le comité « analyse et maitrise du risque » (CAMR) de la SFAR a mis à disposition une fiche de protocole concernant la prise en charge des intoxications aux anesthésiques locaux dans le cadre des aides cognitives (ACo) publiées sur son site internet (122).

Ainsi en cas de suspicion d'intoxication aux AL, se traduisant chez le patient non apte à communiquer par, au niveau neurologique, des convulsions, un coma ou, au niveau cardiaque par un bloc auriculoventriculaire ou des troubles du rythme ventriculaire par ou une hypotension, un collapsus ou un ACR, il faut :

- Arrêter l'injection des anesthésiques locaux
- Augmenter la FiO_2 à 1
- Débuter une réanimation cardio-respiratoire en cas d'ACR en évitant l'administration d'amiodarone ou de fortes doses d'adrénaline qui peuvent potentialiser l'effet toxique
- Le traitement spécifique consiste en l'administration d'émulsion lipidique à 20% (Intralipide® 20% : 3ml/kg en bolus ou Medialipide® 20% : 6 à 9ml/kg en bolus, à répéter si symptomatologie persistante)
- Des benzodiazépines seront administrées en cas de convulsions prolongées

La documentation de l'intoxication se fait par dosage de la cinétique des AL pendant et après résolution du choc.

Dans le cadre d'une prise en charge en service de réanimation, le patient bénéficie d'une surveillance par monitorage continu du tracé électrocardiographique, de la pléthysmographie et de la pression artérielle invasive permettant de déceler rapidement des signes de toxicité neurologiques ou cardiaques liés à la lidocaïne. De même, la surveillance biologique de la fonction hépatique (avec

taux de prothrombine) et le dosage de la lidocaïne à différents temps permet l'arrêt de l'administration du traitement en cas d'insuffisance hépatique (TP<15%), situation à risque de surdosage ou de taux plasmatique de lidocaïne >5µg/ml. Par ailleurs, dans les revues récentes concernant la lidocaïne IVSE en période périopératoire, il n'a pas été rapporté de cas de toxicité systémique (34,84).
En cas d'insuffisance rénale sévère (CL_{cr} <30 mL/min · 1.73 m^{-2}), en se basant sur l'étude pharmacocinétique de la lidocaïne de Demartin (65), il est préconisé de réduire les posologies de lidocaïne de moitié et de les rétablir en cas d'épuration extra-rénale.

Il est également rapporté dans les RCP de la lidocaïne des réactions d'hypersensibilité immédiate incluant le choc anaphylactique ou retardée. Ces réactions sont rares et seront rapidement détectées par le monitorage continu des patients, pour traitement relevant d'une prise en charge connue par les anesthésistes-réanimateurs. De même, de très rares cas de méthémoglobinémie ont pu être observés quel que soit l'âge mais plus particulièrement à craindre en période néonatale qui seront détectés par les gazométries artérielles, réalisées pluriquotidiennement.

Concernant les interactions médicamenteuses, elles concernent principalement les antiarythmiques (bêta-bloquants notamment). Leur association est possible en milieu de soins intensifs et de réanimation où la surveillance clinique est étroite et un contrôle de l'ECG est réalisé quotidiennement en plus du monitorage ECG continu avec monitorage du Qtc.
En cas de nécessité de recours à l'amiodarone pour ACFA mal tolérée, étant donné le risque d'augmentation des concentrations plasmatiques de lidocaïne, avec possibilité d'effets indésirables neurologiques et cardiaques, par diminution de son métabolisme hépatique par l'amiodarone, la surveillance clinique et ECG sera accrue. Le traitement par lidocaïne ou placebo en cas de traitement nécessaire par amiodarone sera définitivement arrêté.

Les antiviraux tels que le ritonavir / lopinavir qui, en raison de la probable interaction avec la lidocaïne, pourraient augmenter les taux sériques de la lidocaïne et sa toxicité potentielle (hypotension artérielle, arythmie cardiaques) ne sont plus utilisés dans le service du fait d'études négatives dans la COVID-19 (123) .

En ce qui concerne la cimétidine, elle n'a que peu d'indications en réanimation, les patients étant traités par inhibiteurs de la pompe à protons. En cas de recours à ce médicament, les doses habituelles sont de 200 mg donc en dessous du seuil d'interaction avec la lidocaïne.

En ce qui concerne la fluvoxamine, elle n'a pas d'indication en réanimation et est interrompue chez tout patient sédaté. Toutefois sa demi-vie est de 13 à 15 h après une prise unique et elle est plus longue (17 à 22 heures) en cas de prises répétées. De ce fait, les patients traités par fluvoxamine ne seront pas inclus dans l'étude.

- **Bénéfices escomptés :**

Réduire la durée de ventilation des patients en SDRA aura un bénéfice direct sur la survie des patients, la durée de la ventilation étant associée à un risque nosocomial important, notamment de pneumopathie acquise sous ventilation mécanique (124) et diminuera les complications découlant des séjours en réanimation. Par ailleurs, dans les Recommandations Formalisées d'Experts communes SFAR–SRLF de 2017 concernant les « Pneumonies Associées Aux Soins De Réanimation », les experts recommandaient dans le cadre d'une prévention multimodale des pneumonies associées aux soins de limiter les doses et les durées des sédatifs et analgésiques liées à la ventilation mécanique.

L'utilisation de la lidocaïne, molécule passée dans le domaine public, en stock important, pour réprimer l'orage cytokinique des patients pourrait être une alternative ou tout du moins un adjuvant aux traitements actuellement testés dans des protocoles de recherche, disponibles seulement en quantité limitée et chers notamment les immunomodulateurs (Tocizilumab par exemple) et un traitement antiinflammatoire complémentaire à la dexaméthasone (27).

Dans le cadre de ses effets anti-tussifs, la lidocaïne pourrait également diminuer les épisodes de toux post-extubation (125) et diminuer le risque d'aérosolisation virale et donc protéger les soignants (48).

Ses potentielles propriétés neuroprotectrices sur la microglie (126), recherchées dans plusieurs études notamment en chirurgie cardiaque (49,127) ou en neurochirurgie (128), pourraient également être bénéfiques dans l'atteinte neurologique des patients atteints de Covid-19 (20).

Enfin, réduire la durée de séjour en réanimation et la consommation de médicaments d'anesthésie est primordial devant les limites logistiques auxquelles notre système de santé doit actuellement faire face, notamment pour gérer la demande de lits de réanimation et les risques de pénurie en médicaments d'anesthésie (22,23,129) .

Ainsi, au vu des bénéfices importants pour le patient, les soignants et la société, et des risques minimes du fait du monitorage constant des patients en réanimation, le rapport bénéfice/risque est en faveur de l'utilisation de la lidocaïne dans le cadre de la sédation-analgésie pour SDRA.

II- OBJECTIFS ET CRITERES D'EVALUATION DE L'ESSAI

II-1. Objectif Principal

Evaluer l'efficacité de la Lidocaïne en IV sur le rapport PaO2/FiO2 à J2 chez les patients intubés ventilés pour SDRA modéré à sévère sans lien avec la Covid-19 et chez les patients intubés ventilés pour SDRA modéré à sévère consécutif à une infection par SARS-Cov-2.

II-2. Objectifs Secondaires

1. Suivre l'évolution du rapport PaO2/FiO2 de J0 à J21 chez les patients intubés ventilés pour SDRA
2. Evaluer l'efficacité de la Lidocaïne en IV sur la durée de la ventilation mécanique chez les patients intubés ventilés pour SDRA
3. Mesurer les effets de la Lidocaïne IV sur les marqueurs de l'inflammation de routine
4. Evaluer l'effet antithrombotique de la Lidocaïne IV
5. Evaluer la concentration plasmatique de Lidocaïne IV chez les patients atteints de SDRA
6. Evaluer le retentissement hémodynamique de la lidocaïne IVSE
7. Evaluer l'efficacité de la Lidocaïne IV dans l'iléus de réanimation
8. Evaluer l'épargne morphinique et en hypnotique par la Lidocaïne IV
9. Evaluer l'impact de la Lidocaïne IV dans l'évolution et les complications des séjours en réanimation
10. Evaluer l'effet de la Lidocaïne IV sur la toux à l'extubation/déventilation
11. Comparer les résultats obtenus pour l'ensemble des objectifs secondaires en fonction de la positivité au Covid-19.

II-3. Objectifs de l'étude ancillaire

Une collection sera constituée afin de réaliser des recherches ultérieures sur la réponse inflammatoire dans le SDRA et dans la pneumopathie à Covid-19.

II-4. Critère d'évaluation principal

Le critère d'évaluation principal sera le Rapport PaO_2/FiO_2 à J2 chez les patients intubés ventilés pour SDRA modéré à sévère sans lien avec le Covid-19 et chez les patients intubés ventilés pour SDRA modéré à sévère consécutif à une infection par SARS-Cov-2.

II-5. Critères d'évaluation secondaires

1. Rapport PaO2/FiO2 de J0 à J21 (ou jusqu'à la sortie de réanimation en cas de sortie avant J21)
2. Nombre de jours vivants sans ventilation mécanique (ventilator-free days) à J28 et J90
3. Mesurer les effets de la Lidocaïne IV sur les marqueurs de l'inflammation de routine : dosage à J0, J2, J7, J14 et J21 de la ferritine, bicarbonate, CRP, PCT, LDH, IL-6, Tropo HS, Triglycérides, NFS avec lymphocytes
4. Evaluer l'effet antithrombotique de la Lidocaïne IV : dosage à J0, J2, J7, J14 et J21 des plaquettes, du TCAr, du Fibrinogène, des D-Dimères, recensement des évènements thromboemboliques jusqu'à J28. Tests thromboélastométriques (TEG6S® et/ou Quantra® à J0, J2, J7, J14 et J21)
5. Concentration plasmatique de la lidocaïne et albuminémie à H4, J2, J7, J14 ;
6. Recherche d'épisodes de troubles du rythme et recours à un vasopresseur, mesure du PR, du QRS et QTc quotidiennement jusqu'à J14
7. Evaluer l'efficacité de la Lidocaïne IV dans l'iléus de réanimation : date de reprise des gaz et du transit (selles)
8. Evaluer l'épargne morphinique en hypnotique par la Lidocaïne IV : collecte des posologies des hypnotiques, des curares et des morphiniques en mg/kg/h, 1 fois par jour, nombre d'épisodes nécessitant une curarisation
9. Evaluer l'impact de la Lidocaïne IV sur l'évolution du patient (réintubation, durée de séjour en réanimation) et sur les complications liées à la réanimation (mortalité à J7, J14, J21, J28, J60 et J90, épisodes de pneumothorax, de pneumopathie bactérienne, bronchospasme, choc cardiogénique, insuffisance rénale aigue, épuration extra-rénale, délirium, fibrillation atriale, AVC, tétraparésie de réanimation…). En ce qui concerne le délirium et la tétraparésie de réanimation, les scores CAM-ICU et MRC seront mesurés une fois par jour à partir du réveil du patient jusqu'à sa sortie de réanimation.
10. Evaluer l'effet de la Lidocaïne IV sur la toux en objectivant ou non une toux immédiatement ou dans les 24h après l'extubation/déventilation, et évaluer cet effet sur les maux de gorge immédiatement ou dans les 24h après l'extubation.
11. Les critères présentés ci-dessus seront comparés en fonction de la positivité au Covid-19.

II-6. Critères d'évaluation des études ancillaires

Des marqueurs de l'inflammation seront dosés sur la collection d'échantillons biologiques constituée. Ces marqueurs seront définis en fonction de l'avancé des connaissances sur la pathologie étudiée.

III- CONCEPTION DE LA RECHERCHE

III-1. Plan expérimental

Il s'agit d'une étude monocentrique, comparative, randomisée, en double aveugle de phase 3.
Les patients sont pris en charge selon le protocole du service pour le SDRA et la pneumopathie à Covid-19. Lorsque les patients nécessitent une ventilation mécanique invasive, ils seront intubés selon le protocole du service et la prise en charge sera celle définie par le protocole du service.

Les patients seront recrutés en procédure d'urgence, et randomisés dans le groupe Lidocaïne ou contrôle dans les 24 h suivant l'intubation. Le sujet sera informé dès que possible et son accord sera recueilli pour la poursuite de la recherche.

III-2. Calendrier prévisionnel

Durée de période d'inclusion : **3 ans**
Durée de participation de chaque sujet : **3 mois**
Durée totale de l'étude : **39 mois**
Durée d'exclusion
- pendant la recherche : le patient ne peut participer à un autre essai thérapeutique pendant la durée de sa participation à la présente recherche**.** La participation à un autre protocole de recherche observationnel est néanmoins possible.
- à l'issue de la recherche : pas de durée d'exclusion au bout des 3 mois de participation des sujets
- en cas de sortie prématurée : **1 semaine**
Fin de la recherche *:* la fin de la recherche correspond à la dernière visite du dernier sujet participant à la recherche.

IV- POPULATION ETUDIEE

IV-1. Critères d'inclusion

- Homme ou femme âgé de 18 ans ou plus
- Hospitalisé en service de réanimation
- En SDRA modéré à sévère selon les critères de Berlin (11):

- présence dans les 7 jours suivant une pathologie pulmonaire ou extra-pulmonaire aigüe de l'association d'une hypoxémie aigüe ($PaO_2/FiO_2 \leq 300$ mmHg) chez un patient ventilé avec une pression expiratoire positive (PEP) de 5 cmH_2O au moins, ainsi que d'infiltrats radiologiques bilatéraux non entièrement expliqués par une insuffisance cardiaque ou une surcharge volémique.
- La définition de Berlin distingue les SDRA selon le rapport PaO_2/FiO_2 en SDRA modérés ($100 < PaO_2/FiO_2 \leq 200$ mmHg) et SDRA sévères ($PaO_2/FiO_2 \leq 100$ mmHg).

- Intubé et sédaté pour ventilation mécanique protectrice
- Beta HCG négatif pour les femmes en âges de procréer
- Sujet affilié à un régime de protection sociale d'assurance maladie

IV-2. Critères de non inclusion

- Hypersensibilité aux anesthésiques locaux du groupe à liaison amide.
- Porphyrie aiguë.
- Troubles de la conduction auriculoventriculaire nécessitant un entraînement électrosystolique permanent non encore réalisé.
- Épilepsie non contrôlée par un traitement.
- Traitement par un antiarythmique de classe III : amiodarone, dronedarone
- Traitement par un autre antiarythmique de classe I : quinidine, disopyramide, hydroquinidine, flécaïnide, propafenone
- Insuffisance hépatocellulaire définie par un TP<15% en l'absence d'anti-vitamine K
- Traitement par fluvoxamine
- Sujet sous sauvegarde de justice
- Sujet sous tutelle ou sous curatelle (statut connu avant l'inclusion)
- Grossesse
- Allaitement

IV-3. Faisabilité et modalités de recrutement

Le service de réanimation **chirurgicale** de L'Hôpital de Hautepierre prend en charge une trentaine de SDRA modéré à sévère par an. De même, une trentaine de patients sont admis par an dans le service de réanimation **médicale** de cet hôpital participant également à l'étude ce qui porte au nombre de 60 patients/an le potentiel d'inclusion dans la présente étude.

Par ailleurs, le Grand Est a particulièrement été touché lors de la première vague de la pandémie à COVID-19 et a connu un pic de 971 hospitalisations en réanimation au plus haut de la crise (dont 283 pour le Bas-Rhin), sachant que pour la même période, il y a eu 324 transferts de patients vers les autres régions et pays frontaliers moins touchés. Les inclusions pour la COVID-19 se feront ainsi au fil de l'eau et la durée de l'essai est de 3 ans afin de prendre en compte une saisonnalité du COVID-19, confirmée par cette deuxième vague. En cas de recrudescence du nombre de patients admis en réanimation, le recrutement de patients infectés par le coronavirus pourra représenter une part plus importante de la population étudiée.

V- DEROULEMENT PRATIQUE DE L'ESSAI

V-1. Chronologie des visites

Un tableau récapitule la chronologie des visites page suivante :

VISITES	Inclusion V0		Suivi	J2 (CJP)	Suivi journalier	J7	Suivi journalier	J14	Suivi journalier	J28	J60	J90 Visite de fin de recherche
						+/- 1J		+/- 1J				+/- 5J
Type de visite (consultation, HDJ, hospitalisation)	Hospit° J0	H4	Hospit°	Hospit°	Hospit°	Hospit°	Hospit°	Hospit°	Hospit°	Hospit°	Appel téléphonique	Appel téléphonique
Scanner thoracique / RT PCR pour confirmation infection par Sars-Cov-2	X											
Test RT PCR de suivi de l'infection par Sars-Cov-2						X		X	X(J21)	X		
Information et recueil de CE en procédure urgence	X*											
Données démographiques et antécédents médicaux	X											
Electrocardiogramme	X		X	X	X	X	X	X	X	X		
Vérification des critères d'éligibilité	X*											
Dosage des B HCG	X											
Randomisation	X*											
Administration de la lidocaïne versus placebo jusqu'à 24 h de l'extubation – déventilation maximum jusqu'à J14	X*	X*	X*	X*	X*	X*	X*	X*				
Examen clinique1	X	X	X	X	X	X	X	X	X	X		
Analyses biologiques2	X	X	X	X	X	X	X	X	X	X		

**Dosage plasmatique de la lidocaïne (Prélèvement d'un tube hépariné de 2,5 ml)**		**X***		**X***		**X***		**X***				
**Prélèvements sanguins plasmathèque (Prélèvement de 2 tubes héparinés de 2,5 ml)**	**X***			**X***		**X***		**X***				
**Echelle de qualité de vie**												**X***
**Recueil des événements indésirables**	**X***	**X***	**X***	**X***	**X***	**X***	**X***	**X***	**X***	**X***	**X***	**X***
**Recueil des traitements concomitants**	**X***	**X***	**X***	**X***	**X***	**X***	**X***	**X***	**X***	**X***	**X***	**X***

[1]Examen clinique quotidien :
Examen neurologique, auscultation cardiopulmonaire, examen vasculaire, examen urodigestif et examen cutané, surveillance hémodynamique et respiratoire
[2]Analyses biologiques : liste des examens biologiques

<u>Bilan **quotidien**</u>
Temps de céphaline activé (TCA) et son ratio TCAr, gazométrie du sang artériel (dont PaO2, Lactates et bicarbonates), plaquettes, Fibrinogène,
<u>Bilan réalisé à **J0, J7, J14 et J21**</u>
Tropo HS, albumine, cholestérol
<u>Bilan réalisé à **J0, J2, J7, J14 et J21**</u>
Triglycéride, ferritine, PCT, formule leucocytaire, d-dimères, IL-6, CRP, LDH, Tests thromboélastométriques (TEG6S® et/ou Quantra®)

Dosage plasmatique de la lidocaïne **H4, J2, J7 et J14**

Appel à J60 Statut vivant/décédé, retour à domicile, au travail, hospitalisation, réhospitalisation
Appel à J90 Statut vivant/décédé, retour à domicile, au travail, hospitalisation, réhospitalisation, et [3]échelle de qualité de vie (cf annexe 1).

(X) : Examens réalisés dans le cadre du soin
(X*) : Examens réalisés spécifiquement pour la recherche

V-2. Description des visites

V-2.1 Inclusion en situation d'urgence (visite V0)

Patients non aptes à consentir préalablement à leur inclusion :

Dans cette situation urgente, le consentement sera sollicité auprès d'un membre de la famille ou d'une personne de confiance. Dans le cas où cette personne ne peut pas être jointe, l'investigateur décidera d'inclure le patient dans l'étude au moyen d'une procédure de consentement en urgence. L'investigateur informera dans la mesure du possible le proche du patient de sa décision d'inclure le patient dans l'étude, son accord pour la poursuite de la recherche lui sera demandé, son consentement sera recueilli par écrit.

Le consentement éclairé différé sera obtenu dès que possible auprès du patient. Le patient sera informé du caractère entièrement volontaire de sa participation à cette recherche, ainsi que sur ses droits en matière de protection, d'accès, de rectification, d'effacement et de limitation de ses données personnelles. Ces informations sont communiquées oralement au patient et une notice d'information sera remise au sein du service où il est hospitalisé. Le sujet sollicité pourra contacter l'investigateur et poser toutes sortes de questions relatives à l'étude, auxquelles l'investigateur répondra. Son consentement écrit sera recueilli. La date à laquelle le sujet a accepté de participer à la recherche est notée dans son dossier médical, de même, que la date éventuelle d'opposition à sa participation, le cas échéant.

Le sujet est libre de refuser de participer à l'étude, il peut revenir à tout moment sur sa décision et retirer son accord et ce, quelle qu'en soit la raison, et ceci sans justification, sans conséquence sur la suite du traitement du patient ni la qualité des soins qui lui seront fournis et sans conséquence sur la relation avec son médecin.

Une copie du consentement renseignée et signée par l'investigateur sera mise dans le dossier médical du sujet ayant accepté de participer à l'étude.

Si l'investigateur découvre après inclusion en situation d'urgence que le patient est sous curatelle ou sous tutelle, alors il recueille le consentement du patient assisté par son curateur dans le cas d'une curatelle, ou il recueille le consentement du représentant légal dans le cas d'une tutelle.

Si tout au long de l'étude, l'investigateur n'a pas pu recueillir de consentement ni auprès du patient, ni auprès d'un proche, l'investigateur attestera par écrit avoir recherché un consentement auprès de ces derniers.

Vérification des critères d'éligibilité :
Les patients seront recrutés lorsque tous les critères d'éligibilité auront été vérifiés et que les résultats du test diagnostic Covid-19 auront été collectés.

Dans cette situation urgente, l'administration de la lidocaïne en adjuvant de la sédation doit être amorcée le plus rapidement possible, idéalement dans les 24h après intubation du patient s'il est en SDRA ou dans les 5 jours suivant le diagnostic de SDRA notamment s'il est déjà intubé.

Patients admis en réanimation sans être intubés, ni sédatés mais dont l'état se dégrade, et pour lesquels le consentement peut être recueilli préalablement à leur inclusion :
Dans les cas où l'insuffisance respiratoire aiguë est traitée initialement par oxygénothérapie haut débit ou VNI, les patients peuvent être informés et exprimer préalablement leur consentement pour participer à l'étude si jamais leur état se dégrade ultérieurement et nécessite une intubation et une sédation pour ventilation mécanique protectrice et qu'ils présentent alors les critères d'inclusion à l'étude.

Le consentement éclairé précède donc l'éligibilité du patient. Il permet d'informer le patient lorsque son état de santé le permet encore et lui laisse le temps de la réflexion. Il sera donc informé du caractère entièrement volontaire de sa participation à cette recherche, ainsi que sur ses droits en matière de protection, d'accès, de rectification, d'effacement et de limitation de ses données personnelles. Ces informations sont communiquées oralement au patient et une notice d'information sera remise au sein du service où il est hospitalisé. Le sujet sollicité pourra contacter l'investigateur et poser toutes sortes de questions relatives à l'étude, auxquelles l'investigateur répondra. Son consentement écrit sera recueilli. La date à laquelle le sujet a accepté de participer à la recherche est notée dans son dossier médical, de même, que la date éventuelle d'opposition à sa participation, le cas échéant.

Le sujet est libre de refuser de participer à l'étude, il peut revenir à tout moment sur sa décision et retirer son accord et ce, quelle qu'en soit la raison, et ceci sans justification, sans conséquence sur la

suite du traitement du patient ni la qualité des soins qui lui seront fournis et sans conséquence sur la relation avec son médecin.

Une copie du consentement renseignée et signée par l'investigateur sera mise dans le dossier médical du sujet ayant accepté de participer à l'étude.

Vérification des critères d'éligibilité :

Les patients qui ont donc consentis seront recrutés lorsque tous les critères d'éligibilité seront réunis (notamment une fois qu'ils seront intubés et sédatés) et que les résultats du test diagnostic Covid-19 auront été collectés.

Randomisation :

La randomisation s'effectue par l'investigateur (ou une personne déléguée) après signature du consentement et vérification des critères d'éligibilité. La randomisation permet l'attribution du numéro d'inclusion du sujet et de son groupe de traitement, soit :

- groupe contrôle : groupe placebo
- groupe expérimental : groupe lidocaïne

La randomisation conduira à l'inclusion du nombre de sujets nécessaire dans les groupes étudiés. Elle sera stratifiée sur la positivité au Sars-Cov-2. Elle se fera via Internet, par la plateforme Cleanweb, à laquelle accèdera l'investigateur ou une personne désignée, à l'aide de ses codes d'accès personnels. L'équipe investigatrice, ainsi que le promoteur recevront un mail de confirmation de la randomisation.

Examen clinique

Les examens réalisés sont ceux de la pratique courante, ils comprennent :

- Un examen clinique comportant un examen neurologique, auscultation cardiopulmonaire, examen vasculaire, examen urodigestif et examen cutané, surveillance hémodynamique et respiratoire (y compris ECG)
- La taille du patient sera vérifiée par mètre ruban.

Bilan biologique

- Un bilan biologique est réalisé de façon standard précédant l'administration du traitement : Temps de céphaline activé (TCA) et son ratio TCAr, gazométrie du sang artériel (PaO2 et bicarbonates), plaquettes, Fibrinogène, Tropo HS, Tests thromboélastométriques (TEG6S® et/ou

Quantra®), albumine, cholestérol, Triglycéride, ferritine, PCT, formule leucocytaire, d-dimères, IL-6, CRP, LDH

- Un prélèvement spécifique à l'essai, concomitant aux prélèvements de routine sur cathéter est effectué pour la constitution de la plasmathèque. Il s'agit de 2 tubes héparinés de 2,5 ml.

Administration du traitement à l'étude :

Dans chaque groupe, les patients recevront la stratégie thérapeutique attribuée lors de la randomisation jusqu'à l'extubation ou déventilation définitive du patient. L'équipe soignante de l'étude veillera à ce que l'échelle d'agitation-sédation de Richmond (RASS) soit de -4 à - 5 avant de commencer la sédation par propofol (à débuter à un débit de 2mg/kg/h de poids réel, +/- précédé d'un bolus) et que l'échelle Critical-Care pain Observation Tool (CPOT) soit inférieure à 3 par sufentanil (à 0,5mg/kg/h de poids réel, +/- précédé d'un bolus) et pendant la perfusion de cisatracurium dans les deux bras. Le blocage neuromusculaire, s'il n'est pas déjà instauré, doit commencer dans les 4 heures suivant la randomisation. Les patients recevront un bolus de cisatracurium de 15 mg, suivi d'une perfusion continue de 37,5 mg/heure pour un maximum de 48 heures. La dose peut être ajustée pour un TOF<2/4 ou synchronie avec le respirateur. La sédation, la curarisation et la ventilation (VT, FR et PEP) seront titrés pour une pression du plateau inspiratoire inférieure à 30 cmH_2O. En cas de dispositifs monitorant la profondeur d'anesthésie (BIS, entropie, Sedline) et/ou de l'analgésie (ANI), ceux-ci pourront être utilisés pour guider l'analgo-sédation. La curarisation se poursuivra idéalement jusqu'à ce que PaO_2/FiO_2 dépasse 150 pendant au moins 4 heures (ou pour une durée maximale de 48h). La sédation pourra ensuite être diminuée pour un score de RASS entre de 0 à -1, avec pour objectif une interruption de sédation rapide dans la mesure du possible. En ce qui concerne les patients atteints de COVID-19, ces derniers devront recevoir le ou les traitements de référence définis par le Haut Conseil de la santé Publique (HCSP). Pour rappel, selon l'avis relatif à l'actualisation des recommandations thérapeutiques dans le Covid-19 en date du 17 juin 2020 (128), la prise en charge actuelle consiste en la mise en place d'un traitement symptomatique de support (dit « Standard of Care »), l'abstention d'un traitement spécifique et l'inclusion prioritaire dans un essai thérapeutique. Cette prise en charge est susceptible d'évolutions, notamment lorsque les résultats des essais randomisés en cours seront disponibles.

Sont laissés à l'appréciation du praticien en charge du patient :

- En cas de détresse respiratoire, d'asynchronie respiratoire ou d'hypoxie, le recours à des doses plus élevées de sédation ou l'adjonction d'autres hypnotiques tels que le midazolam ou la kétamine

- Le recours au décubitus ventral et aux thérapies de sauvetage telles que l'ECMO
- La phase de sevrage ventilatoire et l'extubation sont guidées par les habitudes du service.

Le traitement par lidocaïne sera administré avec un bolus de 1mg/kg de poids idéal théorique, puis 3 mg/kg/h pendant une heure, puis 1,5 mg/kg/h pour la 2ème heure, puis 0,72 mg/kg/h les 22 heures suivantes et 0,6 mg/kg/h après la 24ème heure ou l'équivalent volumétrique en sérum physiologique dans le groupe CTL jusqu'à 24 h après l'extubation (ou déventilation définitive) du patient ou au maximum 14 jours. Le poids idéal théorique est calculé à partir de la taille et du sexe du patient selon la formule, P = X + 0,91 (taille en cm – 152,4), X = 50 pour les hommes et X = 45,5 pour les femmes.

La surveillance respiratoire et hémodynamique est accrue lors de l'instauration du traitement

4 heures après l'administration du traitement

- Un examen clinique est réalisé à la recherche des troubles du rythme cardiaque et d'une hypotension.
- Un prélèvement spécifique à la recherche est effectué afin de réaliser le dosage de la lidocaïne, il s'agit d'un tube hépariné de 2,5 ml. Ce prélèvement est effectué directement via le cathéter artériel déjà positionné. Seuls les tubes prélevés chez les sujets du bras lidocaïne seront analysés, tandis que les tubes prélevés pour les sujets du bras placebo alimenteront la collection biologique.

V-2.2 Visites de suivi (jusqu'à J28)

Un suivi quotidien est effectué jusqu'à la sortie des patients de réanimation.
Les examens réalisés sont ceux de la pratique courante, ils comprennent :

- Un examen clinique comportant un examen neurologique, auscultation cardiopulmonaire, examen vasculaire, examen urodigestif et examen cutané, surveillance hémodynamique et respiratoire (y compris ECG)
- Les bilans biologiques standards sont les suivants :

  Quotidiennement

  Temps de céphaline activé (TCA) et son ratio TCAr, gazométrie du sang artériel (PaO2 et bicarbonates), plaquettes, Fibrinogène.

  Bilan réalisé à J0, J7, J14 et J21

  Tropo HS, albumine, cholestérol

Bilan réalisé à J0, J2, J7, J14 et J21

Triglycéride, ferritine, PCT, formule leucocytaire, d-dimères, IL-6, CRP, LDH, Tests thromboélastométriques (TEG6S® et/ou Quantra®)

Spécifiquement pour la recherche sont réalisés :

- Le dosage plasmatique de la lidocaïne à H4, J2, J7 et J14 (bras lidocaïne)
- Les prélèvements pour la constitution de la plasmathèque à J0, J2, J7 et J14

En cas de sortie du service de réanimation avant J28, on s'attachera à récolter les données de morbi-mortalité auprès du médecin en charge du patient dans le service aval ou auprès du patient s'il est sorti d'hospitalisation.

Une attention particulière sera portée à J2, date de recueil du critère de jugement principal.

V-2.3 Mortalité à J60

A J60, l'investigateur s'enquerra de l'état clinique du patient afin d'évaluer l'efficacité et la sécurité de la lidocaïne dans le contexte de SDRA lié ou non à la COVID-19. Cette information sera récupérée par appel au service d'aval ou par appel téléphonique au patient ou à ses proches.

V-2.4 Appel téléphonique à J90 (fin de recherche)

Le patient sera contacté par téléphone à J90 par l'investigateur en charge de son suivi afin de recueillir son état de santé et lui fera passer une échelle de qualité de vie, il s'agit d'un questionnaire de santé SF-36 modifié, les questions 3 à 8 et 10 ne porteront pas sur les 4 dernières semaines mais sur la dernière semaine (cf annexe 1).
La participation du sujet à la recherche prend fin à l'issue de cette visite.

V-2.5 Visite de sortie d'étude en cas d'arrêt prématuré (le cas échéant)

En cas de sortie prématuré d'étude, le patient sera contacté par téléphone pour recueillir son état de santé et récolter l'ensemble des évènements indésirables.
Le traitement sera interrompu en cas d'entrée en état de choc avec insuffisance hépatocellulaire objectivée biologiquement, apparition d'épisodes de troubles du rythme ventriculaire secondaires à un trouble de la repolarisation ventriculaire.

La posologie du médicament sera diminuée de moitié en cas d'allongement du QTc supérieur à 500 ms.

Le reste de la prise en charge est laissé au libre arbitre des cliniciens en charge du patient.

La durée de suivi est de 90 jours, avec suivi téléphonique en cas de sortie de réanimation avant J28 et à 90 jours.

V-3. Procédure de mise en insu et de levée d'aveugle

V-3.1 Mise en insu

Préparation du traitement

Suite à la prescription médicale via une ordonnance essai clinique, le traitement sera préparé extemporanément par un(e) infirmier(e) diplômé(e) d'état ou par un médecin qui n'est pas en charge du patient en ouvert dans l'étude et qui attestera sur l'honneur ne pas divulguer le bras de traitement du patient dans l'étude. Etant donné le nombre d'intervenants et de rotation au sein d'un même service, la préparation du traitement se fera par un(e) infirmier(e) diplômé(e) d'état ou par un médecin d'un autre service. Pour le groupe « lidocaïne », le pousse seringue sera préparé à partir d'ampoules de lidocaïne pure pour un volume de 60 ml. Le débit sera calculé en fonction du poids théorique du patient. Pour le groupe « placebo », le pousse seringue sera rempli à partir de NaCl 0,9 %, avec un débit qui sera également défini en fonction du poids théorique du patient. La lidocaïne présente le même aspect que le NaCl si bien qu'il ne sera pas possible de distinguer le produit contenu dans le pousse seringue. L'étiquetage essai clinique du pousse seringue ne permettra pas d'identifier le médicament administré au patient. L'infirmier(e) diplômé(e) d'état (ou le médecin ayant préparé le pousse-seringue) assurera la traçabilité essai clinique du médicament administré au patient.

Contrôle des résultats du dosage de la lidocaïne

Afin d'éviter tout surdosage de lidocaïne, un dosage plasmatique de la lidocaïne sera réalisé à H4, J2, J7 et J14 et les résultats seront communiqués sous forme de valeur inférieure à 5 µg/ml, comprise entre 5 et 7,5 µg/ml ou supérieure à 7,5 µg/ml au médecin en charge du patient qui sera donc amené à modifier les débits d'administration du pousse seringue.

- En cas de taux plasmatique inférieur à 5 µg/ml, le débit d'administration reste le même
- En cas de taux plasmatique compris entre 5 et 7,5 µg/ml, l'administration du médicament est interrompue 3h et le débit sera ensuite diminué de 25%

- En cas de taux plasmatique au-delà de 7,5 µg/ml, l'administration du médicament est interrompue 10h et le débit sera ensuite repris à demi-dose.
- De manière aléatoire, afin de garder l'insu, de faux résultats seront également communiqués pour les patients du groupe contrôle.

Afin de ne pas mettre les investigateurs sur la voie du traitement, les débits de NaCl seront modifiés de façon aléatoire pour le groupe placebo le jour des dosages plasmatiques de lidocaïne (H4, J2, J7).

V-3.2 Levée d'aveugle en cours de recherche

En cas d'urgence, la levée d'insu pourra être effectué 24h/24h par un investigateur de l'étude via l'e-CRF cleanweb.
Conformément à la réglementation, le vigilant des essais cliniques en charge de l'étude sera autorisé à lever l'aveugle en cas de survenue d'un effet indésirable grave inattendu devant être transmis aux Autorités Règlementaires.

V-3.3 Levée d'aveugle à l'issue de la recherche

À l'issue de la recherche l'aveugle sera levée, et les sujets pourront être informés de leur bras de traitement dans l'étude sur simple demande.

V-4. Arrêt prématuré

V-4.1 Critères et procédures d'arrêt prématuré de l'utilisation des produits expérimentaux

Le traitement sera interrompu en cas d'entrée en état de choc avec insuffisance hépatocellulaire objectivée biologiquement avec un TP<15%, apparition d'épisodes de troubles du rythme ventriculaire secondaires à un trouble de la repolarisation ventriculaire.
La posologie du médicament sera diminuée de moitié en cas d'allongement du QTc supérieur à 500 ms ou d'insuffisance rénale sévère (CL_{cr} <30 mL/min · 1.73 m^{-2}). A noter, qu'en cas d'épuration extra-rénale, la posologie correspondant à une situation normale doit être reprise. En cas de surdosage médicamenteux objectivé par les dosages plasmatiques de H4, J2 ou J7, la posologie du médicament sera diminuée de la façon suivante :

- En cas de taux plasmatique inférieur à 5 µg/ml, le débit d'administration reste le même
- En cas de taux plasmatique compris entre 5 et 7,5 µg/ml, l'administration du médicament est interrompue 3h et le débit sera ensuite diminué de 25%

- En cas de taux plasmatique au-delà de 7,5 µg/ml, l'administration du médicament est interrompue 10h et le débit sera ensuite repris à demi-dose.
- De manière aléatoire, afin de garder l'insu, de fausses valeurs de lidocaïne pourront être communiquées pour les patients du bras contrôle.

Le reste de la prise en charge est laissé au libre arbitre des cliniciens en charge du patient.

V-4.2 Critères et procédures d'arrêt prématuré de participation à la recherche

Les sujets peuvent retirer leur consentement et demander à sortir de l'étude à n'importe quel moment quelle qu'en soit la raison. Il incombe à l'investigateur de documenter de façon aussi complète que possible les raisons de l'arrêt prématuré.
De son côté, l'investigateur est en droit d'interrompre temporairement ou définitivement la participation d'un sujet à l'étude, pour toute raison qui servirait au mieux les intérêts de ce dernier.

V-4.3 Critères d'arrêt d'une partie ou de la totalité de la recherche

Sur décision du CPP, du Promoteur ou de l'investigateur, il est possible d'interrompre l'étude (en cas de difficultés de recrutement, ou de tout autre motif le justifiant). Le traitement spécifique de la pneumopathie à COVID-19 peut varier au fur et à mesure de l'avancée des connaissances sur la maladie. S'il apparaissait qu'un traitement faisait la preuve de son efficacité dans le traitement du Covid-19 et était incompatible avec la lidocaïne, l'essai serait interrompu afin que les patients puissent bénéficier du meilleur traitement. Les inclusions seront donc interrompues. En ce qui concerne les patients déjà inclus dans l'étude, l'administration du médicament sera interrompue mais le suivi du patient avec les visites et le recueil des données seront poursuivis afin de permettre une analyse des données.

V-5. Indemnisation

Les sujets inclus dans la recherche ne percevront pas d'indemnité compensatoire pour leur participation à la recherche.

VI- MEDICAMENT EXPERIMENTAL

VI-1. Description et modalités d'administration

- Lidocaïne :

Le médicament de l'étude est de la lidocaïne 2% sans conservateur (ATC N01BB02) fournie par le laboratoire FRESENIUS KABI France. Il sera administré dans une indication hors AMM. La voie intraveineuse est conforme au résumé des caractéristiques du produit. Le médicament est conditionné en ampoules de 10 ou 20 ml. Chaque ml de solution contient 20 mg de chlorhydrate de lidocaïne (forme monohydratée).

La solution de lidocaïne 20 mg/ml sera administrée par perfusion intraveineuse lente à l'aide d'un pousse seringue électrique.

Le protocole d'administration de la lidocaïne, afin de maintenir les taux plasmatiques inférieurs à 5µg/ml sera : un bolus de 1mg/kg de poids idéal théorique, puis 3 mg/kg/h pendant une heure, puis 1,5 mg/kg/h pour la 2ème heure, puis 0,72 mg/kg/h les 22 heures suivantes et 0,6 mg/kg/h après la 24ème heure, la perfusion est poursuivie jusqu'à 24h après extubation ou déventilation définitive ou jusqu'à 14 jours maximum. Ce protocole est basé sur des études pharmacologiques réalisées sur une population de 99 patients ayant bénéficié d'une chirurgie cardiaque pour pontage coronarien (110).

A noter, l'administration de la lidocaïne se fera en poids idéal théorique.

Le poids idéal théorique est calculé à partir de la taille et du sexe du patient selon la formule, P = X + 0,91 (taille en cm – 152,4), X = 50 pour les hommes et X = 45,5 pour les femmes.

- Placebo

Le placebo utilisé sera une solution de NaCl 0,9 % administrable par voie intraveineuse conformément à son AMM (ATC B05XA03). Le NaCl 0,9% est conditionné en ampoules de 10 ou 20 ml. Chaque ml de solution contient 9 mg de chlorure de sodium. La solution de NaCl 0,9% sera administrée par perfusion intraveineuse lente à l'aide d'un pousse seringue électrique.

Son administration sera l'équivalent volumétrique de la lidocaïne : un bolus de 0,05 ml/kg de poids idéal théorique, puis 0,15 ml/kg/h pendant une heure, puis 0,075 ml/kg/h pour la 2ème heure, puis 0,036 ml/kg/h les 22 heures suivantes et 0,03 ml/kg/h après la 24ème heure, la perfusion est poursuivie jusqu'à 24h après extubation ou déventilation définitive ou jusqu'à 14 jours maximum.

Afin de respecter la mise en insu, le médicament sera préparé extemporanément par une infirmière diplômée d'Etat ou par un médecin qui n'est pas en charge du patient.

Les débits en mL/h sont ainsi les mêmes dans les deux groupes et sont repris dans le tableau en annexe 2. L'administration se fait jusqu'à 24 h après extubation (ou déventilation définitive si trachéotomie) ou 14 jours (la situation rencontrée le plus tôt).

VI-2. Gestion des médicaments expérimentaux

VI-2.1 Libération et distribution des produits

La forme commerciale de solution injectable de lidocaïne 2% (20 mg/ml) du laboratoire Fresenius Kabi sera commandée et distribuée par la PUI des HUS et fera l'objet d'un contre-étiquetage spécifique à l'étude conformément à la réglementation en vigueur. De la même façon, le chlorure de sodium PROAMP 0,9 % solution injectable du laboratoire Aguettant sera commandé, étiqueté et distribué par la PUI des HUS.

VI-2.2 Fourniture des produits

Le médicament expérimental sera fourni par la PUI des HUS.

VI-2.3 Conditionnement des produits

La lidocaïne 2% sera fournie conditionnée en ampoules de 10 ml ou de 20 ml. Le chlorure de sodium 0,9% sera fourni en ampoules de 10 ou 20 ml.

VI-2.4. Etiquetage des produits

Les ampoules de lidocaïne et de chlorure de sodium seront étiquetées conformément à l'arrêté du 24 mai 2006 fixant le contenu de l'étiquetage des médicaments expérimentaux. Une étiquette répondant aux exigences du même arrêté sera complétée par l'un(e) infirmier(e) diplômé(e) en charge de la préparation et apposé sur la seringue avant administration au patient.

VI-2.5 Expédition et gestion des produits

La fourniture des médicaments expérimentaux sera assurée par le pharmacien des essais cliniques de la pharmacie à usage intérieur. Un stock de lidocaïne sera délocalisé dans le service investigateur afin de pouvoir inclure des patients la nuit et le week-end. L'accès au stock sera restreint au personnel autorisé en ouvert.

L'utilisation de chaque ampoule fera l'objet d'une traçabilité nominative par le personnel désigné en ouvert.

Un renouvellement de la dotation sera effectué sur demande du service investigateur via un formulaire de demande de mise en dotation.

Comme indiqué dans le résumé des caractéristiques du produit de la lidocaïne 20 mg/ml, cette dernière ne nécessite pas de précautions particulières de conservation.

VI-2.6 Dispensation des produits

L'investigateur effectuera une prescription du traitement expérimental qui permettra de conserver l'insu. L'administration du traitement expérimental sera effectuée à partir du stock délocalisé dans le service. L'IDE en ouvert tracera nominativement le numéro de lot de chaque ampoule de Lidocaïne ou de NaCl 0,9% administrée à un patient. Chaque administration fera ensuite l'objet d'une traçabilité nominative qui sera tracée rétrospectivement par la pharmacie.

VI-2.7 Stockage

Les ampoules de lidocaïne et de chlorure de sodium seront conservées à température ambiante.

VI-2.8 Retour et destruction des produits non utilisés

Pour des raisons sanitaires, les ampoules et poches vides seront éliminées directement après administration via le circuit des déchets liés aux soins.

VII - ETUDES BIOLOGIQUES

VII -1. Analyses biologiques prévues dans le cadre du protocole

Bilan Standard

Les examens biologiques standards sont les suivants :

Un bilan quotidien est réalisé et comporte les analyses suivantes :

Temps de céphaline activé (TCA) et son ratio TCAr, gazométrie du sang artériel (dont PaO2, Lactates et bicarbonates), plaquettes, Fibrinogène.

Un Bilan sera réalisé **à J0, J7, J14 et J21** et comportera les analyses suivantes :

Tropo HS, albumine, cholestérol

Un Bilan sera réalisé à **J0, J2, J7, J14 et J21** et comportera les analyses suivantes :
Triglycéride, ferritine, PCT, formule leucocytaire, d-dimères, IL-6, CRP, LDH, Tests thromboélastométriques (TEG6S® et/ou Quantra®)

Examen réalisé spécifiquement pour la recherche :
Le dosage plasmatique de la lidocaïne sera effectué à **H4, J2, J7 et J14.** À cet effet, un tube hépariné de 2,5ml sera prélevé à **H4, à J2, à J7 et à J14.** Le dosage des échantillons des sujets du bras lidocaïne sera réalisé au sein du laboratoire de biochimie et biologie moléculaire, Hôpital de Hautepierre, 1 avenue Molière, 67098 STRASBOURG Cedex.

VII -2. Constitution d'une collection d'échantillons biologiques

Dans le cadre du présent protocole, une collection d'échantillon biologique sera nouvellement constituée : 2 tubes héparinés de 2,5mL de sang seront prélevés chez les sujets pour lesquels un consentement aura été recueilli. Ces prélèvements seront réalisés concomitamment aux bilans biologiques standards à J0, J2, J7 et J14 soit un total de 20 ml sur toute la durée de participation du sujet à l'étude.
Ces échantillons sanguins seront conservés à -80 °C pour une durée de 3 ans au sein Centre de Ressources Biologiques (CRB), Hôpital de Hautepierre, 1 avenue Molière, 67098 STRASBOURG Cedex.
Cette collection permettra la réalisation de recherche concernant la réponse inflammatoire lors de l'infection par le SARS-CoV-2.

VIII - MEDICAMENTS/TRAITEMENTS AUXILIAIRES

- **Médicaments/traitements auxiliaires* autorisés**

Les traitements auxiliaires autorisés sont :
- Hypnotiques : propofol, kétamine, midazolam, clonidine, dexmétomidine ou tout hypnotique qui serait amené à substituer ceux-ci en cas de pénurie médicamenteuse
- Curares : cisatracurium ou tout curare qui serait amené à substituer celui-ci en cas de pénurie médicamenteuse
- Morphinique : sufentanil, rémifentanil ou tout morphinique qui serait amené à substituer ceux-ci en cas de pénurie médicamenteuse
- Les traitements prokinétiques et laxatifs

- Les traitements anticoagulants
- Les antibiotiques
- Les corticoïdes, notamment la dexaméthasone

- **Médicaments/traitements interdits**

Les médicaments interdits sont les autres anesthésiques locaux : ropivacaïne, lévobupivacaïne, chloroprocaïne notamment) et les autres antiarythmiques de classe I (quinidine, disopyramide, hydroquinidine, flécaïnide, propafenone) et III (amiodarone, dronedarone), ainsi que la fluvoxamine.

IX- GESTION DES DONNEES DE SECURITE

IX -1. Définitions

IX -1.1 Evénement indésirable

Toute manifestation nocive survenant chez une personne qui se prête à une recherche impliquant la personne humaine que cette manifestation soit liée ou non à la recherche ou au produit sur lequel porte cette recherche.

IX -1.2 Effet indésirable

Evènement indésirable survenant chez une personne qui se prête à une recherche impliquant la personne humaine, lorsque cet évènement est lié à la recherche ou au produit sur lequel porte cette recherche.
L'effet indésirable d'un médicament expérimental, s'entend comme toute réaction nocive et non désirée à un médicament expérimental quelle que soit la dose administrée. Cette définition est également applicable aux préparations de thérapie cellulaire définies à l'article L. 1243-1 du code de la santé publique.

IX -1.3 Evénement ou effet indésirable grave

Tout évènement ou effet indésirable qui :
- entraine la mort,
- met en danger la vie de la personne qui se prête à la recherche,

- nécessite une hospitalisation ou une prolongation d'hospitalisation,
- provoque une incapacité ou un handicap important ou durable,
- se traduit par une anomalie ou une malformation congénitale,
- ou tout évènement considéré comme médicalement grave,

Et s'agissant du médicament quelle que soit la dose administrée

L'expression « mise en jeu du pronostic vital » est réservée à une menace vitale immédiate, au moment de l'évènement indésirable.

IX -1.4 Effet indésirable inattendu

Tout effet indésirable du produit dont la nature, la sévérité, la fréquence ou l'évolution ne concordent pas avec les informations de référence sur la sécurité mentionnées dans le résumé des caractéristiques du produit ou dans la brochure pour l'investigateur lorsque le produit n'est pas autorisé.

IX -1.5 Fait nouveau

- Toute nouvelle donnée pouvant conduire à une réévaluation du rapport des bénéfices et des risques de la recherche ou du produit objet de la recherche, à des modifications dans l'utilisation de ce produit, dans la conduite de la recherche, ou des documents relatifs à la recherche, ou à suspendre ou interrompre ou modifier le protocole de la recherche ou des recherches similaires.
- Pour les essais portant sur la première administration ou utilisation d'un produit de santé chez des personnes qui ne présentent aucune affection : tout effet indésirable grave.

IX -2. Description des évènements indésirables attendus.

Les évènements indésirables attendus liés à la prise en charge expérimentale :

Lidocaïne (groupe lidocaïne):

Les effets indésirables attendus liés à l'utilisation de la lidocaïne sont ceux mentionnés dans le Résumé des Caractéristiques du Produit (RCP) de LIDOCAÏNE KABI 20 mg/ml, solution injectable. Ce RCP mis à jour le 10/09/2018 constitue le premier document de référence sur la sécurité. Ils sont indiqués ci-dessous :

- Affections du système sanguin et lymphatique : Méthémoglobinémie (**fréquence indéterminée**)

- Affections du système immunitaire : Réactions d'hypersensibilité, urticaire, éruption cutanée, angio- œdème, bronchospasme, dans des cas graves, choc anaphylactique (**rare ≥ 1/10,000 à < 1/1,000**)
- Affections du système nerveux : Paresthésie, vertiges (**fréquent ≥ 1/100 à < 1/10**); symptômes de toxicité du SNC (convulsions, paresthésie péri-buccale, engourdissement de la langue, hyperacousie, troubles visuels, perte de conscience, tremblements, somnolence, étourdissement, acouphène, sensation d'intoxication, dysarthrie) (**peu fréquent ≥ 1/1,000 à <1/100**) ; Neuropathie, lésions des nerfs périphériques, arachnoïdite (**rare ≥ 1/10,000 à < 1/1,000**); nervosité, coma (**fréquence indéterminée**)
- Affections oculaires : Vision trouble, diplopie et amaurose transitoire, amaurose bilatérale (**rare ≥ 1/10,000 à < 1/1,000)**
- Affections de l'oreille et du labyrinthe : acouphènes, hyperacousie (**fréquence indéterminée**)
- Affections cardiaques : bradycardie (**fréquent ≥ 1/100 à < 1/10**) arrêt cardiaque, arythmies (**rare ≥ 1/10,000 à < 1/1,000**)
- Affections vasculaires : hypotension (**très fréquent ≥ 1/10),** hypertension (**fréquent ≥ 1/100 à < 1/10**)
- Affections respiratoires, thoraciques et médiastinales : dépression respiratoire (**rare ≥ 1/10,000 à < 1/1,000**), dyspnée, arrêt respiratoire (**fréquence indéterminée**)
- Affections gastro-intestinales : nausées (**très fréquent ≥ 1/10)**, vomissements (**fréquent ≥ 1/100 à < 1/10**)

Chlorure de sodium NaCl (groupe placebo):

Les effets indésirables attendus liés à l'utilisation du placebo-NaCl sont ceux mentionnés dans le Résumé des Caractéristiques du Produit (RCP) de CHLORURE DE SODIUM PROAMP 0,9 %, solution injectable. Ce RCP mis à jour le 26/09/2016 constitue le second document de référence sur la sécurité.

Le principal risque est celui d'un surdosage. Les effets indésirables généraux de l'excès de sodium comprennent nausées, vomissements, diarrhée, crampes abdominales, soif, diminution de la sécrétion de salive et de larmes, sudation, fièvre, tachycardie, hypertension, insuffisance rénale, œdème pulmonaire et périphérique, arrêt respiratoire, céphalées, étourdissement, impatience, irritabilité, lipothymie, contraction et raideur musculaire, convulsions, coma et décès.

Les signes cliniques du syndrome de démyélinisation osmotique sont progressifs : confusion, dysarthrie, dysphagie, faiblesse des membres, puis tétraplégies, délire et finalement coma. Les symptômes cliniques surviennent plusieurs jours après une trop rapide et/ou trop importante correction de l'hyponatrémie.

L'administration excessive de chlorure de sodium peut provoquer une hypernatrémie nécessitant l'arrêt immédiat de la solution de chlorure de sodium et doit être traitée par un médecin spécialiste. Ce traitement consiste en la surveillance de la natrémie et en l'administration de solution pour perfusion de glucose. Les chlorures en excès dans l'organisme peuvent provoquer une perte de bicarbonate avec une acidose.

Etant donné que la natrémie, le pH sanguin et le taux de bicarbonate plasmatique sont des paramètres biologiques dosés et corrigés plusieurs fois par jour en réanimation, les risques surajoutés du fait de ce traitement sont nuls.

Les évènements indésirables attendus liés à la recherche sont les suivants :

- les effets indésirables attendus sont ceux mentionnés dans le résumé des caractéristiques du produit en vigueur de la spécialité concernée ou du chef de groupe générique le cas échéant
- En cas d'association au propofol, une augmentation de l'effet hypnotique

En effet, en cas d'association à la lidocaïne, un effet hypnotique accru du propofol est attendu ce qui permettra de diminuer la posologie du propofol (épargne en hypnotique). A noter, qu'une diminution de l'aire sous la courbe de la lidocaïne a été mise en évidence en cas d'association au midazolam

- Une potentialisation de l'effet de blocage neuromusculaire des curares (épargne en curares).
- Un risque de majoration de la dépression respiratoire due aux opioïdes
- Afin de limiter ces effets, la sédation est ajustée au RASS, la curarisation au TOF et l'analgésie au CPOT.
- Prélèvements sanguins : il s'agit d'un volume sanguin supplémentaire, aucun risque n'est attendu

Les évènements indésirables attendus liés à l'évolution de la maladie sont les suivants :

- Décès,
- Trachéotomie et assistance ventilatoire permanente,
- Oxygénothérapie permanente,
- Recours à l'ECMO,
- Insuffisance rénale nécessitant ou non une épuration extra-rénale

- Évènements thromboemboliques
- Pneumopathies acquises sous ventilation mécanique
- Confusion mentale

Tout évènement indésirable ne figurant ni dans la liste des évènements attendus ni dans la dernière version du document de référence est qualifié d'inattendu ((RCP) de LIDOCAÏNE KABI 20 mg/ml, solution injectable mis à jour le 10/09/2018 et RCP du 26/09/2016 du CHLORURE DE SODIUM PROAMP 0,9 %, solution injectable.)

IX -3. Conduite à tenir par l'investigateur en cas d'évènement indésirable, de fait nouveau ou de grossesse.

IX -3.1 Recueil des évènements indésirables (EvI).

Dès la signature du consentement, l'investigateur est responsable du recueil de tous les évènements indésirables. Il rapporte les évènements indésirables graves et non graves (EvI biologiques et cliniques) qui surviennent entre la signature du consentement et la fin de participation du patient ou la fin de recueil des évènements indésirables, dans le cahier d'observation (CRF).
Ces évènements indésirables seront évalués à chaque visite au cours de l'étude par un interrogatoire et lors de l'examen clinique du patient.
L'évolution clinique des patients inclus dans cette étude sera marquée par des modifications des paramètres biologiques, des signes et symptômes liés à la pathologie sous-jacente (patient hospitalisé en réanimation). Compte-tenu du profil de sécurité bien connu de la lidocaïne, seuls les évènements indésirables déterminants pour l'évaluation de la sécurité seront notifiés par l'investigateur et concernent :

- Evènements respiratoires, thoraciques et médiastinaux
- Evènements lié à un surdosage de lidocaine
- Evènements thromboemboliques
- Troubles du rythme

IX -3.2 Notification des évènements indésirables graves (EvIG), et des faits nouveaux

L'investigateur évalue chaque évènement indésirable au regard de sa gravité. La gravité est déterminée conformément à la définition « évènement ou effet indésirable grave » cf supra.
La gradation de la sévérité, si pertinente, se fera à partir de l'échelle suivante :

- Grade 1 : légère, asymptomatique ou symptômes légers, observation clinique ou diagnostique seulement, intervention non indiquée
- Grade 2 : modérée, intervention minime, locale ou non invasive indiquée, limitant les activités instrumentales de la vie quotidienne appropriées à l'âge (préparation des repas, utilisation du téléphone, gestion comptable, …)
- Grade 3 : sévère ou médicalement significatif mais sans mise en jeu du pronostic vital immédiate, nécessitant une hospitalisation ou prolongation d'hospitalisation, invalidant, limitant les soins personnels de la vie quotidienne (baignade, habillement et déshabillage, alimentation, utilisation des toilettes, …)
- Grade 4 : mise en jeu du pronostic vital, intervention d'urgence
- Grade 5 : décès dû à l'évènement indésirable

L'investigateur doit notifier au promoteur, sans délai à compter du jour où il en a connaissance, tout évènement indésirable grave (EvIG), en utilisant la fiche de déclaration appropriée, s'il survient :
- à partir de la date de signature du consentement,
- pendant toute la durée de suivi du patient prévue par la recherche,
- Jusqu'à la fin du suivi du participant prévue par la recherche.
- Si l'investigateur prend connaissance d'un évènement indésirable grave, dont il suspecte un lien de causalité avec la recherche, survenant après la fin de l'essai clinique chez un participant qu'il a traité, il en informe le promoteur sans délai.

Le **formulaire de déclaration initiale d'EvIG doit être envoyé sans délai à compter du jour où l'investigateur en a connaissance** par fax ou email à la :

Vigilance des essais cliniques
Fax : 03 88 11 67 26
Email à : vigilance-ec@chru-strasbourg.fr

Tous les formulaires de SAE doivent être complétés, datés, signés par l'investigateur principal ou un investigateur autorisé.

Tous les EvIG doivent également être recueillis dans le CRF.

L'investigateur doit documenter au mieux l'évènement, en donner si possible, le diagnostic médical. L'investigateur doit s'assurer que les informations pertinentes de suivi soient communiquées au promoteur dès que possible.

L'investigateur doit transmettre, en plus du formulaire de notification des EvIG, les copies des résultats de laboratoire ou des comptes rendus d'examens ou d'hospitalisation renseignant l'évènement indésirable grave, y compris les résultats négatifs pertinents **sans omettre de rendre ces documents anonymes** et d'inscrire le code alpha numérique du patient dans l'étude.

L'investigateur doit suivre le patient ayant présenté un EvIG jusqu'à sa résolution, sa stabilisation à un niveau qu'il juge comme médicalement acceptable ou le retour à l'état antérieur, même si le patient a arrêté la procédure de recherche. Un complément d'information concernant l'évolution de l'évènement, si cette évolution n'est pas mentionnée dans le premier rapport, sera envoyé au promoteur par l'investigateur.

L'investigateur et le promoteur doivent évaluer, indépendamment l'un de l'autre, la causalité de l'évènement indésirable grave.

Tous les EvIG pour lesquels l'investigateur ou le promoteur estime qu'une relation de causalité peut-être raisonnablement envisagée sont considérés comme **des suspicions d'effets indésirables graves.**

Les EvIG seront également saisis dans le cahier d'observation électronique.

Certaines circonstances nécessitant une hospitalisation ne relèvent pas du critère de gravité : « hospitalisation/prolongation d'hospitalisation » comme :

- Admission pour raisons sociale ou administrative ;
- Hospitalisation prédéfinie par le protocole ;
- Hospitalisation pour traitement médical ou chirurgical programmé avant la recherche ;
- Hospitalisation pour la prise en charge d'une pathologie pré-existante (antécédent médical) non aggravée durant la participation à l'étude du sujet
- …

L'investigateur doit notifier sans délai tout fait nouveau dont il a connaissance au promoteur.

IX -3.3 Notification des grossesses

La survenue d'une grossesse durant la participation ou au décours immédiat d'une recherche, ne constitue pas un EvIG. Cependant, si une femme débute une grossesse dans le cadre de la recherche, la grossesse doit être notifiée suivant les mêmes modalités qu'un EvIG car elle fera l'objet d'un suivi particulier jusqu'à son issue.

Pour cela, l'investigateur informe sans délai la vigilance des essais cliniques du promoteur à l'aide du formulaire de déclaration d'une grossesse.

L'investigateur doit suivre la patiente jusqu'au terme de la grossesse ou de son interruption et en notifier l'issue au promoteur. Toute anomalie constatée sur le fœtus ou l'enfant doit être notifiée. Toute interruption volontaire de grossesse (IVG), interruption médicale de grossesse (IMG) ou fausse couche doit faire l'objet d'une notification de grossesse, et si un critère de gravité est présent, elle doit faire l'objet d'une notification d'EvIG. S'il s'agit d'une exposition paternelle, l'investigateur doit obtenir l'accord de la parturiente pour recueillir les informations sur la grossesse.

IX -3.4 Tableau récapitulatif du circuit des notifications

TYPE D'EVENEMENT	MODALITES DE NOTIFICATION	DELAI DE NOTIFICATION AU PROMOTEUR
**Evènement indésirable non grave**	Recueil dans le cahier d'observation	Pas de notification immédiate
**Evènement indésirable grave**	Formulaire de déclaration initiale d'EvIG + rapport de suivi si nécessaire + recueil dans le cahier d'observation	**Notification sans délai au promoteur** *(ou au plus tard dans les 24 heures)*
**Fait nouveau**	Formulaire de déclaration initiale d'EvIG	**Notification sans délai au promoteur**
**Grossesse**	Formulaire de déclaration grossesse	

IX -4. Déclaration par le promoteur des suspicions d'effets indésirables graves inattendus, des faits nouveaux et autres évènements.

Le promoteur évalue si l'effet indésirable grave est attendu ou inattendu en se basant sur la liste des évènements indésirables graves attendus décrits dans le protocole et sur les documents de référence.

Le promoteur déclare à l'ANSM et enregistre dans la base de données EudraVigilance tous les effets indésirables graves inattendus.

Dans le cas de cette recherche en insu, le promoteur déclare les effets indésirables graves inattendus à l'ANSM et Eudravigilance après avoir levé l'insu.

Tableau récapitulatif des déclarations EIGI

		**Délai de déclaration initiale**	**Déclaration aux autorités compétentes concernées**	**Délai du suivi**
RIPH1 médicament	EIGI Décès ou mise en jeu du pronostic vital	sans délai	ANSM<br>EMA<br>(Eudravigilance)	Max 8j
	Autres EIGI	Au plus tard 15j		

Le promoteur déclare **sans délai les faits nouveaux** survenus au cours de la recherche :
- à l'Autorité compétente
- au Comité de Protection des Personnes

Le promoteur et l'investigateur prennent les mesures urgentes appropriées. Le promoteur en informe l'Autorité Compétente et le Comité de Protection des Personnes. Le promoteur informe les investigateurs principaux.

IX -5. Rapport annuel de sécurité.

Le promoteur rédige un rapport annuel de sécurité à la date anniversaire de l'autorisation de la recherche (pour les études portant sur un médicament) / la première inclusion (pour tous les autres types de recherche), comprenant:

- la liste des effets indésirables graves susceptibles d'être liés aux traitements expérimentaux de la recherche incluant les effets graves attendus et inattendus, survenus dans l'essai concerné pendant la période couverte par le rapport,
- une analyse concise et critique de la sécurité des participants se prêtant à la recherche.
- les tableaux de synthèse de tous les évènements indésirables graves survenus dans l'essai concerné depuis le début de la recherche.

Ce rapport est envoyé à l'Autorité Compétente et au CPP dans les 60 jours suivant la date anniversaire de l'autorisation de la recherche/la première inclusion.

X - CREATION D'UN COMITE DE SURVEILLANCE INDEPENDANT

Dans la mesure où les risques liés à la chaque traitement sont identifiés, que toutes les précautions et mesures de surveillance ont été prises pour limiter ces risques, aucun comité de surveillance indépendant ne sera mis en place dans le cadre de cette étude.

XI- STATISTIQUES

XI-1. Description des Méthodes Statistiques Prévues

L'analyse statistique comportera une partie descriptive et une partie inférentielle. Les analyses se feront selon les méthodes bayésiennes.

L'analyse statistique descriptive des variables quantitatives se fera en donnant l'ensemble des valeurs observées (tri à plat des valeurs), avec la fréquence de chaque valeur, ainsi que sa fréquence relative. Ces fréquences seront données par valeur et sous forme cumulée. Pour chaque variable, seront donnés les paramètres de position (moyenne, médiane, minimum, maximum, premier et troisième quartiles) ainsi que les paramètres de dispersion (variance, écart-type, étendue, écart interquartile). Le caractère gaussien des données sera testé par le test de Shapiro-Wilk et par des diagrammes quantile-quantile.

Le descriptif des variables qualitatives sera fait en donnant les effectifs et proportions de chaque modalité dans l'échantillon. Chaque fois que cela sera utile, des tableaux croisés seront donnés avec effectifs, proportions par ligne, proportions par colonne et proportions par rapport au total, pour chaque case du tableau.

Pour répondre à l'objectif principal, l'analyse inférentielle permettra de comparer le rapport PaO_2 sur FiO_2 en fonction du groupe avec une régression linéaire simple ou généralisée en fonction de la distribution des données.

Pour répondre aux objectifs secondaires, des régressions linéaires bayésiennes seront réalisées pour analyser les variables quantitatives et des régressions logistiques bayésiennes pour analyser les variables qualitatives.

Des analyses en sous-groupe et avec la présence d'une interaction entre le groupe de traitement et la positivité au Sars-Cov-2 seront réalisées afin de déterminer l'effet de la positivité au Sars-Cov-2 associé à celui de la lidocaïne sur les critères de jugement principal et secondaires.

Les lois a priori seront d'une part très peu informatives et d'autre part informatives (en prenant les résultats de l'étude de Jabaudon (130) à propos de l'utilisation du sévoflurane pour sédation des patients atteints de SDRA) dans le cadre d'une analyse de sensibilité.

Pour chaque analyse, la loi a posteriori du paramètre d'intérêt (proportion, moyenne, coefficient de la régression, etc.) sera estimée en utilisant la méthode de Monte Carlo par chaînes de Markov. Le nombre d'itérations retenues par défaut sera de 100 000 après suppression des 10 000 premières et en retenant une valeur sur 2 (210 000 itérations seront donc réalisées). La convergence sera estimée graphiquement. L'autocorrélation sera estimée graphiquement et si nécessaire, le nombre d'itérations sera augmenté pour augmenter le pas des valeurs retenues dans le but de réduire autant que faire se peut l'autocorrélation.

Les analyses seront faites avec le logiciel R dans sa version la plus à jour au moment de l'analyse ainsi qu'avec tous les progiciels requis pour mener à bien les analyses et avec les logiciels OpenBUGS et JAGS.

XI-2. Nombre de Personnes à Inclure et Justification

D'après les données d'une étude concernant l'utilisation de sévoflurane dans le SDRA (130), le rapport PaO_2 sur FiO_2 est estimé à 205±56 dans le groupe avec sévoflurane et à 166±59 dans le groupe midazolam. Par conséquent, un effectif de 46 personnes par groupe permettra de montrer une différence de rapport PaO2 sur FiO2 avec une puissance de 90% et un risque de première espèce de 5%. Quatre patients sont ajoutés dans chaque groupe pour permettre de prévoir d'éventuelles sorties d'étude. Au total, 100 sujets seront donc inclus.

Il n'est pas possible de savoir la proportion de patients avec Covid-19, toutefois, cet effectif permettra d'obtenir au moins 50 sujets dans un sous-groupe (avec ou sans Covid-19) ce qui nous permettra d'obtenir une puissance d'au moins 65 % dans ce sous-groupe.

Le calcul a été réalisé avec le logiciel R 3.6.3.

XI-3- Niveau de significativité statistique

Il n'y a pas de niveau de significativité en bayésien toutefois les intervalles de crédibilité seront calculés à 95 % selon la méthode des quantiles. L'effet d'un facteur sera considéré comme présent si la probabilité que l'effet soit supérieur à la valeur de référence est supérieure à 0,975 ou inférieure à 0,025.

XI-4. Critères statistiques d'arrêt de l'étude

Les analyses étant réalisées en bayésien, il n'y a pas d'inflation du risque alpha. Des analyses seront réalisées hebdomadairement et l'étude sera arrêtée si la probabilité que l'effet du traitement sur le rapport PaO_2 sur FiO_2 est supérieur à 0,99 ou inférieur à 0,05.

XI-5. Modalités de prise en compte des données manquantes, non utilisées ou non valides

La proportion de données manquantes sera donnée pour chaque variable. Une description univariée et bivariée sera réalisée permettant d'estimer le mécanisme probable à l'origine des données manquantes (complètement aléatoire, aléatoire ou non aléatoire). Les données manquantes seront traitées par simple délétion des cas si ces valeurs sont très peu nombreuses (moins de 3 % pour une analyse donnée) ou par imputation multiple dans le cas contraire, jusqu'à une proportion maximum de données manquantes de l'ordre de 30%. Au-delà de cette proportion, la pertinence de chaque analyse sera discutée au cas par cas. L'imputation se fera directement dans les modèles.

Les données aberrantes seront discutées et une recherche sera faite pour tenter de les expliquer. En cas d'erreur matérielle, elle sera rectifiée. Sinon, une double analyse avec et sans la donnée concernée sera pratiquée et son inclusion ou sa non inclusion sera discutée au cas par cas en fonction de l'aberration.

XI-6. Gestion des modifications apportées au plan statistique initial

Les éventuelles modifications à apporter au plan d'analyse statistique seront proposées par le statisticien en charge de l'étude et validées par consensus entre le statisticien et l'investigateur principal.

XI-7. Choix des personnes à inclure dans les analyses

Les données de toutes les personnes incluses avec au moins une donnée non-manquante seront analysées.

XI-8. Remplacement des sujets sortis d'étude

Il n'y aura pas de remplacement des éventuels sujets sortis de l'étude, dix patients étant ajoutés pour permettre de prévoir d'éventuelles sorties d'étude.

XII- DROIT D'ACCES AUX DONNEES ET DOCUMENTS SOURCE

L'investigateur autorise l'accès direct aux données et documents source conformément aux dispositions législatives et réglementaires en vigueur, aux personnes chargées du contrôle de qualité de la recherche dûment mandatées à cet effet par le promoteur et à toutes personnes appelées à collaborer aux essais. Ces personnes prennent toutes les précautions nécessaires en vue d'assurer la confidentialité des informations relatives à l'essai, aux produits expérimentaux, aux personnes qui s'y prêtent et notamment en ce qui concerne leur identité ainsi qu'aux résultats obtenus. Les données collectées par ces personnes au cours des contrôles de qualité ou des audits sont alors rendues anonymes.
Les investigateurs acceptent de se conformer aux exigences du promoteur et de l'Autorité Compétente en ce qui concerne un audit ou une inspection de l'étude.
L'audit pourra s'appliquer à tous les stades de l'étude, du développement du protocole à la publication des résultats et au classement des données utilisées ou produites dans le cadre de l'étude.

XIII - RECUEIL DES DONNEES

XIII -1. Données sources

Les documents source sont définis comme tout document ou objet original permettant de prouver l'existence ou l'exactitude d'une donnée ou d'un fait enregistré au cours de l'étude (dossier médical du patient, feuille de résultat de laboratoire, correspondance médicale, données informatisées d'un appareil de mesure...etc.).

L'investigateur autorise l'accès direct à tous les documents source conformément aux dispositions législatives et réglementaires en vigueur, aux personnes chargées du contrôle de qualité de la recherche dûment mandatées à cet effet par le promoteur et à toutes personnes appelées à collaborer aux essais. Ces personnes prennent toutes les précautions nécessaires en vue d'assurer la confidentialité des informations relatives à l'essai et aux personnes qui s'y prêtent, notamment en ce qui concerne leur identité ainsi qu'aux résultats obtenus.

Les investigateurs acceptent de se conformer aux exigences du promoteur et de l'Autorité Compétente en ce qui concerne un audit ou une inspection de l'étude. L'audit pourra s'appliquer à tous les stades de l'étude, du développement du protocole à la publication des résultats et au classement des données utilisées ou produites dans le cadre de l'étude.

XIII -2. Cahier d'observation électronique (eCRF).

Toutes les données de cette étude seront anonymisées puis retranscrites dans un cahier d'observation électronique par l'investigateur ou une personne qu'il aura désignée.

Une trace de toutes les modifications apportées aux cahiers d'observations sera conservée. Ces traces doivent permettent de connaître, pour toute modification, la valeur antérieure, la date de la modification et la personne ayant procédé à la modification.

Pour attester l'authenticité et la précision des données figurant dans le cahier d'observation, l'investigateur apposera sa signature, électronique dans le cadre d'un eCRF, conformément aux ICH-GCP part 11.

Les données collectées dans l'e –CRF sont les suivantes :

<u>Visite d'inclusion</u>

Les antécédents :
1. Données démographiques (l'âge et le sexe)

2. les antécédents médicaux, l'examen clinique et les critères pronostiques négatifs : âge élevé, comorbidités, défaillance d'organe associée, SOFA>5, D- dimères > 1 µg/mL, le mode de vie du patient (autonomie ?) afin de définir le score de fragilité (score CFS)
3. Taille; poids réel, poids idéal théorique; indice de masse corporelle (IMC)
4. Temps passé sous respirateur avant l'inclusion
5. Type d'intubation, lieu où a eu lieu l'intubation (pré-hospitalier, SAU, USI, Bloc opératoire, réanimation…)
6. Service où a lieu l'inclusion
8. Facteurs de risque de SDRA autre que le Covid-19 (septicémie, inhalation, traumatisme, pneumonie, autres)
9. Le tabagisme
10. Les facteurs de risques de gravité d'une pneumopathie à COVID-19

Les paramètres de base (informations retrouvées dans le dossier au moment de l'inclusion) :
1. Antécédents et examen physique : Signes vitaux : fréquence cardiaque (battements/min), tension artérielle systolique et diastolique systémique (mmHg), température corporelle (°C)
2. Mode ventilatoire, FR réglée, FR réelle, ventilation minute, volume courant, FiO2, PEP, auto-PE, rapport I:E, plateau, pression maximale et moyenne des voies respiratoires
3. Administration des médicaments (posologie)
   a) Sédatif intraveineux
   b) Opioïdes intraveineux
   c) Curare
   d) Corticostéroïdes intraveineux ou oraux (exprimés en équivalents-méthylprednisolone)
   e) Antibiotiques
   f) Catécholamines
4. Présence et site présumé d'infection
5. Scores SAPS II et APACHE II
6. Score SOFA : pour évaluation des fonctions cardiovasculaires, rénales, respiratoires, hépatiques et hématologiques
7. Traitement anticoagulant en cours ou préalable
8. Radiographie pulmonaire utilisée pour diagnostiquer le SDRA (pourcentage d'invasion)
10. TDM initiale si présente (gravité)

Paramètres de l'étude : les conditions suivantes seront assurées avant les mesures : pas de d'aspiration endobronchique pendant 10 minutes ; pas de procédures invasives ni de changement de respirateur pendant 30 minutes. Toutes les pressions vasculaires seront mesurées avec pour zéro de référence l'oreillette gauche.

1. Paramètres respiratoires, y compris le volume courant, la fréquence respiratoire, le rapport I:E, EtCO2, PaCO2, le pH artériel, FiO2, PEP, l'auto-PEP, la compliance et la résistance pulmonaire, la pression de plateau, de crête, la pression moyenne des voies respiratoires
   Mesures de référence pour évaluer l'épargne en hypnotique, morphiniques et curares
   Dose de sédation dans le bras interventionnel et contrôle :
      a. molécules et doses administrées d'hypnotiques, morphiniques (exprimés en équivalent morphine)

b. Curare : dose totale de perfusion de cisatracurium

2. Résultats du bilan biologique J0

Visite à H4

Paramètres de l'étude : Les conditions suivantes seront assurées avant les mesures : pas de d'aspiration endobronchique pendant 10 minutes ; pas de procédures invasives ni de changement de respirateur pendant 30 minutes. Toutes les pressions vasculaires seront mesurées avec pour zéro de référence l'oreillette gauche.

1. Paramètres respiratoires, y compris le volume courant, la fréquence respiratoire, le rapport I:E, EtCO2, PaCO2, le pH artériel, FiO2, PEP, l'auto-PEP, la compliance et la résistance pulmonaire, la pression de plateau, de crête, la pression moyenne des voies respiratoires
2. Évaluation de l'épargne en hypnotique, morphiniques et curares
   Dose de sédation dans le bras interventionnel et contrôle :
   a. molécules et doses administrées d'hypnotiques, morphiniques (exprimés en équivalent morphine)
   b. Curare : dose totale de perfusion de cisatracurium
3. Dosage de la lidocaïne plasmatique (donnée masquée)

Les visites quotidiennes de J1 à J28 ou sortie de réanimation si elle a lieu avant J28

Les visites de suivi seront quotidiennes et récolteront :
Mesures de référence (quotidiennes)
Les paramètres suivants seront mesurés et enregistrés quotidiennement dans la matinée en utilisant les valeurs les plus proches. Les conditions suivantes seront assurées avant les mesures : pas de d'aspiration endobronchique pendant 10 minutes ; pas de procédures invasives ni de changement de respirateur pendant 30 minutes. Toutes les pressions vasculaires seront mesurées avec pour zéro de référence l'oreillette gauche.

1. En cas de ventilation mécanique : mode ventilatoire, FR demandée et FR réelle, ventilation minute, volume courant, FiO_2, PEP, autoPEP, compliance et résistance pulmonaire, rapport I :E, pression de plateau, de pic, pression moyenne des voies aériennes, débit de crête et réglage du temps inspiratoire
2. PaO2, PaCO2, pH artériel et SpO2
3. Dose de sédation:
   a. molécules et doses administrées d'hypnotiques, morphiniques (exprimés en équivalent morphine)
   b. Curare : nécessité d'un recours à la curarisation IVSE ou à l'utilisation de bolus supplémentaires de cisatracurium pendant les 24 dernières heures (Oui / Non)
4. Procédures de sauvetage utilisées
   a) Monoxyde d'azote inhalé
   b) Époprosténol sodique
   c) Ventilation à haute fréquence
   d) ECMO
5. Administration de Corticostéroïdes oraux ou intraveineux (en équivalents méthylprednisolone)

6. Infection documentée (prélèvements bactériologiques, viraux, mycologiques)
7. Mesures hémodynamiques : pression artérielle systolique, moyenne et diastolique, dose maximale de noradrénaline ou d'un autre vasopresseur, taux de lactate sérique, tachycardie supraventriculaire (TSV) ou fibrillation auriculaire nouvelle
8. Critères de KDIGO pour les lésions rénales aigues
9. au niveau digestif : transit, vomissements…
10. à la levée des sédations CAM-ICU
11. score SOFA
12. Résultats des bilans biologiques

La visite du jour d'extubation/déventilation et le lendemain

a. Recherche de la présente d'une toux à l'extubation ou à la déventilation et dans les 24h suivant l'extubation
b. Recherche d'un mal de gorge à l'extubation ou dans les 24h suivant l'extubation

Une attention particulière sera portée à J2, date de recueil du critère de jugement principal (Rapport PaO2/FiO2).

Résultats des dosages biologiques

Les résultats des dosages biologiques seront également enregistrés aux temps suivants :

Bilan **quotidien** :
Temps de céphaline activé (TCA) et son ratio TCAr, gazométrie du sang artériel (dont PaO2, Lactates et bicarbonates), plaquettes, Fibrinogène.

Bilan réalisé à **J0, J7, J14 et J21**
Tropo HS, albumine, cholestérol.

Bilan réalisé à **J0, J2, J7, J14 et J21**
Triglycéride, ferritine, PCT, formule leucocytaire, d-dimères, IL-6, CRP, LDH, Tests thromboélastométriques (TEG6S® et/ou Quantra®).

Dosage plasmatique de la lidocaïne **H4, J2, J7 et J14**

Appel téléphonique à J60

Statut vivant/décédé

Appel téléphonique à J90

Statut vivant/décédé, retour à domicile, au travail, hospitalisation, ré hospitalisation, échelle qualité de vie questionnaire SF36 modifié

XIII -3. Base de données.

L'hébergement de la base de données couplée à l'eCRF CLEANWEB est géré par la société TELEMEDICINE Technologies S.A.S disposant d'un ensemble de Datacentres virtuels dédiés. Cette société assure la confidentialité, la sécurité, et l'intégrité des données, selon un plan de sécurité prédéfini par le Promoteur et conforme aux recommandations internationales (ICH-GCP part 11).
La gestion des données de l'essai sera réalisée à l'aide de la solution logicielle CLEANWEB® commercialisée par TELEMEDICINE Technologies S.A.S.
La liste des personnes autorisées à accéder aux données est définie par le porteur de projet (investigateur coordonnateur/principal) et le Promoteur. Chaque personne se verra allouer des identifiants strictement personnels et confidentiels pour accéder aux masques de saisie CLEANWEB®.

XIV - ASSURANCE DE LA QUALITE

XIV -1. Contrôle de cohérence.

Des contrôles de cohérence des données recueillies seront réalisés informatiquement selon des règles prédéfinies entre le promoteur et l'investigateur décris dans le plan de monitoring. Des demandes d'information ou « queries » seront envoyées à l'investigateur pour corriger ou clarifier certaines données.
Toute modification de données sera tracée via un audit trail consultable avec le logiciel CLEANWEB®.

XIV -2. Monitoring.

Un(e) attaché(e) de recherche clinique (ARC), délégué par le promoteur réalisera un contrôle qualité suivant le plan de monitoring défini préalablement au démarrage de l'essai. Après concertation avec l'équipe d'investigation, sur accord de ces derniers, et dès lors que les conditions le permettent, le monitoring à distance sera privilégié. Ce monitoring ne devra pas générer de surcroît d'activité pour l'équipe d'investigation et aura pour objet :

- De contrôler le respect du protocole,
- De vérifier les consentements éclairés,
- De vérifier la notification des Evènements Indésirables Graves, et le recueil des événements indésirables
- De suivre la traçabilité du produit expérimental (en lien avec la pharmacie et les infirmières en charge de la préparation des traitements, dispensation, condition de stockage et comptabilité)

- D'assurer le contrôle de qualité : confronter les données du cahier d'observation avec les documents sources du sujet.

Dans le cas où le monitoring ne pourra pas être effectué à distance, des visites seront planifiées sur site dans le respect des mesures de sécurité du service.

XV- ARCHIVAGE

Conformément à l'article R1123-68 du code de la santé publique et aux Bonnes Pratiques Cliniques, les documents et données relatifs à la recherche seront conservés par le promoteur et l'investigateur pendant toute la durée de la recherche et pour une durée de 15 ans suivant la fin de la recherche (recherches portant sur des médicaments, des dispositifs médicaux ou des dispositifs médicaux de diagnostic in vitro ou recherches ne portant pas sur un produit mentionné à l'article L.5311-1 du code de la santé publique)

Dans le centre participant à la recherche, tous les documents relatifs à la recherche sont sous la responsabilité de l'investigateur pendant toute la durée réglementaire d'archivage :

- Le protocole et les modifications éventuelles au protocole
- Les cahiers d'observation (copies)
- Les dossiers source des participants ayant signé un consentement
- Tous les autres documents et courriers relatifs à la recherche
- L'exemplaire original des consentements éclairés signés des participants

Toutes les données, tous les documents et rapports pourront faire l'objet d'audit ou d'inspection. Ces documents doivent être conservés de manière à pouvoir être facilement mis à disposition des autorités compétentes et leur être accessible, sur demande.

Dans le cas où l'investigateur ne peut plus assurer la conservation des documents de l'étude (par ex : départ en retraite), il désignera l'investigateur à qui la responsabilité de la conservation des documents a été déléguée et en informera par courrier le promoteur.

XVI- CONSIDERATIONS ETHIQUES

La recherche sera menée en conformité avec les principes de la Déclaration d'Helsinki, les Bonnes Pratiques Cliniques, le protocole et la réglementation en vigueur.

XVI 1. Comité de Protection des Personnes et Autorité compétente

Conformément à l'article L1121-4 du code de la santé publique, le promoteur procédera avant toute mise en œuvre de la recherche, à une demande d'avis contraignant auprès du Comité de Protection des Personnes (CPP) et à une demande d'autorisation auprès de l'Autorité compétente (AC).

Conformément à l'article L1123-9 du code de la santé publique, toute modification substantielle fera l'objet d'une demande d'avis contraignant auprès du Comité de Protection des Personnes (CPP) et d'une demande d'autorisation auprès de l'Autorité compétente (AC).

Aucune sélection ou inclusion n'aura lieu avant :

- L'avis favorable du Comité de Protection des Personnes,
- L'autorisation de l'Autorité compétente,
- L'information du Directeur Général de l'établissement de santé dans lequel se déroulera la recherche en cas d'étude multicentrique
- La réunion de mise en place de l'étude par le promoteur.

Par ailleurs, la prise en charge des patients COVID-19 suivra les recommandations du HCSP, le dernier avis datant du 17 juin 2020 (128), cette prise en charge est susceptible d'évolution, notamment lorsque des essais randomisés en cours seront disponibles. En cas de recommandation d'un traitement spécifique à la COVID-19, compatible avec la lidocaïne IVSE, le protocole fera l'objet d'un amendement. En cas de traitement incompatible avec l'utilisation de la lidocaïne IVSE, les patients COVID-19 ne seront plus inclus dans l'étude, afin de bénéficier du « Standard of Care ».

XVI -2. Information et consentement du volontaire

<u>Situation d'urgence vitale</u>

L'inclusion du sujet est réalisée dans la situation d'urgence vitale suivante : patient déjà intubé et sédaté au moment où il remplit les conditions d'inclusion avec nécessité de démarrer le traitement dans les 24h. Dans ces circonstances, le consentement du sujet, de sa famille ou de la personne de confiance (défini à l'article L1111-6) ne pourra pas être recueilli préalablement à la recherche. L'intéressé(e), sa famille ou la personne de confiance seront informés dès que possible et leur consentement leur sera demandé pour la poursuite éventuelle de la recherche.

Les informations communiquées sont résumées dans un document écrit remis à l'intéressé(e), sa famille ou la personne de confiance. Une fois que cette information aura été donnée, que l'investigateur aura répondu à l'ensemble des interrogations de l'intéressé(e), de sa famille ou de la

personne de confiance, l'investigateur recueillera auprès de l'intéressé(e), de sa famille ou de la personne de confiance le consentement par écrit. L'intéressé(e) est libre de refuser de poursuivre sa participation à l'étude, et il peut retirer à tout moment son consentement et ce, qu'elle qu'en soit la raison, et sans encourir aucune responsabilité, ni aucun préjudice. De même la famille de l'intéressé(e), la personne de confiance peuvent retirer à tout moment leur consentement et ce, qu'elle qu'en soit la raison, et sans que l'intéressé(e) n'encoure aucune responsabilité, ni aucun préjudice.

Un exemplaire signé du consentement est remis au sujet, à sa famille, ou à la personne de confiance.

L'intéressé(e), la personne de confiance ou les membres de la famille, pourront également s'opposer à l'utilisation des données concernant le patient dans le cadre de la recherche.

<u>Majeurs sous curatelle</u>

Dans le cas où un patient serait sous curatelle (découverte après l'inclusion), conformément à l'article L1122-2 du code de la santé publique, une information adaptée à la capacité de compréhension du patient sous curatelle sera fournie par l'investigateur dès que celui-ci sera apte à comprendre les informations communiquées et à donner son consentement. Le consentement sera recueilli auprès du patient sous curatelle assisté par son curateur.

<u>Majeurs sous tutelle</u>

Dans le cas où un patient serait sous tutelle (découverte après l'inclusion), conformément à l'article L1122-2 du code de la santé publique, une information adaptée à la capacité de compréhension du patient sous tutelle sera fournie par l'investigateur dès que celui-ci sera apte. Le patient sous tutelle sera consulté dans la mesure où son état le permet. L'adhésion personnelle du patient sous tutelle en vue de sa participation à la recherche impliquant la personne humaine sera recherchée. Il ne pourra être passé outre à son refus ou à la révocation de son acceptation. L'autorisation de participation à la recherche sera donnée par le représentant légal du majeur sous tutelle.

<u>Patient décédé avant que l'information n'ait pu être délivrée à la personne de confiance / un membre de la famille</u>

Dans l'hypothèse où un patient décéderait avant que l'information ait pu être délivrée à la personne de confiance ou, à la famille ou aux proches, une note d'information spécifique sera remise à ces derniers ultérieurement afin de leur permettre de s'opposer à l'utilisation des données concernant le

patient dans le cadre de cette recherche, en application des dispositions de l'article L.1122-1-3 du code de la santé publique.

XVI -3. Constitution d'une collection d'échantillons biologiques

Dans le cadre de cette recherche, le Promoteur constitue une collection d'Echantillons biologiques humains associés à des données cliniques, selon les termes de l'article L 1243-3 du Code de la Santé Publique (CSP), ci-après dénommée « la Collection ».

A la fin de la Recherche, la Collection sera dûment déclarée conformément à la réglementation en vigueur, en indiquant la mention des lieux où sont conservés les échantillons, ci-après désignés « les Echantillons ». Le Promoteur est responsable scientifique de la Collection ainsi constituée.

Les prélèvements, recueils ou mises à disposition des Echantillons ne pourront être effectués qu'auprès des personnes y ayant dûment consenti, par le biais du formulaire de consentement spécifique à la Recherche.

En cas de retrait du consentement d'un patient au prélèvement et à l'utilisation de ses échantillons, le Promoteur devra en être informé sans délai et prendre les mesures adéquates concernant la destruction des Echantillons et données associées.

Toute nouvelle étude qui serait mise en œuvre à partir des échantillons et des données recueillies devra faire l'objet des formalités réglementaires adéquates auprès des autorités compétentes.

XVI -4. Protection des données à caractère personnel

Le traitement des données à caractère personnel mis en œuvre dans le cadre de la recherche sera réalisé dans les conditions définies par la loi n° 78-17 du 6 janvier 1978 modifiée relative à l'informatique, aux fichiers et aux libertés et des textes réglementaires pris pour son application et par le règlement UE 2016/679 du 27 avril 2016, relatif à la protection des personnes physiques à l'égard du traitement des données à caractère personnel.

Le traitement des données mis en œuvre dans le cadre de cette recherche est réalisé en conformité avec l'ensemble des dispositions de la méthodologie de référence MR001 actualisé au 03 mai 2018 par la délibération n°2018-153 à l'exception des modalités d'information des personnes concernées (inclusion de personnes se trouvant en situation d'urgence vitale immédiate). Les Hôpitaux

Universitaires de Strasbourg ont signé un engagement de conformité à la MR001 en date du 08 janvier 2009.

Dans le cas des patients inclus en situation d'urgence vitale pour lesquelles il n'aura pas été possible de recueillir leur consentement ou celui d'un membre de la famille/personne de confiance préalablement à leur inclusion, une d'autorisation de mise en œuvre du traitement de données a été délivrée par la CNIL le 10 novembre 2020 (Décision DR-2020-350).

XVI.5- Assurance

Les Hôpitaux Universitaires de Strasbourg, promoteurs de l'étude, ont souscrit pour toute la durée de l'étude une assurance garantissant sa propre responsabilité civile ainsi que celle de tout intervenant impliqué dans la réalisation de l'essai, indépendamment de la nature des liens existant entre les intervenants et le promoteur.

XVI -6. Financement

Cette recherche fait l'objet d'un financement par les Hôpitaux Universitaires de Strasbourg.

XVII - FIN DE RECHERCHE ET RAPPORT FINAL

La fin de la recherche correspond au terme de la participation du dernier sujet participant à la recherche.

Sur décision du CPP, du Promoteur ou de l'Investigateur, il est possible d'interrompre la recherche (en cas de difficultés de recrutement, ou de tout autre motif le justifiant).

Dans un délai de 10/11 mois suivant la fin de la recherche ou son interruption, un rapport final sera établi et signé par le promoteur et l'investigateur. Ce rapport sera tenu à la disposition de l'autorité compétente. Le promoteur transmettra au CPP et à l'ANSM les résultats de la recherche sous forme d'un résumé du rapport final dans un délai d'un an après la fin de la recherche.

XVIII - CONFIDENTIALITE ET PUBLICATION DES RESULTATS

L'investigateur principal doit s'assurer que l'anonymat des patients sera respecté. L'investigateur principal conserve une **liste confidentielle d'identification des patients inclus**.

Conformément à l'article R.5121-13 du Code de la Santé Publique, les investigateurs et toutes les personnes appelées à collaborer à l'essai sont tenus au secret professionnel en ce qui concerne notamment la nature des produits utilisés, l'essai, les personnes qui s'y prêtent et les résultats obtenus. Ils ne peuvent, sans l'accord du promoteur, donner d'informations relatives aux essais qu'au ministre chargé de la santé, aux médecins inspecteurs de santé publique, aux pharmaciens inspecteurs de santé publique, au directeur général et aux inspecteurs de l'Agence nationale de sécurité du médicament et des produits de santé.

Les essais ne peuvent faire l'objet d'aucun commentaire écrit ou oral sans l'accord conjoint de l'expérimentateur ou de l'investigateur et du promoteur. **Tout rapport, toute communication orale ou écrite occasionnés par cette étude devront être transmis au promoteur.**

XIX- BIBLIOGRAPHIE

1. Huang C, Wang Y, Li X, Ren L, Zhao J, Hu Y, et al. Clinical features of patients infected with 2019 novel coronavirus in Wuhan, China. Lancet Lond Engl. 15 2020;395(10223):497-506.

2. Nicastri E, D'Abramo A, Faggioni G, De Santis R, Mariano A, Lepore L, et al. Coronavirus disease (COVID-19) in a paucisymptomatic patient: epidemiological and clinical challenge in settings with limited community transmission, Italy, February 2020. Euro Surveill Bull Eur Sur Mal Transm Eur Commun Dis Bull. 2020;25(11).

3. Bouadma L, Lescure F-X, Lucet J-C, Yazdanpanah Y, Timsit J-F. Severe SARS-CoV-2 infections: practical considerations and management strategy for intensivists. Intensive Care Med. 26 févr 2020;1-4.

4. Bernard Stoecklin S, Rolland P, Silue Y, Mailles A, Campese C, Simondon A, et al. First cases of coronavirus disease 2019 (COVID-19) in France: surveillance, investigations and control measures, January 2020. Euro Surveill Bull Eur Sur Mal Transm Eur Commun Dis Bull. 2020;25(6).

5. WHO Director-General's opening remarks at the media briefing on COVID-19 - 11 March 2020 [Internet]. [cité 16 avr 2020]. Disponible sur: https://www.who.int/dg/speeches/detail/who-director-general-s-opening-remarks-at-the-media-briefing-on-covid-19---11-march-2020

6. Zhou F, Yu T, Du R, Fan G, Liu Y, Liu Z, et al. Clinical course and risk factors for mortality of adult inpatients with COVID-19 in Wuhan, China: a retrospective cohort study. The Lancet. mars 2020;395(10229):1054-62.

7. Gattinoni L, Chiumello D, Caironi P, Busana M, Romitti F, Brazzi L, et al. COVID-19 pneumonia: different respiratory treatments for different phenotypes? Intensive Care Med. 14 avr 2020;1-4.

8. The Lille COVID-19 ICU and Anatomopathology Group, Copin M-C, Parmentier E, Duburcq T, Poissy J, Mathieu D. Time to consider histologic pattern of lung injury to treat critically ill patients with COVID-19 infection. Intensive Care Med [Internet]. 23 avr 2020 [cité 26 avr 2020]; Disponible sur: http://link.springer.com/10.1007/s00134-020-06057-8

9. Leisman DE, Deutschman CS, Legrand M. Facing COVID-19 in the ICU: vascular dysfunction, thrombosis, and dysregulated inflammation. Intensive Care Med. 28 avr 2020;

10. Phua J, Weng L, Ling L, Egi M, Lim C-M, Divatia JV, et al. Intensive care management of coronavirus disease 2019 (COVID-19): challenges and recommendations. Lancet Respir Med. avr 2020;S2213260020301612.

11. ARDS Definition Task Force, Ranieri VM, Rubenfeld GD, Thompson BT, Ferguson ND, Caldwell E, et al. Acute respiratory distress syndrome: the Berlin Definition. JAMA. 20 juin 2012;307(23):2526-33.

12. Pugin J, Verghese G, Widmer MC, Matthay MA. The alveolar space is the site of intense

inflammatory and profibrotic reactions in the early phase of acute respiratory distress syndrome. Crit Care Med. févr 1999;27(2):304-12.

13. Park WY, Goodman RB, Steinberg KP, Ruzinski JT, Radella F, Park DR, et al. Cytokine balance in the lungs of patients with acute respiratory distress syndrome. Am J Respir Crit Care Med. 15 nov 2001;164(10 Pt 1):1896-903.

14. Xiong Y, Liu Y, Cao L, Wang D, Guo M, Jiang A, et al. Transcriptomic characteristics of bronchoalveolar lavage fluid and peripheral blood mononuclear cells in COVID-19 patients. Emerg Microbes Infect. 1 janv 2020;9(1):761-70.

15. Bellani G, Laffey JG, Pham T, Fan E, Brochard L, Esteban A, et al. Epidemiology, Patterns of Care, and Mortality for Patients With Acute Respiratory Distress Syndrome in Intensive Care Units in 50 Countries. JAMA. 23 févr 2016;315(8):788-800.

16. Chen G, Wu D, Guo W, Cao Y, Huang D, Wang H, et al. Clinical and immunologic features in severe and moderate Coronavirus Disease 2019. J Clin Invest. 27 mars 2020;

17. Mehta P, McAuley DF, Brown M, Sanchez E, Tattersall RS, Manson JJ. COVID-19: consider cytokine storm syndromes and immunosuppression. The Lancet [Internet]. 16 mars 2020 [cité 22 mars 2020];0(0). Disponible sur: https://www.thelancet.com/journals/lancet/article/PIIS0140-6736(20)30628-0/abstract

18. Leonard-Lorant I, Delabranche X, Severac F, Helms J, Pauzet C, Collange O, et al. Acute Pulmonary Embolism in COVID-19 Patients on CT Angiography and Relationship to D-Dimer Levels. Radiology. 23 avr 2020;201561.

19. Klok FA, Kruip MJHA, van der Meer NJM, Arbous MS, Gommers D a. MPJ, Kant KM, et al. Incidence of thrombotic complications in critically ill ICU patients with COVID-19. Thromb Res. 10 avr 2020;

20. Helms J, Kremer S, Merdji H, Clere-Jehl R, Schenck M, Kummerlen C, et al. Neurologic Features in Severe SARS-CoV-2 Infection. N Engl J Med. 15 avr 2020;0(0):null.

21. Richardson S, Hirsch JS, Narasimhan M, Crawford JM, McGinn T, Davidson KW, et al. Presenting Characteristics, Comorbidities, and Outcomes Among 5700 Patients Hospitalized With COVID-19 in the New York City Area. JAMA [Internet]. 22 avr 2020 [cité 26 avr 2020]; Disponible sur: https://jamanetwork.com/journals/jama/fullarticle/2765184

22. admin_sfar. Préconisations pour l'utilisation parcimonieuse des molécules en tension durant la pandémie COVID-19 [Internet]. Société Française d'Anesthésie et de Réanimation. 2020 [cité 17 avr 2020]. Disponible sur: https://sfar.org/preconisations-pour-lutilisation-parcimonieuse-des-molecules-en-tension-durant-la-pandemie-covid-19/

23. Lefrant J-Y, Fischer M-O, Potier H, Degryse C, Jaber S, Muller L, et al. A national healthcare response to intensive care bed requirements during the COVID-19 outbreak in France. Anaesth Crit Care Pain Med. 5 oct 2020;

24. Gauss T, Pasquier P, Joannes-Boyau O, Constantin J-M, Langeron O, Bouzat P, et al.

Preliminary pragmatic lessons from the SARS-CoV-2 pandemic in France. Anaesth Crit Care Pain Med. 2020;39(3):329-32.

25. Frank AJ, Thompson BT. Pharmacological treatments for acute respiratory distress syndrome. Curr Opin Crit Care. févr 2010;16(1):62-8.

26. admin_sfar. Recommandations pour la prise en charge du SDRA [Internet]. Société Française d'Anesthésie et de Réanimation. 2018 [cité 7 juin 2020]. Disponible sur: https://sfar.org/recommandations-prise-charge-sdra/

27. RECOVERY Collaborative Group, Horby P, Lim WS, Emberson JR, Mafham M, Bell JL, et al. Dexamethasone in Hospitalized Patients with Covid-19 - Preliminary Report. N Engl J Med. 17 juill 2020;

28. WHO Rapid Evidence Appraisal for COVID-19 Therapies (REACT) Working Group, Sterne JAC, Murthy S, Diaz JV, Slutsky AS, Villar J, et al. Association Between Administration of Systemic Corticosteroids and Mortality Among Critically Ill Patients With COVID-19: A Meta-analysis. JAMA. 06 2020;324(13):1330-41.

29. Infectiologie P le 09/11/2020 dans. Recommandations actualisées de la prise en charge des patients COVIDs [Internet]. SRLF. 2020 [cité 10 nov 2020]. Disponible sur: https://www.srlf.org/recommandations-actualisees-de-la-prise-en-charge-des-patients-covids/

30. Utilisation de la dexaméthasone et d'autres corticoïdes dans le Covid-19 [Internet]. [cité 9 nov 2020]. Disponible sur: https://www.hcsp.fr/Explore.cgi/AvisRapportsDomaine?clefr=935

31. Alhazzani W, Møller MH, Arabi YM, Loeb M, Gong MN, Fan E, et al. Surviving Sepsis Campaign: guidelines on the management of critically ill adults with Coronavirus Disease 2019 (COVID-19). Intensive Care Med. 28 mars 2020;1-34.

32. Krishnamoorthy V, Chung L. Bench-to-bedside: The use of local anesthetics to attenuate inflammation in acute respiratory distress syndrome. Int J Crit Illn Inj Sci. 2014;4(2):98-100.

33. Beaussier M. La lidocaïne intraveineuse. Prat En Anesth Réanimation. févr 2020;24(1):5-9.

34. Beaussier M, Delbos A, Maurice-Szamburski A, Ecoffey C, Mercadal L. Perioperative Use of Intravenous Lidocaine. Drugs. août 2018;78(12):1229-46.

35. Downes H, Loehning RW. Local anesthetic contracture and relaxation of airway smooth muscle. Anesthesiology. nov 1977;47(5):430-6.

36. Gaughen CM, Durieux M. The effect of too much intravenous lidocaine on bispectral index. Anesth Analg. déc 2006;103(6):1464-5.

37. Gottschalk A, McKay AM, Malik ZM, Forbes M, Durieux ME, Groves DS. Systemic lidocaine decreases the Bispectral Index in the presence of midazolam, but not its absence. J Clin Anesth. mars 2012;24(2):121-5.

38. Hans GA, Lauwick SM, Kaba A, Bonhomme V, Struys MMRF, Hans PC, et al. Intravenous lidocaine infusion reduces bispectral index-guided requirements of propofol only during surgical stimulation. Br J Anaesth. oct 2010;105(4):471-9.

39. Harvey KP, Adair JD, Isho M, Robinson R. Can intravenous lidocaine decrease postsurgical ileus and shorten hospital stay in elective bowel surgery? A pilot study and literature review. Am J Surg. août 2009;198(2):231-6.

40. Cooke C, Kennedy ED, Foo I, Nimmo S, Speake D, Paterson HM, et al. Meta-analysis of the effect of perioperative intravenous lidocaine on return of gastrointestinal function after colorectal surgery. Tech Coloproctology. 2019;23(1):15-24.

41. Tay MZ, Poh CM, Rénia L, MacAry PA, Ng LFP. The trinity of COVID-19: immunity, inflammation and intervention. Nat Rev Immunol. 2020;20(6):363-74.

42. Ortiz MP, Godoy MC de M, Schlosser RS, Ortiz RP, Godoy JPM, Santiago ES, et al. Effect of endovenous lidocaine on analgesia and serum cytokines: double-blinded and randomized trial. J Clin Anesth. déc 2016;35:70-7.

43. Luostarinen V, Evers H, Lyytikäinen MT, Scheinin null, Wahlén A. Antithrombotic effects of lidocaine and related compounds on laser-induced microvascular injury. Acta Anaesthesiol Scand. févr 1981;25(1):9-11.

44. Tobias MD, Henry C, Augostides YGT. Lidocaine and bupivacaine exert differential effects on whole blood coagulation. J Clin Anesth. févr 1999;11(1):52-5.

45. Nishino T, Hiraga K, Sugimori K. Effects of i.v. lignocaine on airway reflexes elicited by irritation of the tracheal mucosa in humans anaesthetized with enflurane. Br J Anaesth. juin 1990;64(6):682-7.

46. Pandey CK, Raza M, Ranjan R, Singhal V, Kumar M, Lakra A, et al. Intravenous lidocaine 0.5 mg.kg-1 effectively suppresses fentanyl-induced cough. Can J Anaesth J Can Anesth. févr 2005;52(2):172-5.

47. Clivio S, Putzu A, Tramèr MR. Intravenous Lidocaine for the Prevention of Cough: Systematic Review and Meta-analysis of Randomized Controlled Trials. Anesth Analg. 2019;129(5):1249-55.

48. Aminnejad R, Salimi A, Saeidi M. Lidocaine during intubation and extubation in patients with coronavirus disease (COVID-19). Can J Anaesth. 16 mars 2020;1.

49. Klinger RY, Cooter M, Berger M, Podgoreanu MV, Stafford-Smith M, Ortel TL, et al. Effect of intravenous lidocaine on the transcerebral inflammatory response during cardiac surgery: a randomized-controlled trial. Can J Anaesth J Can Anesth. nov 2016;63(11):1223-32.

50. Butterworth JF 4th, Strichartz GR. Molecular mechanisms of local anesthesia: a review. Anesthesiology. avr 1990;72(4):711-34.

51. BREMER G, EKMANNER S. Xylocaine; a new local anaesthetic. Br Dent J. 17 déc 1948;85(12):278-81.

52. Rosen MR, Hoffman BF. Mechanisms of action of antiarrhythmic drugs. Circ Res. janv 1973;32(1):1-8.

53. Choquet O, Zetlaoui PJ. Techniques d'anesthésie locorégionale du membre inférieur.

Httpwwwem-Premiumcomdatatraitesan36-30022 [Internet]. [cité 18 févr 2014]; Disponible sur: http://www.em-premium.com/article/27773

54. Zetlaoui P-J, Choquet O. Techniques d'anesthésie locorégionale du membre supérieur. EMC - Anesth-Réanimation. janv 2013;10(1):1-28.

55. Verzilli D, Viel E, Chanques G, Beaussier M, Delay J-M, Jaber S, et al. Anesthésie et analgésie péridurales (obstétrique exclue). EMC - Anesth-Réanimation. janv 2009;6(4):1-24.

56. Viel E, Gentili M, Ripart J, Eledjam J-J. Rachianesthésie chez l'adulte (obstétrique et pédiatrie exclues). EMC - Anesth-Réanimation. janv 2010;7(2):1-17.

57. Lev R, Rosen P. Prophylactic lidocaine use preintubation: A review. J Emerg Med. juill 1994;12(4):499-506.

58. Marret E, Rolin M, Beaussier M, Bonnet F. Meta-analysis of intravenous lidocaine and postoperative recovery after abdominal surgery. Br J Surg. 2008;95(11):1331–1338.

59. Marret E, Ynineb Y, Tounou-Akue F, Ott M. Place de la lidocaïne par voie intraveineuse dans la prise en charge de la douleur postopératoire. Prat En Anesth Réanimation. avr 2011;15(2):104-9.

60. Attal N, Rouaud J, Brasseur L, Chauvin M, Bouhassira D. Systemic lidocaine in pain due to peripheral nerve injury and predictors of response. Neurology. 27 janv 2004;62(2):218-25.

61. Tremont-Lukats IW, Challapalli V, McNicol EDRp, Lau J, Carr DB. Systemic Administration of Local Anesthetics to Relieve Neuropathic Pain: A Systematic Review and Meta-Analysis. Anesth Analg Dec 2005. 2005;101(6):1738-49.

62. Waller ES. Pharmacokinetic principles of lidocaine dosing in relation to disease state. J Clin Pharmacol. avr 1981;21(4):181-94.

63. Rowland M, Thomson PD, Guichard A, Melmon KL. Disposition kinetics of lidocaine in normal subjects. Ann N Y Acad Sci. 6 juill 1971;179:383-98.

64. Thomson PD, Melmon KL, Richardson JA, Cohn K, Steinbrunn W, Cudihee R, et al. Lidocaine pharmacokinetics in advanced heart failure, liver disease, and renal failure in humans. Ann Intern Med. avr 1973;78(4):499-508.

65. Demartin S, Orlando R, Bertoli M, Pegoraro P, Palatini P. Differential effect of chronic renal failure on the pharmacokinetics of lidocaine in patients receiving and not receiving hemodialysis. Clin Pharmacol Ther. déc 2006;80(6):597-606.

66. Wood JN, Boorman JP, Okuse K, Baker MD. Voltage-gated sodium channels and pain pathways. J Neurobiol. oct 2004;61(1):55-71.

67. Kalso E. Sodium channel blockers in neuropathic pain. Curr Pharm Des. 2005;11(23):3005-11.

68. Osawa Y, Oda A, Iida H, Tanahashi S, Dohi S. The effects of class Ic antiarrhythmics on tetrodotoxin-resistant Na+ currents in rat sensory neurons. Anesth Analg. août 2004;99(2):464-71, table of contents.

69. Hollmann MW, Ritter CH, Henle P, de Klaver M, Kamatchi GL, Durieux ME. Inhibition of

m3 muscarinic acetylcholine receptors by local anaesthetics. Br J Pharmacol. mai 2001;133(1):207-16.

70. Biella G, Sotgiu ML. Central effects of systemic lidocaine mediated by glycine spinal receptors: an iontophoretic study in the rat spinal cord. Brain Res. 19 févr 1993;603(2):201-6.

71. Cohen SP, Mao J. Is the analgesic effect of systemic lidocaine mediated through opioid receptors? Acta Anaesthesiol Scand. août 2003;47(7):910-1.

72. Nagy I, Woolf CJ. Lignocaine selectively reduces C fibre-evoked neuronal activity in rat spinal cord in vitro by decreasing N-methyl-d-aspartate and neurokinin receptor-mediated post-synaptic depolarizations; implications for the development of novel centrally acting analgesics. Pain. janv 1996;64(1):59-70.

73. Rooney BA, Crown ED, Hulsebosch CE, McAdoo DJ. Preemptive analgesia with lidocaine prevents Failed Back Surgery Syndrome. Exp Neurol. avr 2007;204(2):589-96.

74. Lin S-C, Yeh J-H, Chen C-L, Chou S-H, Tsai Y-J. Effects of local lidocaine treatment before and after median nerve injury on mechanical hypersensitivity and microglia activation in rat cuneate nucleus. Eur J Pain Lond Engl. avr 2011;15(4):359-67.

75. Slooter AJC, Van De Leur RR, Zaal IJ. Delirium in critically ill patients. Handb Clin Neurol. 2017;141:449-66.

76. Kotfis K, Williams Roberson S, Wilson JE, Dabrowski W, Pun BT, Ely EW. COVID-19: ICU delirium management during SARS-CoV-2 pandemic. Crit Care [Internet]. 28 avr 2020 [cité 3 mai 2020];24. Disponible sur: https://www.ncbi.nlm.nih.gov/pmc/articles/PMC7186945/

77. Fernandes Fraceto L, Spisni A, Schreier S, de Paula E. Differential effects of uncharged aminoamide local anesthetics on phospholipid bilayers, as monitored by 1H-NMR measurements. Biophys Chem. 1 mai 2005;115(1):11-8.

78. Kitagawa N, Oda M, Totoki T. Possible mechanism of irreversible nerve injury caused by local anesthetics: detergent properties of local anesthetics and membrane disruption. Anesthesiology. avr 2004;100(4):962-7.

79. Aydin ON, Eyigor M, Aydin N. Antimicrobial activity of ropivacaine and other local anaesthetics. Eur J Anaesthesiol. oct 2001;18(10):687-94.

80. Chandan SS, Faoagali J, Wainwright CE. Sensitivity of respiratory bacteria to lignocaine. Pathology (Phila). août 2005;37(4):305-7.

81. Goehring LS, Brandes K, Ashton LV, Wittenburg LA, Olea-Popelka FJ, Lunn DP, et al. Anti-inflammatory drugs decrease infection of brain endothelial cells with EHV-1 in vitro. Equine Vet J. 2017;49(5):629-36.

82. K Y, S H. Destabilization of Herpes Simplex Virus Type 1 Virions by Local Anesthetics, Alkaline pH, and Calcium Depletion [Internet]. Vol. 108, Archives of virology. Arch Virol; 1989 [cité 20 mai 2020]. Disponible sur: https://pubmed.ncbi.nlm.nih.gov/2556981/?from_term=lidocaine+virus&from_page=5&from_pos=8

83. Kranke P, Jokinen J, Pace NL, Schnabel A, Hollmann MW, Hahnenkamp K, et al. Continuous

intravenous perioperative lidocaine infusion for postoperative pain and recovery. In: Cochrane Database of Systematic Reviews [Internet]. John Wiley & Sons, Ltd; 2015 [cité 13 oct 2015]. Disponible sur: http://onlinelibrary.wiley.com.gate2.inist.fr/doi/10.1002/14651858.CD009642.pub2/abstract

84. Weibel S, Jokinen J, Pace NL, Schnabel A, Hollmann MW, Hahnenkamp K, et al. Efficacy and safety of intravenous lidocaine for postoperative analgesia and recovery after surgery: a systematic review with trial sequential analysis. Br J Anaesth. juin 2016;116(6):770-83.

85. Weibel S, Jelting Y, Pace NL, Helf A, Eberhart LH, Hahnenkamp K, et al. Continuous intravenous perioperative lidocaine infusion for postoperative pain and recovery in adults. Cochrane Database Syst Rev. 04 2018;6:CD009642.

86. Devlin JW, Skrobik Y, Gélinas C, Needham DM, Slooter AJC, Pandharipande PP, et al. Clinical Practice Guidelines for the Prevention and Management of Pain, Agitation/Sedation, Delirium, Immobility, and Sleep Disruption in Adult Patients in the ICU. Read Online Crit Care Med Soc Crit Care Med. sept 2018;46(9):e825.

87. Mo Y, Thomas MC, Antigua AD, Ebied AM, Karras GE. Continuous Lidocaine Infusion as Adjunctive Analgesia in Intensive Care Unit Patients. J Clin Pharmacol. 2017;57(7):830-6.

88. Forster C, Vanhaudenhuyse A, Gast P, Louis E, Hick G, Brichant J-F, et al. Intravenous infusion of lidocaine significantly reduces propofol dose for colonoscopy: a randomised placebo-controlled study. Br J Anaesth. nov 2018;121(5):1059-64.

89. Rappels sur le Syndrome de perfusion du propofol - Point d’Information - ANSM : Agence nationale de sécurité du médicament et des produits de santé [Internet]. [cité 7 juin 2020]. Disponible sur: https://www.ansm.sante.fr/S-informer/Points-d-information-Points-d-information/Rappels-sur-le-Syndrome-de-perfusion-du-propofol-Point-d-Information

90. de Klaver MJM, Buckingham M-G, Rich GF. Lidocaine attenuates cytokine-induced cell injury in endothelial and vascular smooth muscle cells. Anesth Analg. août 2003;97(2):465-70, table of contents.

91. Hollmann MW, Gross A, Jelacin N, Durieux ME. Local anesthetic effects on priming and activation of human neutrophils. Anesthesiology. juill 2001;95(1):113-22.

92. Hollmann MW, Durieux ME. Local anesthetics and the inflammatory response: a new therapeutic indication? Anesthesiology. sept 2000;93(3):858-75.

93. Cassuto J, Sinclair R, Bonderovic M. Anti-inflammatory properties of local anesthetics and their present and potential clinical implications. Acta Anaesthesiol Scand. mars 2006;50(3):265-82.

94. van der Wal SEI, van den Heuvel S a. S, Radema SA, van Berkum BFM, Vaneker M, Steegers M a. H, et al. The in vitro mechanisms and in vivo efficacy of intravenous lidocaine on the neuroinflammatory response in acute and chronic pain. Eur J Pain Lond Engl. mai 2016;20(5):655-74.

95. Wickström K, Stavréus-Evers A, Vercauteren O, Olovsson M, Edelstam G. Effect of Lignocaine on IL-6, IL-8, and MCP-1 in Peritoneal Macrophages and Endometriotic Stromal Cells.

Reprod Sci Thousand Oaks Calif. 2017;24(3):382-92.

96. Yardeni IZ, Beilin B, Mayburd E, Levinson Y, Bessler H. The Effect of Perioperative Intravenous Lidocaine on Postoperative Pain and Immune Function. Anesth Analg Novemb 2009. 2009;109(5):1464-9.

97. Wang H-L, Yan H-D, Liu Y-Y, Sun B-Z, Huang R, Wang X-S, et al. Intraoperative intravenous lidocaine exerts a protective effect on cell-mediated immunity in patients undergoing radical hysterectomy. Mol Med Rep. nov 2015;12(5):7039-44.

98. Blumenthal S, Borgeat A, Pasch T, Reyes L, Booy C, Lambert M, et al. Ropivacaine decreases inflammation in experimental endotoxin-induced lung injury. Anesthesiology. mai 2006;104(5):961-9.

99. Piegeler T, Dull RO, Hu G, Castellon M, Chignalia AZ, Koshy RG, et al. Ropivacaine attenuates endotoxin plus hyperinflation-mediated acute lung injury via inhibition of early-onset Src-dependent signaling. BMC Anesthesiol. 2014;14:57.

100. Piegeler T, Votta-Velis EG, Bakhshi FR, Mao M, Carnegie G, Bonini MG, et al. Endothelial barrier protection by local anesthetics: ropivacaine and lidocaine block tumor necrosis factor-α-induced endothelial cell Src activation. Anesthesiology. juin 2014;120(6):1414-28.

101. Mikawa K, Maekawa N, Nishina K, Takao Y, Yaku H, Obara H. Effect of lidocaine pretreatment on endotoxin-induced lung injury in rabbits. Anesthesiology. sept 1994;81(3):689-99.

102. Kiyonari Y, Nishina K, Mikawa K, Maekawa N, Obara H. Lidocaine attenuates acute lung injury induced by a combination of phospholipase A2 and trypsin. Crit Care Med. févr 2000;28(2):484-9.

103. Takao Y, Mikawa K, Nishina K, Maekawa N, Obara H. Lidocaine attenuates hyperoxic lung injury in rabbits. Acta Anaesthesiol Scand. mars 1996;40(3):318-25.

104. Nishina K, Mikawa K, Takao Y, Shiga M, Maekawa N, Obara H. Intravenous lidocaine attenuates acute lung injury induced by hydrochloric acid aspiration in rabbits. Anesthesiology. mai 1998;88(5):1300-9.

105. Huang TK, Uyehara CFT, Balaraman V, Miyasato CY, Person D, Egan E, et al. Surfactant Lavage with Lidocaine Improves Pulmonary Function in Piglets after HCl-Induced Acute Lung Injury. Lung. 2004;182(1):15-25.

106. Flondor M, Listle H, Kemming GI, Zwissler B, Hofstetter C. Effect of inhaled and intravenous lidocaine on inflammatory reaction in endotoxaemic rats. Eur J Anaesthesiol. janv 2010;27(1):53-60.

107. Chen L-J, Ding Y-B, Ma P-L, Jiang S-H, Li K-Z, Li A-Z, et al. The protective effect of lidocaine on lipopolysaccharide-induced acute lung injury in rats through NF-κB and p38 MAPK signaling pathway and excessive inflammatory responses. Eur Rev Med Pharmacol Sci. 2018;22(7):2099-108.

108. Lin S, Jin P, Shao C, Lu W, Xiang Q, Jiang Z, et al. Lidocaine attenuates lipopolysaccharide-induced inflammatory responses and protects against endotoxemia in mice by suppressing HIF1α-induced glycolysis. Int Immunopharmacol. mars 2020;80:106150.

109. Gilbert CRA, Hanson IR, Brown AB, Hingson RA. Intravenous use of xylocaine. Curr Res Anesth Analg. déc 1951;30(6):301-13.

110. Hsu Y-W, Somma J, Newman MF, Mathew JP. Population pharmacokinetics of lidocaine administered during and after cardiac surgery. J Cardiothorac Vasc Anesth. déc 2011;25(6):931-6.

111. Meng L, Qiu H, Wan L, Ai Y, Xue Z, Guo Q, et al. Intubation and Ventilation amid the COVID-19 OutbreakWuhan's Experience. Anesthesiol J Am Soc Anesthesiol [Internet]. 18 mars 2020 [cité 27 avr 2020]; Disponible sur: https://anesthesiology.pubs.asahq.org/article.aspx?articleid=2763453

112. Goodman NW, Stratford N. Effect of i.v. lignocaine on the breathing of patients anaesthetized with propofol. Br J Anaesth. nov 1995;75(5):573-7.

113. Groeben H, Silvanus MT, Beste M, Peters J. Combined intravenous lidocaine and inhaled salbutamol protect against bronchial hyperreactivity more effectively than lidocaine or salbutamol alone. Anesthesiology. oct 1998;89(4):862-8.

114. Groeben H. Effects of high thoracic epidural anesthesia and local anesthetics on bronchial hyperreactivity. J Clin Monit Comput. 2000;16(5-6):457-63.

115. Weiss EB, Hargraves WA, Viswanath SG. The inhibitory action of lidocaine in anaphylaxis. Am Rev Respir Dis. mai 1978;117(5):859-69.

116. Mihara T, Uchimoto K, Morita S, Goto T. The efficacy of lidocaine to prevent laryngospasm in children: a systematic review and meta-analysis. Anaesthesia. déc 2014;69(12):1388-96.

117. Bouaziz H, Aubrun F, Belbachir AA, Cuvillon P, Eisenberg E, Jochum D, et al. Échographie en anesthésie locorégionale. Ann Fr Anesth Réanimation. sept 2011;30(9):e33-5.

118. Colin C. Les blocs périphériques des membres chez l'adulte. Ann Fr Anesth Réanimation. juin 2003;22(6):567-81.

119. Di Gregorio G, Neal JM, Rosenquist RW, Weinberg GL. Clinical Presentation of Local Anesthetic Systemic Toxicity: A Review of Published Cases, 1979 to 2009. [Miscellaneous Article]. Reg Anesth Pain Med MarchApril 2010. 2010;35(2):181-7.

120. Dillane D, Finucane BT. Local anesthetic systemic toxicity. Can J Anaesth J Can Anesth. avr 2010;57(4):368-80.

121. Weinberg GL. Lipid Emulsion Infusion: Resuscitation for Local Anesthetic and Other Drug Overdose. Anesthesiology. juill 2012;117(1):180-7.

122. admin_sfar. Aides cognitives en anesthésie réanimation [Internet]. Société Française d'Anesthésie et de Réanimation. [cité 24 avr 2020]. Disponible sur: https://sfar.org/espace-professionel-anesthesiste-reanimateur/outils-professionnels/boite-a-outils/aides-cognitives-en-anesthesie-reanimation/

123. Cao B, Wang Y, Wen D, Liu W, Wang J, Fan G, et al. A Trial of Lopinavir–Ritonavir in Adults Hospitalized with Severe Covid-19. N Engl J Med. 18 mars 2020;0(0):null.

124. Ferrer M, Torres A. Epidemiology of ICU-acquired pneumonia. Curr Opin Crit Care.

2018;24(5):325-31.

125. Yang SS, Wang N-N, Postonogova T, Yang GJ, McGillion M, Beique F, et al. Intravenous lidocaine to prevent postoperative airway complications in adults: a systematic review and meta-analysis. Br J Anaesth. 1 mars 2020;124(3):314-23.

126. Jeong H-J, Lin D, Li L, Zuo Z. Delayed Treatment with Lidocaine Reduces Mouse Microglial Cell Injury and Cytokine Production After Stimulation with Lipopolysaccharide and Interferon γ. Anesth Analg. avr 2012;114(4):856-61.

127. Leng T, Gao X, Dilger JP, Lin J. Neuroprotective effect of lidocaine: is there clinical potential? Int J Physiol Pathophysiol Pharmacol. 25 avr 2016;8(1):9-13.

128. Matas M, Sotošek V, Kozmar A, Likić R, Sekulić A. Effect of local anesthesia with lidocaine on perioperative proinflammatory cytokine levels in plasma and cerebrospinal fluid in cerebral aneurysm patients: Study protocol for a randomized clinical trial. Medicine (Baltimore). oct 2019;98(42):e17450.

129. Badreldin HA, Atallah B. Global drug shortages due to COVID-19: Impact on patient care and mitigation strategies. Res Soc Adm Pharm RSAP. 19 mai 2020;

130. Jabaudon M, Boucher P, Imhoff E, Chabanne R, Faure J-S, Roszyk L, et al. Sevoflurane for Sedation in Acute Respiratory Distress Syndrome. A Randomized Controlled Pilot Study. Am J Respir Crit Care Med. 15 2017;195(6):792-800.

XX- ANNEXES

Annexe 1 : Questionnaire de santé SF-361 modifié (LidoCOVID)

A. Dans l'ensemble, pensez-vous que votre santé est : (entourez la bonne réponse)
   1. Excellente
   2. Très bonne
   3. Bonne
   4. Médiocre
   5. Mauvaise

B. Par rapport à l'année dernière à la même époque, comment trouvez-vous votre état de santé en ce moment ? (Entourez la réponse de votre choix)
   1. Bien meilleur que l'an dernier
   2. Plutôt meilleur
   3. A peu près pareil
   4. Plutôt moins bon
   5. Beaucoup moins bon

C. Au cours de la dernière semaine, et en raison de votre état physique (Entourez la réponse de votre choix, une par ligne)
   a. Avez-vous réduit le temps passé à votre travail ou à vos activités habituelles ?
      1. Oui
      2. Non
   b. Avez-vous accompli moins de choses que vous auriez souhaitées ?
      1. Oui
      2. Non
   c. Avez-vous du arrêter de faire certaines choses ?
      1. Oui
      2. Non
   d. Avez-vous eu des difficultés à faire votre travail ou toute autre activité? (Par exemple, cela vous a demandé un effort supplémentaire)
      1. Oui
      2. Non

D. Au cours de la dernière semaine, et en raison de votre état émotionnel (comme vous sentir triste, nerveux(se) ou déprimé(e)) (Entourez la réponse de votre choix, une par ligne)
   a. Avez-vous réduit le temps passé à votre travail ou à vos activités habituelles
      1. Oui
      2. Non
   b. Avez-vous accompli moins de choses que vous auriez souhaitées?
      1. Oui
      2. Non
   c. Avez-vous eu des difficultés à faire votre travail ou toute autre activité? (Par exemple, cela vous a demandé un effort supplémentaire)
      1. Oui
      2. Non

E. Au cours de la dernière semaine, dans quelle mesure votre état de santé, physique ou émotionnel, vous a-t-il gêné(e) dans votre vie sociale et vos relations avec les autres, votre famille, vos connaissances (Entourez la réponse de votre choix)
   1. Pas du tout
   2. Un petit peu
   3. Moyennement
   4. Beaucoup
   5. Enormément

F. Au cours de la dernière semaine, quelle a été l'intensité de vos douleurs (physiques)? (Entourez la réponse de votre choix)
   1. Nulle
   2. Très faible
   3. Faible
   4. Moyenne
   5. Grande
   6. Très grande

G. Au cours de la dernière semaine, dans quelle mesure vos douleurs physiques vous ont-elles limité(e) dans votre travail ou vos activités domestiques ? (Entourez la réponse de votre choix)
   1. Pas du tout
   2. Un petit peu
   3. Moyennement
   4. Beaucoup
   5. Enormément

H. Au cours de la dernière semaine, y a-t-il eu des moments où votre état de santé ou émotionnel, vous a gêné dans votre vie et vos relations avec les autres, votre famille, vos amis, vos connaissances ? (Entourez la réponse de votre choix)
   1. En permanence
   2. Une bonne partie du temps
   3. De temps en temps
   4. Rarement
   5. Jamais

I. Voici une liste d'activités que vous pouvez avoir à faire dans votre vie de tous les jours. Pour chacune d'entre elles indiquez si vous êtes limité(é) en raison de votre état de santé actuel. (Entourez la réponse de votre choix, une par ligne)
   a. Efforts physiques importants tels que courir, soulever un objet lourd, faire du sport
      1. Oui, beaucoup limité(e)
      2. Oui, un peu limité(e)
      3. Non, pas du tout limité(e)
   b. Efforts physiques modérés tels que déplacer une table, passer l'aspirateur, jouer aux boules
      1. Oui, beaucoup limité(e)
      2. Oui, un peu limité(e)
      3. Non, pas du tout limité(e)
   c. Soulever et porter des courses
      1. Oui, beaucoup limité(e)
      2. Oui, un peu limité(e)

3. Non, pas du tout limité(e)

d. Monter plusieurs étages par l'escalier
   1. Oui, beaucoup limité(e)
   2. Oui, un peu limité(e)
   3. Non, pas du tout limité(e)
e. Monter un étage par escalier
   1. Oui, beaucoup limité(e)
   2. Oui, un peu limité(e)
   3. Non, pas du tout limité(e)
f. Se pencher en avant, se mettre à genoux, s'accroupir
   1. Oui, beaucoup limité(e)
   2. Oui, un peu limité(e)
   3. Non, pas du tout limité(e)
g. Marcher plus d'un km à pied
   1. Oui, beaucoup limité(e)
   2. Oui, un peu limité(e)
   3. Non, pas du tout limité(e)
h. Marcher plusieurs centaines de mètres
   1. Oui, beaucoup limité(e)
   2. Oui, un peu limité(e)
   3. Non, pas du tout limité(e)
i. Marcher une centaine de mètres
   1. Oui, beaucoup limité(e)
   2. Oui, un peu limité(e)
   3. Non, pas du tout limité(e)
j. Prendre un bain, une douche ou s'habiller
   1. Oui, beaucoup limité(e)
   2. Oui, un peu limité(e)
   3. Non, pas du tout limité(e)

J. Les questions qui suivent portent sur comment vous vous êtes senti(e) au cours de ces 4 dernières semaines. Pour chaque question, veuillez indiquer la réponse qui vous semble la plus appropriée. Au cours de ces 4 dernières semaines, y a-t-il eu des moments où : (Entourez la réponse de votre choix, une par ligne)

a. Vous vous êtes senti(e) dynamique ?
   1. En permanence
   2. Très souvent
   3. Souvent
   4. Quelques fois
   5. Rarement
   6. Jamais
b. Vous vous êtes senti(e) très nerveux(se) ?
   1. En permanence
   2. Très souvent
   3. Souvent
   4. Quelques fois
   5. Rarement
   6. Jamais
c. Vous vous êtes senti(e) si découragé(e) que rien ne pouvait vous remonter le moral ?

1. En permanence
2. Très souvent
3. Souvent
4. Quelques fois
5. Rarement
6. Jamais

d. Vous vous êtes senti(e) calme et détendu(e) ?
   1. En permanence
   2. Très souvent
   3. Souvent
   4. Quelques fois
   5. Rarement
   6. Jamais

e. Vous vous êtes senti(e) débordant(e)d'énergie ?
   1. En permanence
   2. Très souvent
   3. Souvent
   4. Quelques fois
   5. Rarement
   6. Jamais

f. Vous vous êtes senti(e) triste et abattu(e) ?
   1. En permanence
   2. Très souvent
   3. Souvent
   4. Quelques fois
   5. Rarement
   6. Jamais

g. Vous vous êtes senti(e) épuisé(e) ?
   1. En permanence
   2. Très souvent
   3. Souvent
   4. Quelques fois
   5. Rarement
   6. Jamais

h. Vous vous êtes senti(e) heureux(e) ?
   1. En permanence
   2. Très souvent
   3. Souvent
   4. Quelques fois
   5. Rarement
   6. Jamais

i. Vous vous êtes senti(e) fatigué(e) ?
   1. En permanence
   2. Très souvent
   3. Souvent
   4. Quelques fois
   5. Rarement
   6. Jamais

K. Indiquez pour chacune des phrases suivantes dans quelle mesure elles sont vraies ou fausses dans votre cas : (Entourez la réponse de votre choix, une par ligne)

a. Je tombe malade plus facilement que les autres
   1. Totalement vrai
   2. Plutôt vrai
   3. Je ne sais pas
   4. Plutôt fausse
   5. Totalement fausse

b. Je me porte aussi bien que n'importe qui
   1. Totalement vrai
   2. Plutôt vrai
   3. Je ne sais pas
   4. Plutôt fausse
   5. Totalement fausse

c. Je m'attends à ce que ma santé se dégrade
   1. Totalement vrai
   2. Plutôt vrai
   3. Je ne sais pas
   4. Plutôt fausse
   5. Totalement fausse

d. Je suis en excellente santé
   1. Totalement vrai
   2. Plutôt vrai
   3. Je ne sais pas
   4. Plutôt fausse
   5. Totalement fausse

Score (0 à 100):

Annexe 2 : Débit lors de l'administration des traitements en ml/h

**Homme**								**Femme**						
**Taille (cm)**	**Poids idéal théorique (kg)**	**Bolus (ml)**	**H1 (ml/h)**	**H2 (ml/h)**	**de H3 à H24 (ml/h)**	**à partir de H25 (ml/h)**		**Taille (cm)**	**Poids idéal théorique (kg)**	**Bolus (ml)**	**H1 (ml/h)**	**H2 (ml/h)**	**de H3 à H24 (ml/h)**	**à partir de H25 (ml/h)**
		0,05 mL/kg	0,15 mL/kg/h	0,075 mL/kg/h	0,036 ml/kg/h	0,03 ml/kg/h				0,05 mL/kg	0,15 mL/kg/h	0,075 mL/kg/h	0,036 ml/kg/h	0,03 ml/kg/h
145	43,27	2,16	6,49	3,24	1,56	1,30		145	38,766	1,94	5,81	2,91	1,40	1,16
146	44,18	2,21	6,63	3,31	1,59	1,33		146	39,676	1,98	5,95	2,98	1,43	1,19
147	45,09	2,25	6,76	3,38	1,62	1,35		147	40,586	2,03	6,09	3,04	1,46	1,22
148	46,00	2,30	6,90	3,45	1,66	1,38		148	41,496	2,07	6,22	3,11	1,49	1,24
149	46,91	2,35	7,04	3,52	1,69	1,41		149	42,406	2,12	6,36	3,18	1,53	1,27
150	47,82	2,39	7,17	3,59	1,72	1,43		150	43,316	2,17	6,50	3,25	1,56	1,30
151	48,73	2,44	7,31	3,65	1,75	1,46		151	44,226	2,21	6,63	3,32	1,59	1,33
152	49,64	2,48	7,45	3,72	1,79	1,49		152	45,136	2,26	6,77	3,39	1,62	1,35
153	50,55	2,53	7,58	3,79	1,82	1,52		153	46,046	2,30	6,91	3,45	1,66	1,38
154	51,46	2,57	7,72	3,86	1,85	1,54		154	46,956	2,35	7,04	3,52	1,69	1,41
155	52,37	2,62	7,85	3,93	1,89	1,57		155	47,866	2,39	7,18	3,59	1,72	1,44
156	53,28	2,66	7,99	4,00	1,92	1,60		156	48,776	2,44	7,32	3,66	1,76	1,46
157	54,19	2,71	8,13	4,06	1,95	1,63		157	49,686	2,48	7,45	3,73	1,79	1,49
158	55,10	2,75	8,26	4,13	1,98	1,65		158	50,596	2,53	7,59	3,79	1,82	1,52
159	56,01	2,80	8,40	4,20	2,02	1,68		159	51,506	2,58	7,73	3,86	1,85	1,55
160	56,92	2,85	8,54	4,27	2,05	1,71		160	52,416	2,62	7,86	3,93	1,89	1,57
161	57,83	2,89	8,67	4,34	2,08	1,73		161	53,326	2,67	8,00	4,00	1,92	1,60
162	58,74	2,94	8,81	4,41	2,11	1,76		162	54,236	2,71	8,14	4,07	1,95	1,63

163	59,65	2,98	8,95	4,47	2,15	1,79		163	55,146	2,76	8,27	4,14	1,99	1,65
164	60,56	3,03	9,08	4,54	2,18	1,82		164	56,056	2,80	8,41	4,20	2,02	1,68
165	61,47	3,07	9,22	4,61	2,21	1,84		165	56,966	2,85	8,54	4,27	2,05	1,71
166	62,38	3,12	9,36	4,68	2,25	1,87		166	57,876	2,89	8,68	4,34	2,08	1,74
167	63,29	3,16	9,49	4,75	2,28	1,90		167	58,786	2,94	8,82	4,41	2,12	1,76
168	64,20	3,21	9,63	4,81	2,31	1,93		168	59,696	2,98	8,95	4,48	2,15	1,79
169	65,11	3,26	9,77	4,88	2,34	1,95		169	60,606	3,03	9,09	4,55	2,18	1,82
170	66,02	3,30	9,90	4,95	2,38	1,98		170	61,516	3,08	9,23	4,61	2,21	1,85
171	66,93	3,35	10,04	5,02	2,41	2,01		171	62,426	3,12	9,36	4,68	2,25	1,87
172	67,84	3,39	10,18	5,09	2,44	2,04		172	63,336	3,17	9,50	4,75	2,28	1,90
173	68,75	3,44	10,31	5,16	2,47	2,06		173	64,246	3,21	9,64	4,82	2,31	1,93
174	69,66	3,48	10,45	5,22	2,51	2,09		174	65,156	3,26	9,77	4,89	2,35	1,95
175	70,57	3,53	10,58	5,29	2,54	2,12		175	66,066	3,30	9,91	4,95	2,38	1,98
176	71,48	3,57	10,72	5,36	2,57	2,14		176	66,976	3,35	10,05	5,02	2,41	2,01
177	72,39	3,62	10,86	5,43	2,61	2,17		177	67,886	3,39	10,18	5,09	2,44	2,04
178	73,30	3,66	10,99	5,50	2,64	2,20		178	68,796	3,44	10,32	5,16	2,48	2,06
179	74,21	3,71	11,13	5,57	2,67	2,23		179	69,706	3,49	10,46	5,23	2,51	2,09
180	75,12	3,76	11,27	5,63	2,70	2,25		180	70,616	3,53	10,59	5,30	2,54	2,12
181	76,03	3,80	11,40	5,70	2,74	2,28		181	71,526	3,58	10,73	5,36	2,57	2,15
182	76,94	3,85	11,54	5,77	2,77	2,31		182	72,436	3,62	10,87	5,43	2,61	2,17
183	77,85	3,89	11,68	5,84	2,80	2,34		183	73,346	3,67	11,00	5,50	2,64	2,20
184	78,76	3,94	11,81	5,91	2,84	2,36		184	74,256	3,71	11,14	5,57	2,67	2,23
185	79,67	3,98	11,95	5,97	2,87	2,39		185	75,166	3,76	11,27	5,64	2,71	2,25
186	80,58	4,03	12,09	6,04	2,90	2,42		186	76,076	3,80	11,41	5,71	2,74	2,28
187	81,49	4,07	12,22	6,11	2,93	2,44		187	76,986	3,85	11,55	5,77	2,77	2,31
188	82,40	4,12	12,36	6,18	2,97	2,47		188	77,896	3,89	11,68	5,84	2,80	2,34
189	83,31	4,17	12,50	6,25	3,00	2,50		189	78,806	3,94	11,82	5,91	2,84	2,36
190	84,22	4,21	12,63	6,32	3,03	2,53		190	79,716	3,99	11,96	5,98	2,87	2,39

191	85,13	4,26	12,77	6,38	3,06	2,55		191	80,626	4,03	12,09	6,05	2,90	2,42
192	86,04	4,30	12,91	6,45	3,10	2,58		192	81,536	4,08	12,23	6,12	2,94	2,45
193	86,95	4,35	13,04	6,52	3,13	2,61		193	82,446	4,12	12,37	6,18	2,97	2,47
194	87,86	4,39	13,18	6,59	3,16	2,64		194	83,356	4,17	12,50	6,25	3,00	2,50
195	88,77	4,44	13,31	6,66	3,20	2,66		195	84,266	4,21	12,64	6,32	3,03	2,53
196	89,68	4,48	13,45	6,73	3,23	2,69		196	85,176	4,26	12,78	6,39	3,07	2,56
197	90,59	4,53	13,59	6,79	3,26	2,72		197	86,086	4,30	12,91	6,46	3,10	2,58
198	91,50	4,57	13,72	6,86	3,29	2,74		198	86,996	4,35	13,05	6,52	3,13	2,61
199	92,41	4,62	13,86	6,93	3,33	2,77		199	87,906	4,40	13,19	6,59	3,16	2,64
200	93,32	4,67	14,00	7,00	3,36	2,80		200	88,816	4,44	13,32	6,66	3,20	2,66
201	94,23	4,71	14,13	7,07	3,39	2,83		201	89,726	4,49	13,46	6,73	3,23	2,69
202	95,14	4,76	14,27	7,14	3,42	2,85		202	90,636	4,53	13,60	6,80	3,26	2,72
203	96,05	4,80	14,41	7,20	3,46	2,88		203	91,546	4,58	13,73	6,87	3,30	2,75
204	96,96	4,85	14,54	7,27	3,49	2,91		204	92,456	4,62	13,87	6,93	3,33	2,77
205	97,87	4,89	14,68	7,34	3,52	2,94		205	93,366	4,67	14,00	7,00	3,36	2,80

Annexe 3 : PRISE EN CHARGE DES PATIENTS COVID-19 aux Hôpitaux Universitaires de Strasbourg ADAPTEE DES RECOMMANDATIONS DU HCSP ET COREB

	N° d'indexation : RP 90 / 02	PROPOSITIONS THERAPEUTIQUES COVID-19

PRISE EN CHARGE DES PATIENTS COVID-19
ADAPTEE DES RECOMMANDATIONS DU HCSP ET COREB

NATURE DE LA RECOMMANDATION :

☐ Recommandation issue des mentions de l'AMM ou de l'ATU	☑ Recommandation spécifique aux H.U.S.	☐ Recommandation de prescription à certains services de soins	☐ Recommandation de prescription à certains prescripteurs	☑ Recommandation d'indications

CONTENU DE LA RECOMMANDATION :

Ce document se base sur les données de la littérature au 29 octobre 2020. Il s'agit d'un état des connaissances sur quelques points de prise en charge, **à adapter à chaque patient et à chaque situation clinique**. Ce document sera modifié selon l'évolution des connaissances. Dans la mesure du possible, il est souhaitable de proposer aux patients des inclusions dans les essais thérapeutiques auxquels participent les HUS. Un protocole DX-Care est disponible.

TRAITEMENT ANTIVIRAL

- **Cas général : pas de traitement antiviral**.
  - Inefficacité démontrée pour (hydroxy)-chloroquine (Plaquenil®), lopinavir-ritonavir (Kaletra®), azithromycine.
  - Remdésivir : à ce jour, à réserver uniquement à des situations spécifiques en hospitalisation conventionnelle après discussion multi-disciplinaire (cf. contacts ci-dessous).

ANTIBIOTHERAPIE

- **Cas général : pas d'antibiothérapie systématique**, même en cas de corticothérapie.
- Uniquement en cas de gravité ou de pneumopathie bactérienne suspectée : amoxicilline-acide clavulanique ou cefotaxime ou ceftriaxone ± spiramycine pendant 5 jours. Voir note de la CAI/COMEDIMS

CORTICOTHERAPIE

- **Patient non oxygéno-requérant : pas de corticothérapie.**
- **Patient oxygéno-requérant** :
  - Dexaméthasone 6 mg/j IV (10 mg/j si poids > 100 kg).
  - Alternatives :
    Methylprednisolone : 32 mg/j
    Prednisone/Prednisolone 40 mg/j per os (60 mg/j si poids > 100 kg)
  - Durée 7 à 10 jours. Privilégier la voie orale chaque fois que cela est possible, d'emblée ou en relais de l'IV
  - Arrêt en cas de sortie d'hospitalisation.
  - ***Surveillance glycémique et ionogramme***, ivermectine dose unique en cas d'exposition à risque.
  - ***Pour les patients > 70 ans et femmes enceintes***, évaluation du rapport bénéfice/risque individuel.

TRAITEMENT IMMUNO-MODULATEUR

- **Cas général : pas de traitement immuno-modulateur.**
  - Tocilizumab : uniquement dans le cadre d'un protocole thérapeutique (essai clinique).
  - Plasma de convalescent : uniquement dans des situations très spécifiques, à valider par la RCP nationale.

ANTICOAGULATION

- **Patient non oxygéno-requérant** : anticoagulation préventive standard (enoxaparine 4000 UI 1x/j ou 6000 UI 1x/j si poids > 120 kg) si réduction de mobilité et facteurs de risque de maladie veineuse thromboembolique.
- **Patient oxygéno-requérant** :
  - Anticoagulation préventive **standard** en l'absence de facteurs de risque (cf. supra)
  - Anticoagulation **préventive renforcée** (enoxaparine 4000 UI 2x/j ou 6000 UI 2x/j si poids > 120 kg) si FDR thromboembolique (ex : syndrome inflammatoire important, IMC >30 kg/m², ventilation mécanique)
  - Anticoagulation **curative** selon critères cliniques et biologiques, durée adaptée au contexte clinique (exemple : survenue d'un évènement thromboembolique)
  - ***Evaluer le risque thrombotique et hémorragique individuel (ATCD, traitements médicamenteux) ; adaptation thérapeutique selon la fonction rénale, arrêt si sortie d'hospitalisation ou indication spécifique.***

ISOLEMENT ET ORIENTATION DES PATIENTS

- Page Intra HUS de l'Equipe Opérationnelle d'Hygiène
- Outil d'aide à l'orientation des patients COVID en sortie d'hôpital

Pour toute question ou demande d'information :
Questionnaire « avis infectieux » (Dx-Care), seniorsSMIT@chru-strasbourg.fr ; Pharmacie-contact@chru-strasbourg.fr

**Création :**		**Modification :**		**Validation :**	
**Le : 29/10/2020**	**Service des maladies infectieuses**	**Le : 04/11/20**	**Par : Groupe HUS**	**Le : 05/11/20**	**CAI et COMEDIMS**

Page 1/1
